# Metal–support interactions in metal oxide-supported atomic, cluster, and nanoparticle catalysis†

**DOI:** 10.1039/d4cs00527a

**Published:** 2024-10-02

**Authors:** Denis Leybo, Ubong J. Etim, Matteo Monai, Simon R. Bare, Ziyi Zhong, Charlotte Vogt

**Affiliations:** a Schulich Faculty of Chemistry, and Resnick Sustainability Center for Catalysis, Technion, Israel Institute of Technology, Technion City Haifa 32000 Israel C.Vogt@technion.ac.il; b Department of Chemical Engineering and Guangdong Provincial Key Laboratory of Materials and Technologies for Energy Conversion (MATEC), Guangdong Technion Israel Institute of Technology (GTIIT) 241 Daxue Road Shantou 515063 China; c Inorganic Chemistry and Catalysis group, Institute for Sustainable and Circular Chemistry, Utrecht University, Universiteitsweg 99 3584 CG Utrecht The Netherlands; d Stanford Synchrotron Radiation Lightsource, SLAC National Accelerator Laboratory 2575 Sand Hill Road Menlo Park CA 94025 USA; e SUNCAT Center for Interface Science and Catalysis, SLAC National Accelerator Laboratory Menlo Park CA 94025 USA

## Abstract

Supported metal catalysts are essential to a plethora of processes in the chemical industry. The overall performance of these catalysts depends strongly on the interaction of adsorbates at the atomic level, which can be manipulated and controlled by the different constituents of the active material (*i.e.*, support and active metal). The description of catalyst activity and the relationship between active constituent and the support, or metal–support interactions (MSI), in heterogeneous (thermo)catalysts is a complex phenomenon with multivariate (dependent and independent) contributions that are difficult to disentangle, both experimentally and theoretically. So-called “strong metal–support interactions” have been reported for several decades and summarized in excellent review articles. However, in recent years, there has been a proliferation of new findings related to atomically dispersed metal sites, metal oxide defects, and, for example, the generation and evolution of MSI under reaction conditions, which has led to the designation of (sub)classifications of MSI deserving to be critically and systematically evaluated. These include dynamic restructuring under alternating redox and reaction conditions, adsorbate-induced MSI, and evidence of strong interactions in oxide-supported metal oxide catalysts. Here, we review recent literature on MSI in oxide-supported metal particles to provide an up-to-date understanding of the underlying physicochemical principles that dominate the observed effects in supported metal atomic, cluster, and nanoparticle catalysts. Critical evaluation of different subclassifications of MSI is provided, along with discussions on the formation mechanisms, theoretical and characterization advances, and tuning strategies to manipulate catalytic reaction performance. We also provide a perspective on the future of the field, and we discuss the analysis of different MSI effects on catalysis quantitatively.

## Abstract 2

Metal–support interactions (MSI) impact catalyst activity, stability, and selectivity. This review critically evaluates recent findings, theoretical advances, and MSI tuning strategies, offering new perspectives for future research in the field.

## Graphical abstract

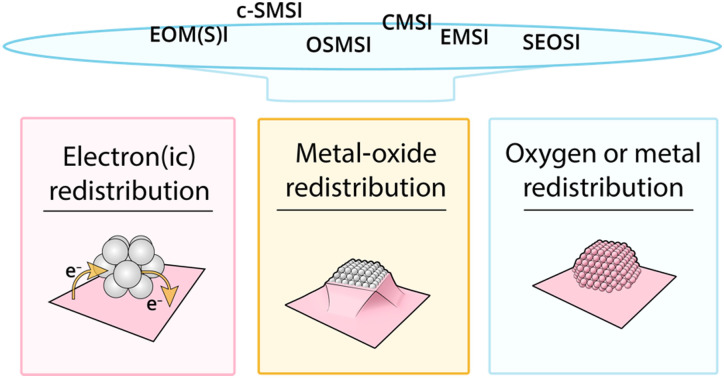

## 1. Introduction

**Fig. 1:**
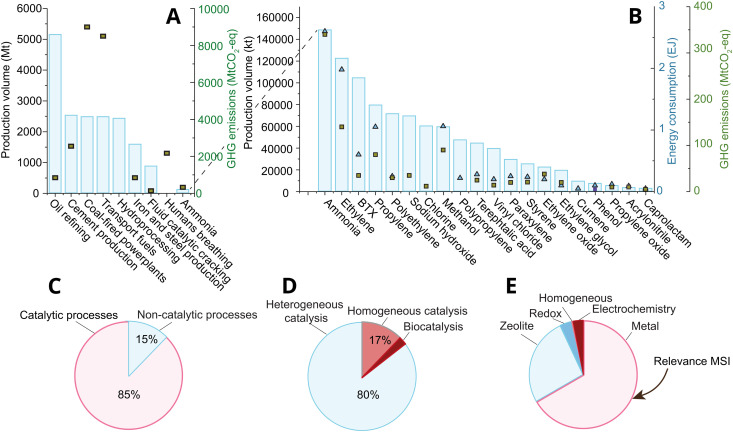
(A) and (B) The production volume of major chemical and catalytic processes in Mt, and their greenhouse gas emissions in Mt of CO_2_-eq. The bars represent the production volume, while the square markers represent the GHG emissions that stem directly from the process.^6^ (B) Zoom-in of (A) on the 20 most-produced chemicals by volume. The market size of the chemical industry was roughly 4.7 trillion USD in 2021. Reproduced with permission from ref. 7 Copyright 2021, American Chemical Society. (C) The percentage of all industrial processes that entail the use of catalytic processes.^1,8,9^ Reproduced with permission from ref. 1. Copyright 2009, John Wiley and Sons. (D) The division of catalytic processes into hetero-, homo-, and biocatalysis.^1,8,9^ Reproduced with permission from ref. 1. Copyright 2009, John Wiley and Sons. (E) Catalyst types (classifications used explicated in ref. 5) by volume of process used to produce the chemicals shown in (B), and including fluid catalytic cracking and hydroprocessing from (A).

Catalysis lies at the heart of the chemical industry, accounting for over 85% of processes^1,2^ within the roughly 4.7 trillion USD (TUSD/y) global market of 2021^3^ (Fig. 1(A–C)); yet catalysis is believed to indirectly affect over a third of the world's 80 TUSD/y global gross domestic product, and roughly 80% of the energy demand, and 75% of all greenhouse gas emissions.^4^ Roughly 80% of industrial catalysis is heterogeneous thermocatalysis (Fig. 1D), where the product and reactant are in a different physical state than the catalyst (often liquid or gas *versus* the solid catalyst) due to relatively simplistic process constraints that nevertheless dominate process economics, like ease of product and catalyst separation, and stability under reaction conditions. The two most abundantly applied classes of heterogeneous thermocatalysts (following the definitions given in ref. 5) in the industry are supported metal, and solid acid catalysts (zeolites and zeotypes). Of these, supported metal catalysts are the most broadly applied in terms of both volume and number of high volume processes, as shown in Fig. 1E.

Active metals (AM) are dispersed on supports primarily to increase the ratio of reactive surface per unit of AM, with the metal present as dispersed nanoparticles (NPs). High surface area supports are often used to create large specific surface areas of the AM component within small volumes of sample, and help to prevent the aggregation of the supported species during both preparation and catalytic use, resulting in improved dispersion and stability. The performance of supported metal catalysts of a particular type of metal usually depends on several parameters, which can broadly and generally be summarized under electronic, geometric, and confinement effects, which are often difficult to disentangle, especially taking into account their constant change as a function of time on reactants stream.^5,10^ Displays of such effects can be found by, for example, varying the particle size,^11–13^ varying the close environment of the reactive metal,^14^ and by varying metal–support interaction (MSI). This provides endless degrees of possible combinations and variations of materials, geometries, and environments.^15,16^

Ideally, one should be able to carefully select catalytic building blocks with specific electronic, geometric, and even confinement properties at the atomic scale to produce the right MSI and the most active, stable, and selective catalyst. However, due to the scale and complexity of the system presented, it is a long-standing challenge to disentangle the contributions of MSI effects and to unify the physicochemical principles that dominate their broad range. Many excellent efforts have been made in this regard. Yet, the introduction of several new terms relating to MSI in recent literature provides an opportunity for an updated overview, with particular attention to size-dependent effects in metal oxide-supported metal catalysis, in an attempt to holistically understand supported catalysts. Before analyzing MSI in catalysis, we first describe some generalized properties of metal oxide (MO) supports, define size-dependent properties of the AM constituents, and relate reactant (adsorbate) properties to them.

Typically, used MO supports have greatly varying physical and chemical properties, from wide-gap insulators to semiconductors, depending on their band gap. Broadly, MO supports can be subdivided into those that are popularly termed “non-reducible” (for example, Al_2_O_3_, SiO_2_, ZrO_2_) and “reducible” (for example, TiO_2_, CeO_2_), and this subdivision greatly dictates the expected MSI as will be further detailed in Section 3.3, where we will analyze this concept of “reducibility” and its relationship to free energy and the electronic structure of metal oxides. Bulk material properties of the MO support can be drastically altered, for example, through doping or nanostructuring, and we refer the reader to an excellent review on the topic for an in-depth discussion.^17^ The bonding of AM atoms to non-reducible, non-defective supports is typically covalent,^18^ while bonding on reducible supports can be both covalent and ionic.^19^

**Fig. 2:**
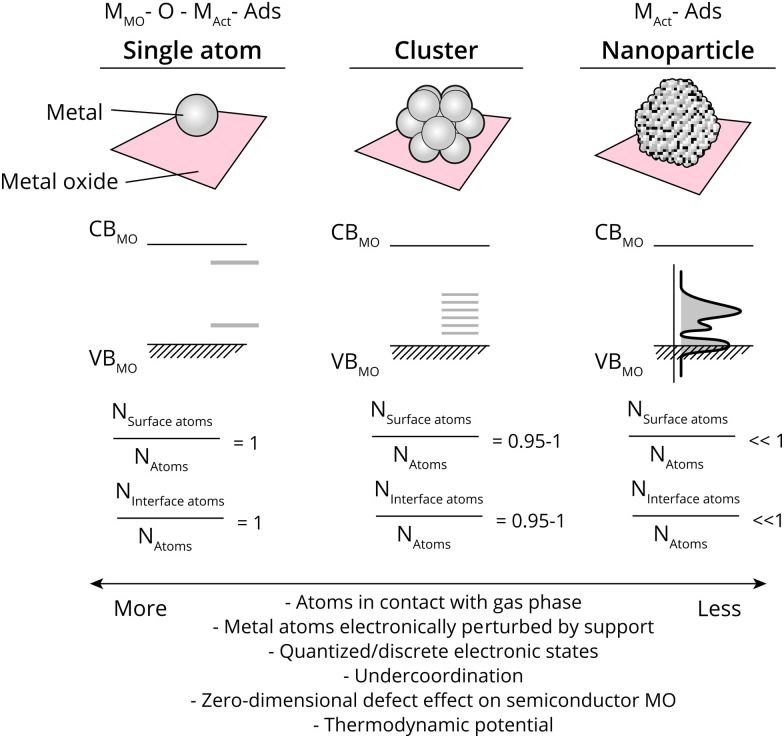
Schematic overview of geometric and electronic effects in supported metal single atoms, clusters, and nanoparticles; the relationship between the conduction band and valence band of the metal oxide (CBMO and VBMO, respectively), the ratio of exposed surface atoms *versus* total atoms (N_Surface atoms_/N_Atoms_), the involved schematic description of the adsorbates can either be simplified as strongly correlated to the metal of the metal oxide support, an oxygen atom, and the metal constituting the ‘active site’ (metal of metal oxide (MMO), O, and active metal (MAct), respectively) for ‘single atoms’, or to the bulk MAct.

The ratio of surface-exposed AM atoms (*i.e.*, the ratio of the AM constituent that is exposed to reactants), and interfacial AM atoms (*R*_surf_, also called dispersion, and *R*_int_, respectively) relative to all AM atoms further dictates several properties related closely to catalysis. The value of *R*_surf_ and *R*_int_ varies from close to 0 to 1 when going from large metal NPs to single atoms. Values of *R*_surf_ and *R*_int_ closer to 1 mean that more atoms are in contact with the reactant phase, a higher degree of metal under-coordination, and a larger relative electronic perturbation from the MO, among others (see Fig. 2). As will be alluded to in detail in this work, metal NPs undergo polarization due to MSI at metal/semiconductor interfaces to varying extents, based on the differences in energetics of the NPs and MO used, while single metal atoms can be described as zero-dimensional (point) defects within a semiconductor MO, where the difference between the band edge and the AM state affect the binding energy with reactants and intermediates, and thus catalytic properties. The strength and nature of the interaction between AM and MO are particularly crucial at *R*_surf_ and *R*_int_ ≪ 1. Especially in such cases, it is possible that a certain AM on a specific bulk MO support can have entirely different catalytic properties depending on the type of interaction it has with the surface, for example dictated by the type of defect site the atom is located on; through covalent or ionic bonding and whether interacting with anions, or cations of the support.

Adsorbates in a sequence of elementary reaction steps also interact differently with surface metal atoms depending on the type of orbital involved in binding, *i.e.*, σ *vs*. π. While σ-bonds preferentially bind to and are activated by highly undercoordinated atoms (abundant for *R*_surf_ close to 1), π-bonds energetically favor more coordinated binding sites, where a larger degree of surface interaction can stabilize the transition state and favor bond cleavage.^20^ Perhaps such interaction could also be achieved by low energetic differences between the MO band edge and AM state at *R*_surf_ and *R*_int_ close to 1.

The term MSI is used to classify any noted effect on catalytic activity, stability, or selectivity that can be attributed to interactions of the AM with the support – which means that all supported metal catalysts experience MSI, to greater or lesser extents.

Indeed, this very broad possible use of the term MSI has led to a wide variety of its subclassifications in different systems. Table 1 gives an overview of the terminologies and abbreviations, the postulated main underlying physicochemical principles, typical examples, and our best attempt to note their first reports in the literature.

**Table 1:** Overview of the different variations of MSI terminology from the literature and the underlying physical principles that characterize them. Abbreviations used in the table: supported metal nanoparticles (SMNPs), supported metal clusters (SMCs), supported metal oxide nanoparticles (SMONPs), oxide metal inverse catalysts (OMICs), single atom catalysts (SACs)

Abbreviation	Term	Main observation and underlying physical principle(s)	Catalyst system	Typical examples	First report
c-SMSI	Classical strong metal–support interaction	Suppressed adsorption of small molecules (CO/H_2_), suboxide overlayer formation under high-temperature H_2_ reduction	SMNPs	Group VIII noble metals/TiO_2_	21
EMSI	Electronic metal–support interaction	Electronic perturbation, often for smaller clusters to atoms, through metal-to-oxide or oxide-to-metal charge transfer, formation of covalent/ionic bonds	SMNPs, SACs, SMCs	Pt/TiO_2_, metal clusters, CeO_*x*_/Cu(111)	22 and 23
CMSI	Covalent metal–support interaction	Strong covalent bond formation, prevalent in single atom catalysts	SACs	Au_1_/FeO_*x*_	24 and 25
OSMSI	Oxidative strong metal–support interaction	Suppressed adsorption due to overlayer formation, similar to c-SMSI, but onset by low temperature oxidation	SMNPs	Au/ZnO, Pt-group/ZnO	26
EOMI	Electronic oxide metal–support interaction	Metal-to-oxide charge transfer (from metal substrates to supported MO adlayer), mainly on model catalysts where the metal is used as support	OMICs	CeO_*x*_/Cu(111)	24
				CeO_*x*_/Ag(111)	
EOMSI	Electronic oxide metal strong interaction	Metal-to-oxide charge transfer (from metal substrates to supported MO adlayer), formation of thin oxide adlayers containing low-valent metal cations	OMICs	CeO_*x*_/Cu(111)	24 and 27
SEOSI	Strong electronic oxide-support interaction	Charge transfer from support oxide to oxide catalyst	SMONPs	In_2_O_3_/ZrO_2_	28
A-SMSI	Adsorbate-mediated strong metal–support interaction	Functionalized suboxide overlayer formation under moderate temperature H_2_ reduction	SMNPs	Rh/TiO_2_, Rh/Nb_2_O_5_	29 and 30
WC-SMSI	Wet-chemical strong metal–support interaction	Suboxide overlayer formation as a result of treatment of supported metal nanoparticles with corresponding cation solution at room temperature and subsequent moderate temperature heat treatment	SMNPs	Au/TiO_2_	31

The diversification of terminology in the literature stems from our more advanced understanding of classical strong metal–support interaction (c-SMSI), which is a result of the increasing availability of theoretical and analytical tools, and synthesis capabilities, through which the electronic and geometric effects can be studied in more detail. For example, one could argue that metal NPs, as opposed to nanoclusters or atoms (*i.e.*, those larger than approximately 2.5 nm diameter), in most cases, exhibit what we classify as “c-SMSI” (see Table 1), characterized by the decoration of the NPs with suboxide species. On the other hand, metal clusters (*i.e.*, those smaller than approximately 2.5 nm) are more associated with electronic metal–support interaction (EMSI) characterized by electronic perturbation, for example, by metal-to-oxide or oxide-to-metal charge transfer, with the partial charge per atom noted to reach a maximum with 30–70 atoms for a Pt/CeO_2_ system.^32,33^ The interactions that have gained much recent interest in supported “single atom catalysts” (SACs) were described as covalent metal–support interaction (CMSI), when strong covalent bonds are at play.^34^

In fact, as will be discussed in more detail in Section 7.3, most of the above-noted variations in nomenclature stem from the same underlying physical concept, *i.e.*, the strength and type of electronic interaction, which is often quite difficult to characterize and warrants a critical or cautious approach in employing them. In any case, whether or not there is an effect that dominates the observed behavior, different contributions are generally simultaneously at play in such supported metal catalyst systems. For example, in a SAC system where CMSI is believed to dominate by characterization of the catalyst *ex situ*, EMSI can also play a large role when adsorbates are introduced^33,35^ (an added complexity is in the presence of clusters or small NPs co-existing in many SAC systems^36^ where even decoration, c-SMSI, can perhaps take place). Taking all of this into account, we believe that the use of specific MSI subclassifications is problematic, as it is likely misleading despite their widespread use.

Since the discovery of the c-SMSI phenomenon in the 1970s, several excellent review papers have been published discussing the (c-S)MSI of supported metal/metal oxide catalysts from inception to date.^15,16,27,34,37–49^ We refer readers, for example, to a review by De Jong and co-workers for a comprehensive and systematic discussion of c-SMSI aimed at controlling catalytic properties.^16^ Recently, Xie *et al.*^49^ discussed c-SMSI as a double-edged sword on the catalytic activity of CO_2_ hydrogenation to *C*_1_ value-added molecules. These reviews have helped us to dissect and explicate differences in MSI of oxide-supported metal catalysts, with a particular focus on clarifying and unifying the recent literature and trends in it that have not yet received attention. We pay special attention to the ways to generate and control MSI and characterize MSI-related materials properties through the prism of current state theory of MSI phenomena. We finally provide paths for new research and suggest possible guiding principles to deploy the various concepts of MSI to design novel catalyst systems, such as dynamic restructuring under alternating redox and reaction conditions, and adsorbate-induced MSI.

## 2. The history of MSI in catalysis

The possibility of catalyst supports interacting with or somehow modifying the properties of metal particles has long been recognized.^50,51^ The term “synergetic promotion” by the support to describe changes in activation energy of a reaction on supported catalysts was introduced by Schwab as early as 1930.^52^ By the 1970s, it was known that solid acids could interact strongly with metal crystallites, withdrawing electron density from them and leaving an electron-deficient metal particle, while basic supports could increase the metal's electron density.^21,53^

In the late 1970s, Tauster *et al.*^21,54^ reported on Group VIII metal particles supported on TiO_2_, which, upon high-temperature reductive treatment, led to an observed suppression of CO and H_2_ chemisorption. They postulated that a special chemical interaction between the metal particles and the support occurred, which they termed “strong metal–support interactions” (SMSI). This term was then proposed to describe the electron transfer between AM NPs and the support, which was later justified by spectroscopic studies (X-ray photoelectron spectroscopy (XPS), electron energy loss spectroscopy (EELS)).^41^ This explanation, however, was later criticized in the literature,^55–57^ and a new model that attributed observed drastic changes in the adsorption properties of a catalyst to the migration of a species originating from the support over the surface of the metal particles was proposed.^58^ The first direct evidence of overlayer formation characterizing c-SMSI was made available in a study by Braunschweig *et al.*^59^ in 1989 with the assistance of high-resolution transmission electron microscopy (HRTEM), which showed the presence of amorphous overlayers of support material on both Rh particles and TiO_2_ support after high-temperature reduction. Sequential oxidation and reduction at low temperature partially removed the overlayers. Later, fundamental studies on MSI took center stage, where several studies were published utilizing different reducible supports,^43,54^ including CeO_2_, Nb_2_O_5_, Fe_3_O_4_, and non-oxide supports,^60^ focused on the understanding of formation mechanisms^61–64^ and subsequent catalytic effects of c-SMSI.^65–67^

**Fig. 3:**
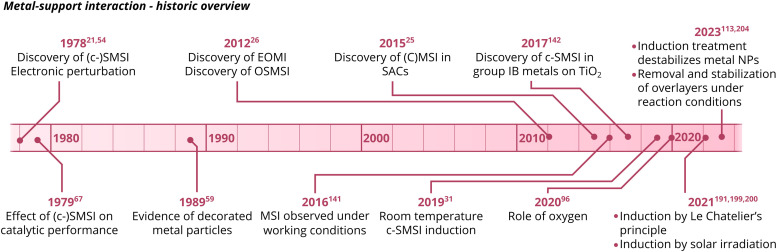
A schematic overview of key MSI-related observations.

In recent years, intense interest in MSI has re-emerged in the academic community. Consequently, the subject has been investigated in increasing detail and in a broad variety of systems. Several key findings have been published in the last 5–10 years, and the important MSI-related observations are summarized in Fig. 3.

The evolution of new observations in literature was accompanied by the introduction of new terms that emphasized different aspects of the phenomena. Due to the diversity of different parameters affecting MSI, this led to a broad terminology, which focused mainly on synthetic subtleties or size effects rather than on underlying fundamental principles, *vide infra*.

For example, it was shown that, despite the classical view, SMSI could also occur in oxidative environments for some types of systems,^26,68,69^ a phenomenon which was termed oxidative strong metal–support interaction (OSMSI), placing the accent on the pretreatment environment. It was further found that MSI comprises interfacial charge-transfer/redistribution and not only material transport through the AM/MO interface.^70^ To emphasize that, the term EMSI was introduced, which was also refined after SACs became popular in which the CMSI term is used to describe MSI more often, since charge transfer in SACs is of more localized character with the formation of covalent bonds.^25^ Charge transfer between two metal oxides (one active and one performing the role of the support) was also realized to occur and was called strong electronic oxide–support interaction (SEOSI).^28^ Moreover, charge and mass transfer at the metal/support interface was studied using model “inverse” catalysts, in which oxide NPs are deposited on well-defined crystallographic planes of metals.^24,27^ Although these systems are not considered to be applicable on an industrial scale and are used for model studies, the new terms, electronic oxide metal–support interaction (EOMI)/electronic oxide metal strong interaction (EOMSI), were introduced to designate MSI effects in these classes of materials.

It is clear that the current terminology lacks consistency and can be misleading, making it difficult to accurately describe the connections between catalyst composition, the properties of its constituents, and the catalyst's activity, selectivity, and stability. First, we must understand the fundamental principles, the underlying physical and chemical processes, that will allow us to group different MSI phenomena and consequently to get closer to the rational design of catalytic materials.

## 3. Fundamentals of MSI

The term MSI should classify any beneficial or non-beneficial electronic, geometrical, enthalpic, or entropic effect on catalytic activity, stability, or selectivity that can be attributed to resulting interactions of an AM component with a support. Such effects arise from thermodynamic driving forces leading to a redistribution of electrons between the metal and MO to reach electronic equilibrium and to the migration of atoms to form more stable surface structures such as overlayers or alloys. Accordingly, AM–MO interactions can be predominantly electronic in nature, or they may be most clearly observed due to geometric rearrangements, but the two are invariably linked.^5^ The size of metal NPs on a support, their wetting behavior, resulting geometry, sintering resistance, propensity towards phase transformations, and restructuring may all be explained by a combination of these effects. Before discussing the intricate, complex and often convoluted characteristics that can result from metal–support interactions, we will analyze AM/MO systems by gradually building up complexity towards reality.

### 3.1. Electronic redistribution

#### 3.1.1. Bulk metal–metal oxide electronic interaction

We begin the description of MSI phenomena by the consideration of a simple system consisting of an AM and an MO support in which we consider initially only their differing bulk electronic structures and the resulting electronic equilibration upon contact between them. In this case, the description of MSI reduces to electronic interaction, *i.e.*, charge transfer at the metal/support interface (for metal oxides, this would be a metal–semiconductor interaction in the case of *e.g.*, TiO_2_, or metal–insulator interaction in the case of, *e.g.*, SiO_2_).

**Fig. 4:**
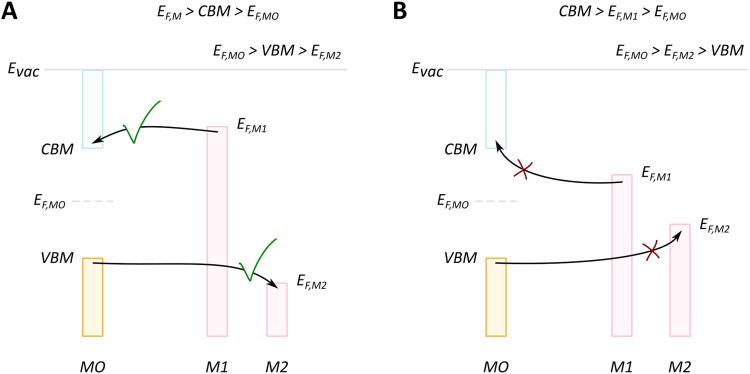
Different relationships between key electronic characteristics of metal and MO, and corresponding direction and possibility of charge flow. In case of the Fermi level of the metal is positioned higher in energy than that of the MO, the electron flow is supposed to be from the metal to the MO (case M1 in (A) and (B)), while the reverse direction is favorable in the opposite case of Fermi levels distribution. Due to the existence of a forbidden energy range in the electronic structure of the MO, the band gap, only transitions as those illustrated in the case of (A) are allowed. MO – metal oxide, M1 and M2 – metals, VBM – valence band maximum, CBM – conduction band minimum, *E*_vac_ – position of vacuum level, *E*_F,MO_ – position of Fermi level of MO, *E*_F,M_ – position of Fermi level of M1 or M2.

We will discuss first the simplistic and unrealistic case of a pristine system with bulk band structures, as illustrated in Fig. 4. The direction of charge redistribution is principally governed by the initial arrangement of the Fermi level of the metal (*E*_F,M_) and that of the oxide (*E*_F,MO_), whose position is in the middle of its band gap in this case. Since the Fermi level of a material equates to the thermodynamic potential of its electrons, after contact between the two materials, a favorable direction of electron flow will be established from the material with a higher Fermi level position to the one with a lower Fermi level position. The existence of the band gap in the MO imposes additional restrictions. A high band gap value, such as typical for MO supports used in catalysis, implies that thermal energy is not sufficient for an electron to overcome the energy barrier that is the band gap, following from the Fermi–Dirac distribution and the Arrhenius equation for carrier concentration^71^ at standard conditions, which results in close to zero electron density in the conduction band and a nominally full valence band. As a consequence, the only way for an electron to move from the metal with a higher Fermi level to the MO is to its conduction band. This transition is allowed only if *E*_F,M_ is higher than the conduction band minimum (CBM) of the MO, Fig. 4A.

In the opposite case, with band structures such as illustrated in Fig. 4B, and where the CBM lies higher in energy than *E*_F,M_, and the *E*_F,M_ lies higher in energy than *E*_F,MO_, a flow of electrons from the metal to the MO should occur, but no available empty orbitals at sufficiently low energy exist to accommodate this charge redistribution.

**Fig. 5:**
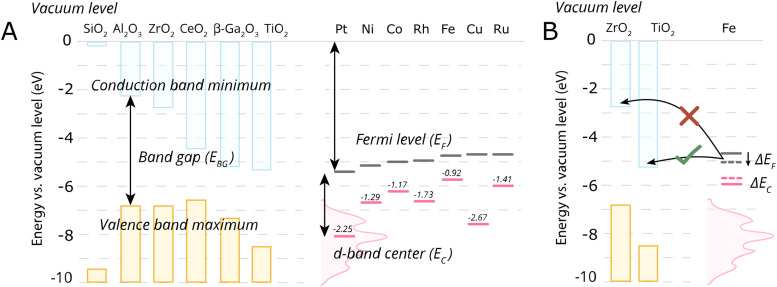
Energetic parameters of importance to describe MSI. (A) For some typical MO supports, the conduction band minima, valence band maxima, and band gap energies of some close packed surfaces (111) are shown, except for Al_2_O_3_ and Ga_2_O_3_ where (100) terminations were used, taken from ref. 72. Fermi levels of the metals, *E*_F_, were taken from ref. 73 and the metal elemental d-band centers *E*_C_ relative to the Fermi levels were taken from ref. 74. (B) A schematic illustration of the electronic interaction of the metal with the MO support based on the conduction band minimum and *E*_F,M_. In the case of Fe on TiO_2_, there will be an electron flow from Fe towards the conduction band of the TiO_2_, while such electron flow does not occur in case of ZrO_2_. The shift of Fe Fermi level also result in the shift of d-band center position which is connected to the activity of AM–MO system.

Despite the simplicity of this model, it was shown that whether or not electronic redistribution from the *E*_F,M_ into the CBM can occur greatly influences the binding strength of the metal to the MO, where a higher degree of redistribution results in stronger interactions. In the case where such an interaction should not occur, such as with SiO_2_, the resulting interactions, namely binding between metal and MO, are expected to be significantly less strong, which is in line with general observations in literature (Fig. 5).^72^

One should then be able to describe the degree of this MSI by electronic redistribution by comparing electronic characteristics between the appropriate metals and metal oxides. To that extent, different parameters can be found describing the physicochemical characteristics of the metal/support interface, including the difference between the metal work function *φ*_M_, which essentially describes the position of *E*_F,M_ (the former can be defined as the minimum energy required to extract an electron from a metal, whereas the latter denotes the highest energy level that an electron occupies at 0 K), and the support's electron affinity (the energy change when an electron is added to a material and which can be simplified as a proxy to describe the position of CBM in this picture, as it is the difference between the vacuum level and the CBM).^72^ The difference in electronegativities of the metal and support have also been used to describe the degree of MSI,^75^ as well as the dipole moment at the interface.^76^

Since such electronic redistribution causes a shift in the position of the *E*_F,M_ upon contact with a support with sufficiently low CBM, it may be expected that also the d-band center, *E*_C_, will shift accordingly as it is typically taken relative to the *E*_F,M_ (Fig. 5B).^74^ The d-band center can be used to approximate the adsorption energies of reactants, which, in turn, can be related to activation energy barriers.^77^ As such, it may be expected that the shift in *E*_C_ resulting from such parameters characterizing the extent of electronic MSI (*e.g.*, *E*_F,M_ – CBM) could be used as a tool to predict the activity of an AM–MO catalyst system.^78^ This premise will be evaluated in Section 7.3.

#### 3.1.2. Surfaces and defects

The discussion so far has been related to pristine bulk, defect-free materials, while even the formation of a surface relative to the bulk creates a disruption in the periodic potential function. For surfaces of semiconductor and insulator materials, rather than discrete energy states corresponding to a valence and conduction band, a continuum or series of discrete levels within the band gap exists.^71^

**Fig. 6:**
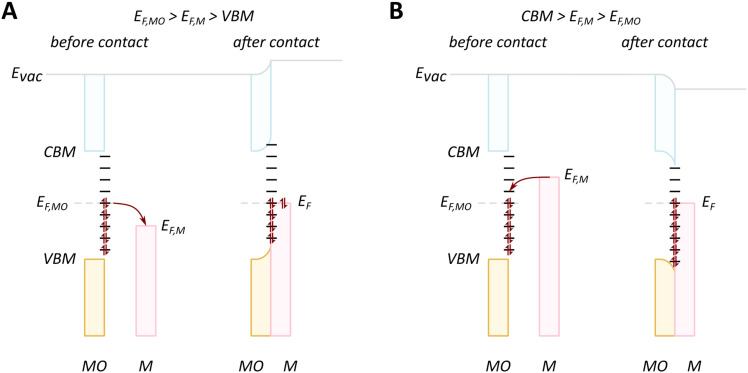
When an MO with electrons in surface states comes into contact with metal, charge redistribution and band bending occurs, the direction of which depends on the relative Fermi level positions. (A) In the case where the Fermi level of the MO (*E*_F,MO_) is higher than *E*_F,M_, the electron flow is from the MO to the metal, and upwards bending of the valence band maximum (VBM) and conduction band minimum (CBM) occurs. (B) The reverse flow is observed in the opposite case. Reproduced from ref. 79.

The presence of the new levels within the band gap makes the forbidden charge redistribution shown in Fig. 4B possible. In this case, when, for example, a metal with a Fermi level lower than that of the MO comes into contact with it, their Fermi levels must be aligned. This causes the energy bands to bend and the surface states to shift.^79^ This phenomenon of band bending is heavily studied in semiconductor physics and excellent review articles can be found describing this phenomenon in detail.^80^ The phenomenon of band bending is a result of the formation of an electric field due to charge redistribution. This leads to the creation of a space charge region, causing the continuous shifting of the energy band edges. This key aspect of band bending is illustrated in Fig. 6A.^80^ The process involves electrons from the MO surface levels moving into the metal, which leads to a slight negative charge on the metal and a slight positive charge on the MO. The reverse flow direction and band bending occur in the opposite case, as shown in Fig. 6B.

In addition to local energy levels within the band gap created by the formation of a surface, crystal lattice defects, such as vacancies, are always present in a solid material at non-zero absolute temperature due to minimization of thermodynamic potential at higher than zero defect concentrations.^81^ Such defects also give rise to additional energy levels between the valence band maximum (VBM) and the CBM. Doping with extrinsic elements, which can either be done intentionally or in the form of an undesired or unknown impurity, can also lead to such localized energy states within the band gap. Furthermore, the particle size and shape of the MO can change the defect density and, in general, the electronic structure. Such phenomena, including bond polarization at the interface, add great complexity to the general description and prediction of the flow of electrons and resulting characteristics in more realistic metal–MO systems, and every case, should be analyzed and characterized separately.^82^ Nevertheless, expected doping effects for the oxides of interest for catalysis can, to a certain extent, be generalized based on their electronic structure.

We can recall that the valence band of metal oxides is typically composed of oxygen 2p levels,^19^ which makes the position of the VBM of the oxides quite low in energy with respect to vacuum level, and a large enthalpic penalty must be paid to create a hole. As a result, it is challenging to make a p-doped oxide, which is characterized by extra hole energy levels close to the VBM. Most MO supports are naturally n-doped semiconductors (for clarity referred to in this manuscript as n-type donor-doped, as X-doped typically refers to doping of a material with X in the field of catalysis), meaning that extra electron energy levels exist close to the CBM.^83^

**Fig. 7:**
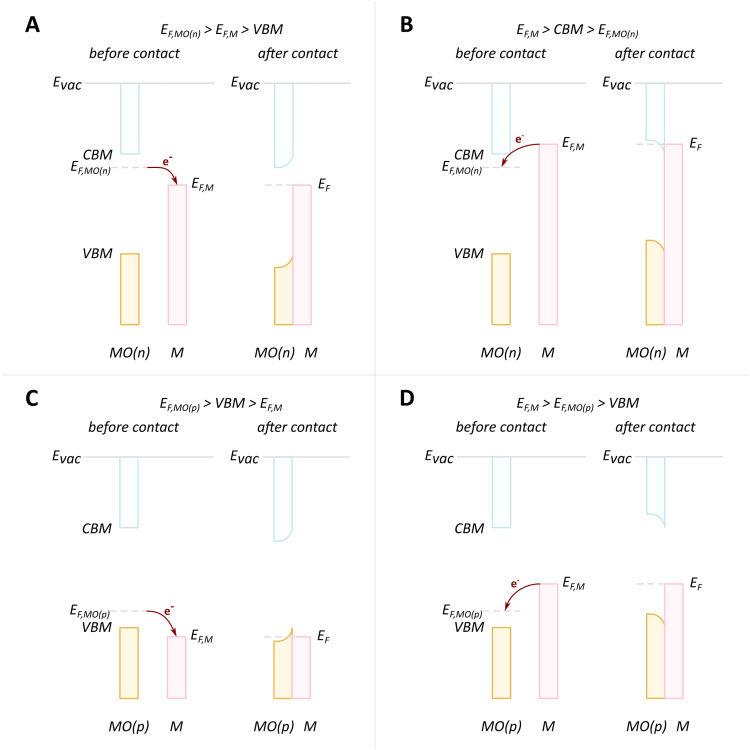
Schematic diagrams of charge redistribution and band bending for different cases of doping types and Fermi level positions. (A) n-type donor-doped MO with Fermi level of MO higher than that of metal; (B) n-type donor-doped MO with Fermi level of MO lower than that of metal; (C) p-doped MO with Fermi level of MO higher than that of metal; (D) p-doped MO with Fermi level of MO lower than that of metal. Adapted with permission from ref. 84. Copyright 2015, Springer Nature.

The evolution of vacancies can significantly affect the expected flow of electrons. For example, in the case of an n-type donor-doped semiconductor, there is electron density near the CBM at standard conditions.^81^ Upon contact of such a semiconductor with a metal with *E*_F_ positioned lower than that of the semiconductor, electron flow will be observed from the semiconductor to the metal. The surface region of the semiconductor, in this case, will be depleted of electrons, and upward band bending will occur, resulting in a potential energy barrier, referred to as Schottky-type junction, which is seen in Fig. 7A. Such charge transfer phenomena were observed in many surface science studies related to understanding c-SMSI phenomena, which were predominantly performed on TiO_2_ as this was the support material c-SMSI was first observed on. These charge transfer phenomena are regarded as a prerequisite for AM encapsulation, or c-SMSI, to occur, which will be further analyzed in the following section. In the opposite case of *E*_F_ position for an n-type donor-doped semiconductor, the electron flow will be in the opposite direction, resulting in what is referred to as an Ohmic-type junction where no potential energy barrier occurs between the metal and MO (Fig. 7B). Although rarer, as was discussed previously, the case of p-doped oxide charge redistribution is also shown in Fig. 7.^84^

The formation of oxygen vacancies is closely related to another phenomenon, namely oxide “reducibility”, which plays a noticeable role in the description of MSI. The higher degree of interaction between metal and support is associated with the higher “reducibility” of the oxide through the more facile formation of oxygen vacancies upon exposure to reducing conditions, acting as n-type dopants and increasing electron density near the CBM of the oxide. In this respect, the “reducibility” of an oxide support could be considered as another descriptor of MSI strength as long as it is itself a well-defined property (see more detailed discussion in Section 3.3).

#### 3.1.3. The effect of metal nanoparticle size on electronic redistribution

The next step in the description of electronic parameters affecting MSI will be the consideration of metal nanoparticle size effects by assessing the expected electronic changes when decreasing the AM nanoparticle size down to the limit of a single atom in SACs.

Ioannides and Verykios considered the metal/n-type donor-doped TiO_2_ system to deduce an expression for size dependence of charge redistribution based on metal–semiconductor contact theory.^62^ The authors first considered the simplified case of Schottky-type charge redistribution shown schematically in Fig. 7A for which the amount of charge transferred through the interface can be calculated numerically using the Poisson equation (eqn (1)) and the expression for charge density for n-type semiconductors (eqn (2)):1
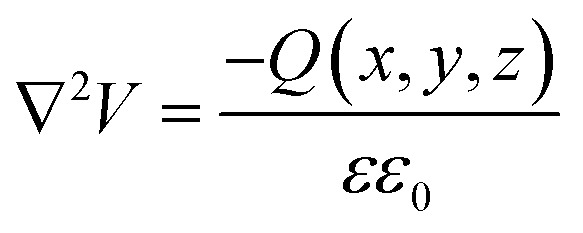
where *V* – potential, *Q* – charge density, *ε* – relative dielectric constant, *ε*_0_ – vacuum permittivity.2
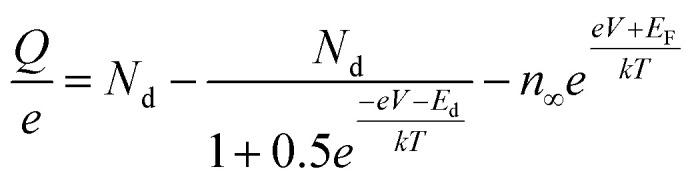
where *N*_d_ – donor concentration, *n*_∞_ – electron concentration in the bulk of the semiconductor, *E*_d_ – donor energy level.

**Fig. 8:**
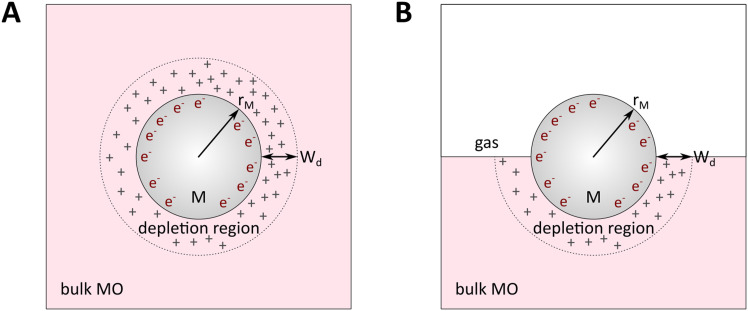
Physical model used to simulate the contact of a metal crystallite with a semiconducting support. (A) Simplified metal particle “immersed” into the bulk MO, and (B) more realistic case with half of the bulk MO “removed”, exposing half of the spherical metal particle to the gas phase. Reproduced with permission from ref. 62. Copyright 1996, Elsevier.

In principle, such a model is only valid for a metal/MO interface extending infinitely on a 2D plane, which, arguably, is adequate for the description of electronic junctions in devices, but is quite far from the case of supported metal NPs and clusters. To account for metal particle size effects, the authors constructed a metal/MO model, consisting of metal particle with radius *r*_M_ embedded in a semiconductor matrix. Due to charge redistribution, there is a charge depletion region of width *W*_d_, as depicted in Fig. 8A. This model allows the use of spherical symmetry to simplify the solution of the Poisson equation. Using the assumption of constant charge density in the depletion region, equal to donor concentration, *N*_d_, the following expressions for contact potential, *V*_0_ (also equal to the differences of work functions, (*Φ*_M_ − *Φ*_SC_)/*e*) (eqn (3)), and number of electrons transferred through the interface, *n*_e_ (eqn (4)), can be obtained:3
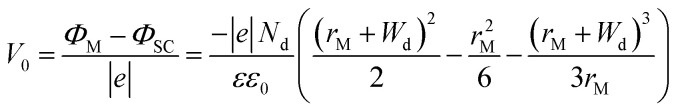
4
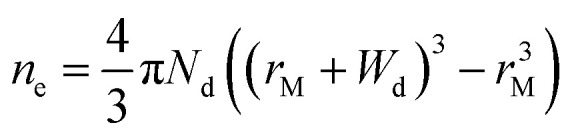


By removing the upper half of the semiconductor, a more realistic model of spherical metal particles deposited on the surface of a semiconductor can be obtained, as depicted in Fig. 8B. The transferred charge is half of the amount predicted by the previous equation.

**Fig. 9:**
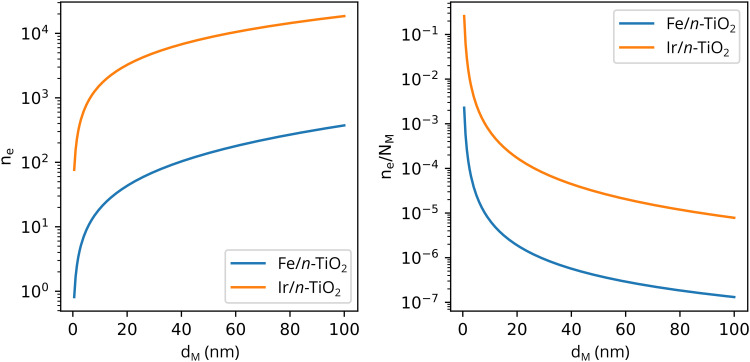
The number of transferred electrons, *n*_e_, and the ratio of *n*_e_ to the number of metal atoms, *N*_M_, as a function of the metal particle diameter, *d*_M_, for Fe/*n*-TiO_2_ and Ir/*n*-TiO_2_ systems, as calculated using eqn (3) and (4). Work functions of metals were taken from ref. 73; work function of n-type donor-doped TiO_2_ was calculated using the equation for the position of the Fermi energy level (−*Φ*_SC_) from ref. 71; for donor concentration, *N*_d_, and effective density of states, typical semiconductor values of 10^15^ cm^−3^ and 10^19^ cm^−3^ were used, respectively.^71^

Solving eqn (3) for certain metal/semiconductor pairs, one can get depletion region width at specific metal particle size. The width can then be used to obtain the amount of charge transferred through the interface at the same particle size using eqn (4). Solving the system of eqn (3) and (4) at different metal particle sizes, one can get the number of transferred electrons and the ratio of electrons per number of metal atoms comprising the nanoparticle as a function of size (Fig. 9). The charge transfer changes from *ca.* 0.2 electrons per metal atom for 2 nm crystallites to less than 0.01 electrons per metal atom for >10 nm crystallites.^62^ As expected, a larger difference between metal and semiconductor work functions leads to an increase in charge transfer.

While a single metal adatom, as opposed to a nanoparticle, does not have a band structure, its electronic structure is still crucially important for understanding its behavior and interaction with the MO substrate. The localized electronic states associated with the metal adatom or small cluster and the surrounding surface states of the MO govern the adsorption properties and the binding energy of metal atoms and clusters onto MOs. The electronic structure of the metal adatom is affected by factors such as the coordination geometry, the availability of nearby bonding or anti-bonding states, and the presence of surface states on the MO surface. The interaction between metal and MO thus determines the overall electronic structure of the system at the adatom/cluster site.

**Fig. 10:**
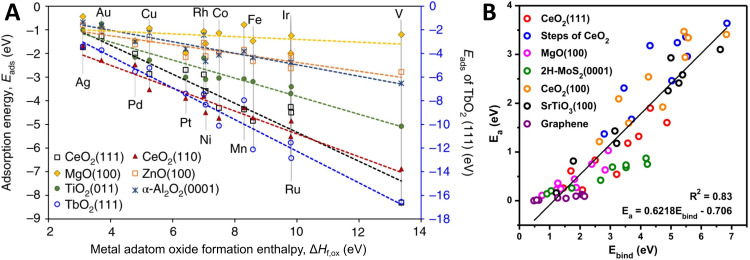
(A) Correlation between metal/support adsorption energies and oxide formation enthalpies of metal adatoms. Different lines show the trends for different MO supports. Adapted with permission from ref. 92. Copyright 2018, Springer Nature. (B) Metal/support interaction determined from the plot of the activation barrier for diffusion (*E*_a_) of metal adatoms against the binding energy (*E*_bind_) of single metal atoms. From ref. 95. Copyright 2020, Springer Nature.

It has been reported that linear scaling relationships exist between metal adsorption energy on a support (interfacial binding strength) and properties of the metal and MO, such as the difference in chemical potentials,^85,86^ metal oxidation enthalpy,^87^ heat of formation of the MO,^88^ “reducibility” of the support (see also the discussion on this term in Section 3.3),^89,90^ and support band gap.^91,92^Fig. 10A shows the linear scaling relationships that were found between the adsorption energy of metal adatoms on different supports and the metal adatom's oxide formation enthalpy. These trends suggest that the interactions of adatoms with surface oxygen atoms largely determine the strength of their interfacial bond with the MO, and the differing slopes of the individual supports show the role of the support's electronic structure in determining the overall metal binding strength. Density functional theory (DFT)-based screening of the adsorption energy of single transition metal atoms over TiO_2_-anatase (101) indicated a clear trend in the metal adsorption behavior, where binding energies of the metal adatoms were found to be strongest for the early transition metal atoms, and were found to correlate well with AM–O dissociation energy. The adsorption energies were found to be weaker for the late transition metal atoms.^93^ Metal adhesion energy on different oxide supports was shown to scale linearly with oxygen adsorption energies on clean fcc (111) metal surfaces, which further supports the importance of AM–O bond formation in MSI.^94^ Both the position of the Fermi level and the density of states near the Fermi level affect the adsorption energy, and this picture for atoms (and clusters) with more localized energy levels corresponds well to the case of bulk interactions, as highlighted in Fig. 5. Furthermore, it is shown in Fig. 10B that the expected rate of metal adatoms surface diffusion can also be estimated by their strength of adsorption onto the MO, as the barrier for diffusion shows a linear correlation to the binding energy of single metal adatoms.

One could argue that the properties highlighted in these linear scaling relations are in fact a measure for the degree of MSI in a system, since the higher binding energy of the metal atom is quite literally a stronger metal–support interaction, as mentioned above. As shown in Fig. 10B, non-reducible or inert supports, such as MgO and graphene, are shown to interact with the metal atoms only weakly. We emphasize that the implication of the reorganization of the electronic levels after AM and MO come in contact is expected to be very important for catalytic activity.

Cheng *et al.* recently reviewed the impact of MSI on Fischer–Tropsch synthesis catalysts. They noted that the adsorption strength of reaction intermediates was indeed tunable by electronic MSI effects such as described above and consequently could be used to control the product distribution.^38^ Theoretical calculations can offer detailed insights into the role of metal–support interactions in heterogeneous catalysis and are particularly useful to deconvolute potential geometric and electronic contributions, such as differing interface sites, and the stability of formed overlayers.^48,96–99^ It can furthermore be concluded that the electronic properties of the MO and metal largely determine the degree and type of expected interaction between the metal and the support.

### 3.2. Geometric effects

**Fig. 11:**
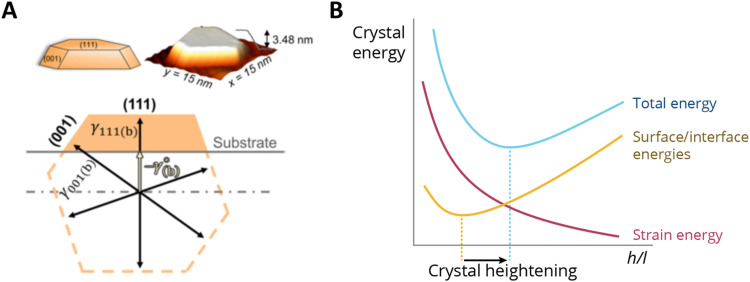
(A) Winterbottom constructions of substrate-supported Pd crystals. A substrate cuts a metal particle in a way that minimizes the total surface and interface energies of the system.^102^ (B) A sketch of the energy components of a metal nanocrystal with a constant volume, as a function of the ratio of height and length (*h*/*l*) when supported on a support with a certain lattice mismatch. The effect of strain makes the *h*/*l* value change and results in crystal heightening. Adapted with permission from ref. 102. Copyright 2021, American Physical Society.

Supported metal atoms, clusters, and particles can undergo geometric reconstruction due to MSI effects, resulting in varying nanoparticle sizes and shapes, and degrees of encapsulation/surface decoration. The shape and size of supported metal NPs relate to the wettability of the supported metal. The equilibrium geometries of non-supported metal NPs in vacuum can be predicted by minimizing the surface Gibbs free energy based on the Wulff construction.^100^ The same principle holds when considering a metal nanoparticle on a support, but one must account for the contribution of the AM/MO interface energy and the surface energy of the support, which are each further affected by the possible effects of, for example, strain and surface tension. In the absence of strain, a supported metal nanoparticle tends to assume a certain height/length (*h*/*l*) ratio, which is largely a function of corresponding facet energies. Such a ratio can be predicted by the Winterbottom construction,^101^ where a substrate cuts a free-standing Wulff crystal (metal particle) to minimize its total surface/interface energies Fig. 11A.^102^ The equilibrium *h*/*l* ratio corresponds to the minimum surface/interface energies. It was shown experimentally that the *h*/*l* ratio increases with the increase of strain induced by crystal lattice mismatch between the metal and MO facets in contact with each other. The thermodynamics behind this phenomenon can be schematically explained as follows: For a metal crystal epitaxially supported on a lattice-mismatched substrate, the total energy comprises the formation enthalpy (which is negative), the surface/interface energies (which are positive), and the strain energy resulting from the lattice mismatch at the interface (which is also positive). Neglecting formation enthalpy, which is fixed regardless of the crystal's shape, the other two energy terms are qualitatively sketched in Fig. 11B. As illustrated in the figure, crystal strain results in crystal heightening.^102^ The effect, however, is less pronounced for particles beyond a critical size for which the interfacial mismatch will be relieved by the formation of dislocations at the interface.

When we consider the possibility of chemical interaction between the AM and MO support, *i.e.*, the formation of alloys, overlayers, or mixed phases, the description of MSI becomes more complex. This involves considering the Δ*G*_f_ of any relevant interactions that may occur between the phases, surfaces, or interfaces of all the atoms present in the AM–MO mix. These are correlated or even largely driven by the electronic properties and structure generalizations discussed above; however, they deserve to be separately discussed as several subtleties arise when explicitly discussing the surface and its energetic properties. This finally leads us to qualitatively describe the range of experimentally observed phenomena, such as the encapsulation of metal NPs by the support and the formation of mixed oxide phases or alloys.

**Fig. 12:**
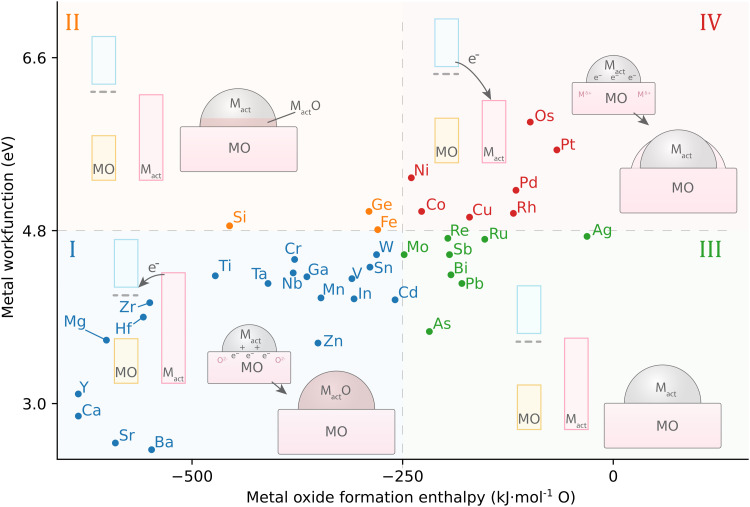
The relationship between AM work function and active MO formation enthalpy leads to four different groups of possible geometric distortions influenced by MSI in systems with n-type donor-doped TiO_2_. Adapted with permission from ref. 70. Copyright 2007, Elsevier. Data for metal work functions is taken from ref. 73; MO formation energy was calculated using HSC 5.11 software for the thermodynamically most stable oxide at 298 K.

Fu and Wagner postulated that the relationship between the work function of the AM, *φ*_M_, and the formation enthalpy of the AM-oxide distinguishes 4 different groups of the behavior of metals on n-type donor-doped TiO_2_.^70^ It was suggested that redox reactions on the TiO_2_ surface are favored if the heat of formation of AM oxides is Δ*H*^0^_f_ < −250 kJ mol^−1^ O (that is, for metals that have a high affinity to oxygen). In this case, a certain degree of bulk oxidation of the AM is expected by means of diffusion of oxygen atoms from TiO_2_. Besides high oxygen affinity, these metals are distinguished by a low work function *φ*_M_, or, consequently, by a higher Fermi level *E*_F_ with respect to TiO_2_ (Fig. 12, group I). After contact between a metal with high *E*_F_ and TiO_2_ is established, charge redistribution occurs, as detailed in the previous section, and the formation of an electric field occurs at the interface, which drives oxygen anion diffusion. The diffusion of oxygen anions from TiO_2_ to the metal proceeds until the chemical potential of oxygen is equal in both phases, which is largely driven by Δ*H*^0^_f_ of the AM oxide. This first of four groups of behavior of metals on TiO_2_ are thus prone to oxidizing, thereby forming oxide phases (such as for alkali metals). Second, metals with *φ*_M_ 3.75–5.0 eV (early transition metals, Fig. 12, group II) see the first to third metal layer at the AM/MO interface oxidized.

On the other hand, for metals with Δ*H*^0^_f_ less negative than −250 kJ mol^−1^ O, *i.e.*, having low affinity to oxygen, chemical bonding with TiO_2_ is not energetically favorable, and two additional behaviors can be distinguished. In general, these metals have *E*_F,M_ lower than that of TiO_2_, which means that the electron flow from TiO_2_ towards the metal is favorable. Charge separation at the interface is then opposite to the previously described cases and is favorable for Ti cation diffusion. Those metals with work functions *φ*_M_ 4.6–5.4 eV (close to the value of TiO_2_, Fig. 12, III group) result in neither oxidation of the AM nor reduction of TiO_2_, and the metal adatoms interact relatively weakly with TiO_2_ and exhibit high surface mobility. Finally, metals with *φ*_M_ > 5.4 eV (Fig. 12, group IV) see no oxidation even at elevated temperatures; only for these metals annealing was observed to lead to encapsulation of the AM nanoparticle, *i.e.*, to the c-SMSI effect.^70^

The delicate balance between the work function of the metal, *φ*_M_, and surface energy, *γ*, of an AM with respect to that of an MO, along with the Δ*G*_f_ of the oxide of the AM, is believed to determine the nature and type of observed c-SMSI effect. The surface free energy *γ* may be defined as the excess energy at the surface of a material compared to the bulk, and the surface free energy is related to, or dependent on, the surface tension. Such energy arises because atoms at the surface have fewer neighboring atoms to interact with, leading to a higher energy state. The *φ*_M_ and surface energy *γ*_M_ are related through the electron density near the surface of the metal and can both be influenced by the surface state of the material and the presence of impurities or adsorbates on the surface. For example, the adsorption of certain species on the metal surface can change the electronic structure, leading to a modification of the *φ*_M_.

Taglauer and Knözinger correlated the encapsulation of AM NPs on MO supports, or c-SMSI, with the surface energy of the oxide supports *γ*_MO_, as they showed that oxides with low surface energy, *e.g.*, TiO_2_ and V_2_O_5_, can more easily participate in encapsulation than those with relatively high surface energy *γ*_MO_ such as SiO_2_ and Al_2_O_3_.^103,104^ The surface energy of the metal *γ*_M_ was then brought into the discussion, suggesting that metals with higher surface energy (*e.g.*, Pt and Pd, but not Au and Cu) favor encapsulation, and it was postulated that minimization of surface energy is, therefore, one of the main driving forces for the reduction of the total free energy of the system.^103–105^ Later, the subsurface non-stoichiometry of the MO and the existence of oxygen vacancies (*i.e.*, doping) were proposed to be a determining factor for encapsulation,^105,106^ as well as the balance between the M–M bonding, and the metal-oxide bonding.^107^ Later, the effect of space charge arising due to electronic redistribution was postulated to be a driving force for species migration, the mechanism of which is described in more detail below.^61^

**Fig. 13:**
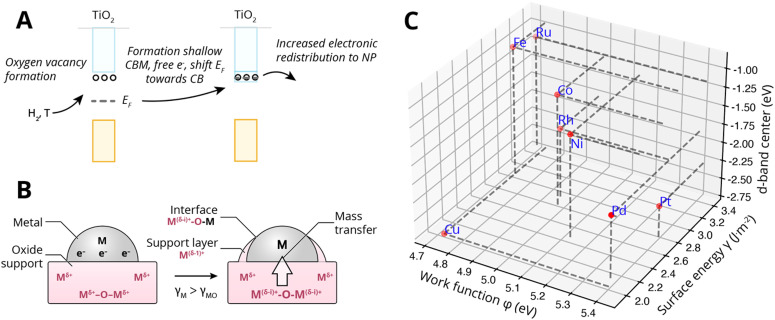
(A) Simplified schematic representation of c-SMSI formation in TiO_2_ following reduction with hydrogen and the formation of oxygen vacancies (n-type doping). This is believed to shift the Fermi level *E*_F_ of the MO upwards, causing increased electronic redistribution towards the metal NP. (B) Schematic illustration of the two prerequisites for encapsulation by mass transfer, first electronic redistribution following the description under (A), and second producing positive space charges near the interface at the MO side and the corresponding electric field that assists the outward diffusion of Ti^*n*+^, and subsequently, the minimization of surface energy for which the prerequisite is that the surface energy of the metal is higher than that of the MO *γ*_M_ > *γ*_MO_. (C) The position of the d-band center relative to Fermi levels of different metals. Surface energies were taken from Mezey and Giber (*T* = 298 K),^108^ work functions from ref. 73, and the metal elemental d-band centers relative to the Fermi levels were taken from ref. 74.

Specifically, encapsulation was believed to occur when TiO_2_ undergoes partial reduction combined with oxygen vacancy formation (see Fig. 13A). The concentration of ionic defects (*e.g.*, interstitial titanium atoms and oxygen vacancies in TiO_2_) is a function of temperature, gas pressure, dopant concentration, and annealing time. If the density of the defect states is sufficiently high, they can develop into a shallow conduction band, thereby producing additional free electrons near the conduction band, shifting the *E*_F_ toward the CB edge of the MO support. Indeed, indications of higher CB electron density were found for TiO_2_ crystals corresponding to strong n-type doping (extra electron energy levels close to the conduction band). The more defective the oxide, the higher its *E*_F_ relative to the *E*_F_ of the metal would become, and the stronger the electron migration to the metal would be. With alignment of the vacuum levels of the two materials AM and MO (*i.e.*, when there is contact), *E*_F_ of the metal will be below the induced shallow CB and thereby the *E*_F_ of the MO will be heightened, which results in equilibration through charge transfer from the occupied donor states in TiO_2_ to the metal. This, then, is believed to form a negatively charged layer on AM and positive space charges in TiO_2_. These space charges then facilitate the outward migration of Ti^*n*+^ atoms due to the electric field induced. Subsequently, if the surface energy of the metal is higher than that of the MO, *γ*_M_ > *γ*_MO_, the formed suboxide species can lower the total energy of the system by redistributing onto the NPs, thereby lowering the surface energy. Later investigations confirmed such electron transfer to enrich the d-orbital electrons of the metal particle. In particular, Fu *et al.*^61^ proved the dependence of the encapsulation process on the electron density in the conduction band of TiO_2_ and on the Pd/TiO_2_ interface.

It is worth repeating that TiO_2_ is by far the most investigated single-crystalline system in MO surface science. Thus, many of the theories above were developed for the case of TiO_2_, which has quite specific properties (*i.e.*, a CBM lower than *φ*_M_ of most metals). The proposed model should be thoroughly evaluated for other metal oxides with different relative positions of CBM and *E*_F_, as well as for both doped and undoped states, particularly when considering recent works that show encapsulation of metal NPs by ‘non-reducible’ supports,^109–112^ or, supports where vacancies are less easily formed. The prerequisite to encapsulation, from what is described above, is the formation of a (strong localized) electric field, which does not necessarily only occur upon charge redistribution due to vacancy formation, but could also occur in materials that accept electrons easily due to low electron affinity, such as is the case for silica, for example. This will be further discussed in Section 3.3.

Fig. 13C shows the work function *φ*_M_*versus* the surface energy *γ*_M_ of several metals that are often used in catalysis. Metals with high surface energies and high work function are expected to be encapsulated by TiO_2_ (*e.g.*, Pt, Pd, Rh, and Ni). A higher *φ*_M_ is believed to increase the charge buildup at the interface, forcing more Ti^*n*+^ migration, and a higher *γ*_M_ means more energy reduction to be gained by encapsulation. Fig. 13C also shows the position of the d-band center *E*_C_ relative to *E*_F_ for the different metals,^74^ which are known to describe the adsorption energies of reaction intermediates as mentioned above. The overall observed activity effects related to metal–support interaction should, therefore, vary within this *φ*, *γ*, *E*_C_ space, and this premise will be further evaluated in Section 7.3 as a function of particle size for CO_2_ hydrogenation and CO oxidation results presented in recent literature.

**Fig. 14:**
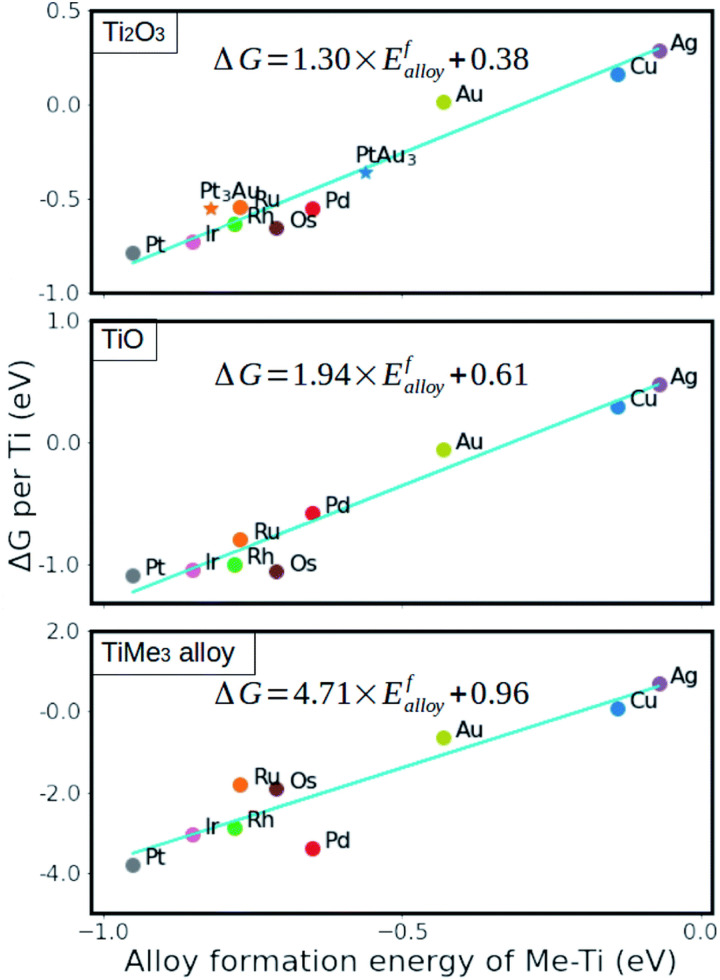
Relative stability of reduced monolayers on metal (111) surfaces as a function of the formation energy of Ti–Me alloys. From ref. 99.

The stability of formed metal suboxide overlayers on metal NPs has been found to be highly dependent on the supported metal environment and treatment, as reported recently by Beck *et al.*^96,113^ The stability and reactivity of metals with respect to the MO overlayers also differ from one another – some are more stable; for example, Cu, Ag, and Au show higher degrees of electron transfer but lower stability because their d-orbitals are already filled. This results in limited charge gain from charge-donating atoms compared to elements with higher d-orbital occupation, and it was shown that the thermodynamic stability of generated overlayers (thin oxide films) on these metals scales linearly with the adsorption energy of the metal oxide cations on the support surface.^98^ The strength of the interaction between metal substrates and reduced oxide monolayers can be determined by the metal alloy formation energy (between the AM and the metal of the oxide support), which is, therefore, a useful parameter to predict the stability of an overlayer.^99^ Using this descriptor, it was shown that anatase layers deposited on Cu, Ru, Pd, Ag, Rh, Os, Ir, Pt, and Au(111) surfaces were more stable than those with the rutile structure (Fig. 14).^99^ The interface energy is particularly crucial if there is a high ratio of interface-to-bulk metal atoms (*R*_int_). The value of the interfacial energy between fully oxidized supports and metal substrates (NPs, clusters, or single atoms) can thus be described by the formation energy of the MO.^98^ However, with alloys comprising metals with significantly different atomic radii, this descriptor might underestimate the effect of the increased strain energy.

**Fig. 15:**
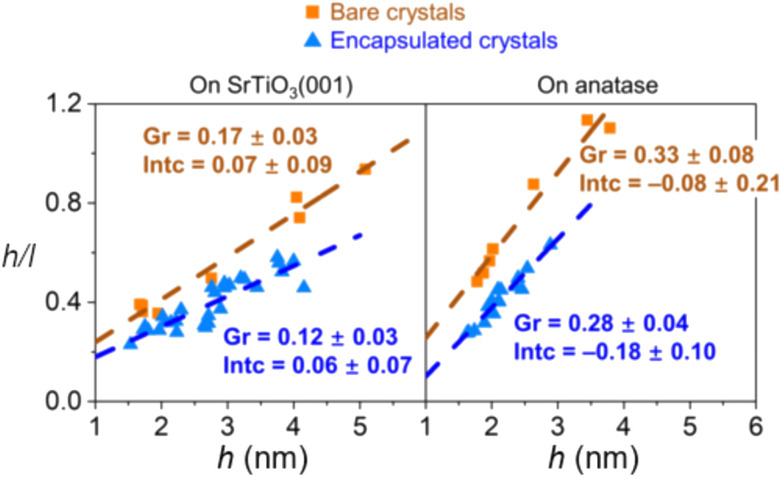
Aspect ratio of Pd crystals plotted against their height for Pd crystals on nanostructured SrTiO_3_ (001) and anatase (001) substrates. Adapted with permission from ref. 102. Copyright 2021, American Physical Society.

The encapsulation process is size-dependent and is believed to be related to the balance between strain, surface tension, and surface energy when considering a nanoparticle with an interfacial mismatch. It was observed that for both bare and encapsulated Pd crystals, the height-to-width ratio increased as a function of crystal height as a mechanism to partially release interfacial mismatch strain with the substrate. However, the authors found that the rate of this increase is lower for encapsulated crystals (Fig. 15), which indicates that with c-SMSI, the interface between the particle and the oxide is altered to form a lower energy interfacial structure, which here also led to less strain in the encapsulated particle. Elastic strain energy scales with crystal volume for a nanoparticle with lattice mismatch, and the authors postulated that c-SMSI, therefore, preferentially occurs on larger crystals.^102^

Pt nanoparticle encapsulation with TiO_*x*_ layers was shown to occur on samples with particle sizes larger than 3 nm only.^114^ The effect was further studied using a model system of Pt NPs and clusters deposited on the rutile (110) plane.^115^ The authors observed overlayer formation in the case of Pt NPs with 1.2 nm in height and 2.2 nm in diameter upon vacuum annealing. In comparison, no encapsulation occurred in the case of Pt clusters with 0.35–0.6 nm in height. The authors related the observed behavior with size dependence of Pt electronic properties and depletion of d-band electron density. Their experimental results, however, also revealed that no TiO_*x*_ suboxide formation was observed in the case of Pt clusters on TiO_2_, which leaves open the question of whether the observed behavior is related to the electronic structure of TiO_2_ (lacking necessary defect electron states required for c-SMSI to occur), or to the change of electronic structure and surface energy of Pt clusters. Du *et al.*^116^ observed a size effect on the c-SMSI in Au/TiO_2_ catalysts and explained it by a thermodynamic equilibrium model, which correlated the degree of encapsulation of Au NPs with their sizes. They found that larger Au particles (∼9 and ∼13 nm) readily undergo c-SMSI compared to smaller ones (∼3 and ∼7 nm). The encapsulation of metal NPs was explained in terms of the surface tension. The authors speculated that the NP surface tension increased with nanoparticle size; larger Au NPs, therefore, showed a stronger tendency to be wetted by TiO_2_.^116^

### 3.3. The concept of “reducible” and “non-reducible” supports

One can think of the classical chemical definition of reducibility as the gain of electrons. But in the context of c-SMSI, where suboxides were first noted to form on “reducible” supports that then encapsulate NPs, the reducibility that comes to mind is perhaps the formation of suboxides or the energy of oxygen vacancy formation. Aside from the context of MSI, the term “reducibility” is also frequently encountered in oxygen-mediated catalytic processes proceeding by means of Mars-van-Krevelen-type mechanisms (*i.e.*, those where lattice oxygen takes part in the catalytic cycle).

Perhaps the most straightforward characteristic describing the degree of oxide “reducibility” we can think of in this respect is the energy of an oxygen vacancy formation. Such characteristics, however, typically cannot easily be obtained experimentally, only theoretically, and problems arise in gaining a generalized understanding as different authors often use different energetic considerations for their calculations and end up with greatly varying numbers for the same oxides.^117–119^

The oxygen vacancy formation process can be described by the removal of a neutral oxygen atom, leaving behind 2 electrons that are accommodated in the cation empty states.^120^ In the case of oxides with predominantly ionic bond character, *vide infra*, cation empty states mainly constitute the conduction band of the oxide while the valence band is filled with electrons from the oxygen 2p level.^19^ Thus, electrons left after neutral oxygen removal should be able to overcome the energy barrier, which is equal to the band gap energy of the metal oxides. The process is energetically unfavorable until the oxide is exposed to reducing conditions which is more probable the smaller the band gap of an oxide is. The band gap then can be considered as a convenient descriptor of oxide “reducibility”, much more so than the Gibbs free energies of formation or reduction reaction Gibbs energy of the MO, as can be seen in Fig. S1 and S2 (ESI†). The nature of binding in metal oxides varies from ionic to covalent, though this distinction between ionic and covalent bonding is somewhat artificial, as many metal oxides exhibit a continuum of bonding types, perhaps with one character dominating over the other. Generally, metal oxides formed by alkali metals (Group 1) and alkaline earth metals (Group 2) tend to be more ionic. These metals have low electronegativity, and when they react, they donate electrons to oxygen, forming metal cations and oxide anions. Transition metal oxides, especially those in intermediate oxidation states, can have an intermediate character with a combination of ionic and covalent bonding. Metal oxides formed by non-metals and metalloids (*e.g.*, Group 14 elements like silicon) tend to have higher electronegativity, and the sharing of electrons between the metal and oxygen leads to a more covalent nature of bonding. In the case of oxide compounds with more pronounced covalent bonding character the overlap between oxygen and metal atomic energy levels results in a mixed contribution of oxygen and metal orbitals towards the density of states of the valence and conduction bands. It is thus harder to rationalize the influence of the band gap value on the low “reducibility” of, *e.g.*, SiO_2_ or Al_2_O_3_, which have stronger covalent bonding character.^121^ To characterize the majority of what is commonly referred to as “reducible” supports, we may best assert that the top of the valence band is separated relatively far from the vacuum, and the bottom of the conduction band is also quite far from it (implying a small band gap), which means a relatively high affinity to electronic perturbations. For “non-reducible” supports, on the other hand, relative chemical inertness is marked by a large band gap, a VBM far away from the vacuum level, and a CBM close to it, as depicted in Fig. 5.

While there is clearly a correlation between the heuristic description of “reducibility” and the proxy of band gap such as described above, there is also a clear discrepancy between this description and phenomena reported in recent works where “non-reducible” supports show very similar behaviors to those classically only observed for “reducible” supports (*i.e.*, encapsulation in reductive environments, although at higher reduction temperatures or in presence of an electron beam).^109–112^ The “ease of removal of an oxygen atom” is certainly not the only, or even most common way to describe reduction. The chemical definition, the process of gaining electrons (or decreasing oxidation state), rather, would be more suitable to describe the entirety of the observed effects.

The direction and degree of loss or gain of electrons upon intimate contact are perhaps better described by the theories surrounding contact-electrification. In fact, this mechanism is said to be one of the oldest unresolved, universal problems in physics.^122^ The ability of an MO to gain or lose electrons during contact with an AM in this context is influenced by various factors, and the band gap is just one of them, among surface chemistry, electron affinity, and the material's ability to trap and hold charges. The chemical nature of the surface atoms and the presence of specific functional groups can significantly influence charge transfer, as already discussed in subsection 3.1. Particularly in the situation where we compare bulk electronic redistribution to shallow electronic redistribution, the electron affinity can have a stronger relative influence on the observed effects, as the accumulation of charge at the surface of a material can create local electric fields. The local permittivity near the surface can be affected by these surface charges and states, altering the material's overall dielectric properties. At the interface between two materials, differences in electron affinity (or *E*_F,M_ and CBM) can lead to charge transfer and the formation of dipoles, which affect the local electric field. It is conceivable that the existence of a local electric field would influence, for example, the diffusion of a Si cation in a manner similar to the diffusion of a Ti cation. The gradient of the electric field is expected to be larger for a material with low permittivity, for example, in SiO_2_*versus* in TiO_2_, so locally the cations could be attracted even stronger. This, however, is all speculation and needs to be researched both practically and theoretically in much more detail.

## 4. Critical assessment of MSI subclassifications

**Fig. 16:**
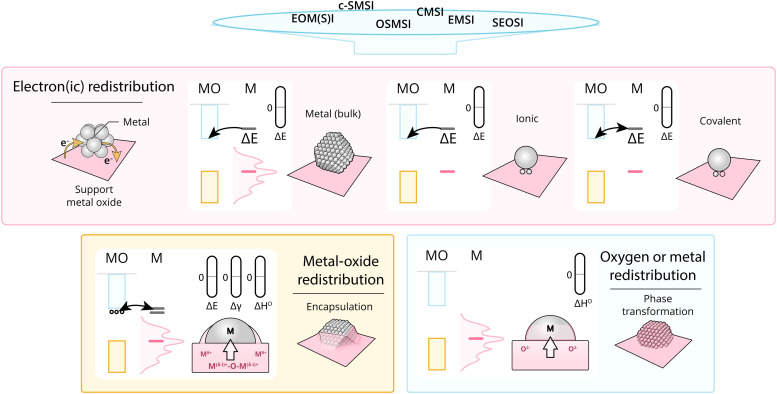
Schematic overview dividing MSI classes into fundamentally different physical phenomena. These phenomena start with electronic redistribution (Δ*E*), where (at least) three fundamentally different types should be distinguished; nanoparticle (*i.e.*, band structure redistribution), ionic, and covalent bonds between single atoms or clusters and the support. Following this electronic redistribution, depending on additional parameters, enthalpy of oxide formation, and surface energy, the oxygen or MO can redistribute leading to phase transformation and encapsulation.

Given what was discussed previously, it is useful to assess the existing terms addressing the main fundamental underlying characteristics of MSI (Fig. 16).

**Fig. 17:**
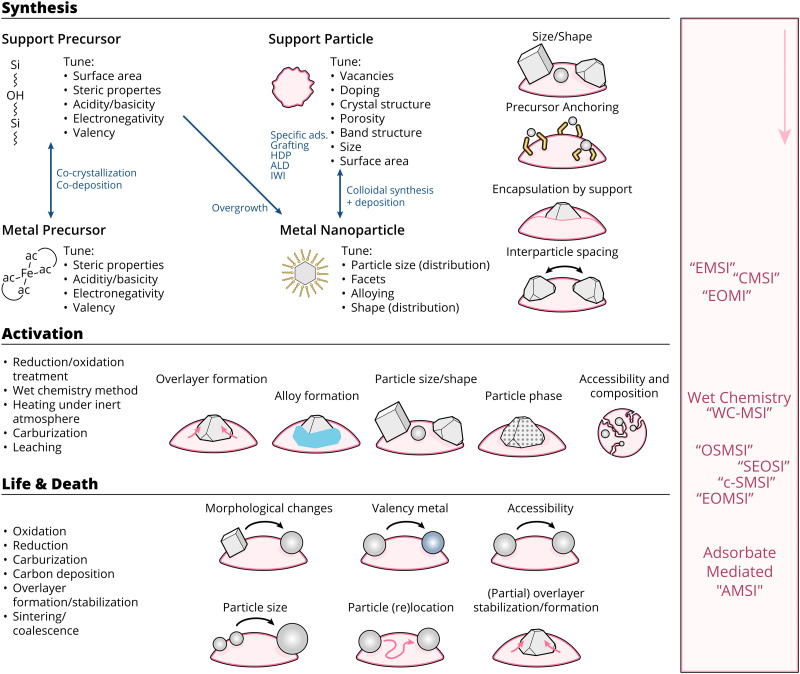
Schematic overview of the means through which MSI can be tuned, as well as the resulting physicochemical properties affected by these means throughout the lifetime of a catalyst (*i.e.*, during synthesis, activation, reaction, deactivation). The right side of the figure shows typically used abbreviations in literature, and the point in the lifetime of a catalyst when they are at play.

As discussed in Section 3.1, the simplest type of interaction involves the redistribution of electron density between the AM and MO support. It occurs once contact between them is established. It is broadly termed EMSI in the literature and influences supported particles size^72^ and shape.^47^ The strength of this type of interaction is determined by the differences in electronic structures of AM and MO, which can be expressed by means of different quantitative descriptors. Although charge redistribution, described in Section 3.1, is also an essential part of other types of interactions, as was demonstrated in Section 3.2, it directly influences the catalytic activity of the resulting material, and its impact should be assessed directly when there is no formation of new phases or encapsulation (*e.g.*, geometric restructuring) at the interface during catalyst activation and operation. It is thus a prerequisite for using quantitative descriptors of electronic structure differences in relations between catalyst activity and composition for this type of MSI. It was shown, for example, that this type of interaction occurs in Cu/TiO_2_ systems,^123^ in which charge transfer from low work function Cu NPs to the TiO_2_ support was postulated. Neither new phase formation nor encapsulation was observed upon heat treatments. Positively charged Pd particles were observed in the Pd/sodium fluorohectorite clay system, which were stable during CO oxidation tests at temperatures up to 250 °C.^124^

EMSI can also be found in other combinations of active NPs/support materials classes. It was encountered in systems consisting of active oxide NPs deposited on oxide supports (SEOSI). For example, in In_2_O_3_ NPs deposited on monoclinic Li-doped ZrO_2_ support, electron transfer from ZrO_2_ towards In_2_O_3_ was observed under CO_2_ hydrogenation conditions.^28^ Oxide-supported oxide catalysts contain metal–oxygen–metal (M–O–M) structural units, and (not unlike for oxide-supported metal catalysts) alteration of interfacial M–O–M can be an important tool to adjust binding strength and local coordination, providing a large variety of different chemical environments as possible active sites. Electron transfer from ZnO with a higher Fermi level than CuO was postulated in the CuO/ZnO system with the formation of an electron-rich interface.^125^

The same type of electronic interaction was also studied using model inverse catalyst systems with MO support NPs deposited on AM monocrystals (EOMI/EOMSI).^24,27,28,126,127^ The inverse catalyst systems can be used to generate specific interfaces or exclude certain interfaces, thus simplifying the investigation of the interaction between the metal and the oxide components.^128^ Charge transfer between Cu(111) and CeO_*x*_ was postulated to occur *via* reduction of Ce^4+^ to Ce^3+^ in experimental^129^ and theoretical^130^ works. The strong chemical bonds were formed *via* hybridization between O 2p and Cu 3d orbitals, as was proposed from DFT calculations. A theoretical study of CeO_2_ supported on (111) planes of different metals revealed that metal work function and structural match/mismatch are key parameters that determine interaction strength, with charge transfer being higher for metals with lower work functions.^131^

EMSI of different strengths can be obtained by redistributing the electron density to modulate chemical interactions in supported metal particles. Such electron redistribution depends on particle size: single atoms, clusters, or NPs.^40^ Charge redistribution is perceived more by NPs smaller in size with the limit of single atoms distributed over the surface of the support. This case was specifically distinguished in the literature by the term CMSI.^25,34,132^ Although observed phenomena in this case also involve charge redistribution and can be quantified by differences in electronic structures of “supported” atoms and the oxide support, they indeed have some specifics that set CMSI slightly apart from EMSI due to its localized character, and also typically a stronger relative contribution to the electronic state and local geometric environment of the AM. When deposited on supports, it has been noted that even small perturbations resulting from electron transfer between metal atoms or clusters and the support, can generate localized electric fields by exposed cations or anions of the support, which, in turn can exert a large electronic influence on catalytic activity.^40^ The observed charge transfer between single atoms and supports is believed to be a result of the bonding of metal atoms with anchoring sites on the supports.^133–135^ It is no longer possible to determine “encapsulation” and “new AM–MO phase formation” for these systems. In order to be able to compare them consistently with EMSI in NPs/MO systems using corresponding quantified differences in electronic structure, one should ensure there is no formation of AM NPs due to surface AM atom diffusion, or their dissolution in the crystal lattice of the support. It is not surprising that high AM–MO bond strength was frequently connected to increased sintering stability of the resulting material. CMSI was shown to occur in Au_1_/FeO_*x*_ systems, where positively charged species were anchored on an iron oxide support.^25^ Examination of aberration-corrected high-angle annular dark-field (HAADF) images of the catalyst after a 100-hour CO oxidation experiment performed at 200 °C did not reveal the formation of Au NPs, and the formation of a strong chemical bond between the AM and the support was postulated. The formation of the strong bond between Pt atoms and the surface of TiO_2_ support was observed by Han *et al.*^136^ Although the effect was prominent after the high-temperature reduction, and was represented by CO adsorption suppression similar to c-SMSI, no “encapsulation” of Pt single atoms was shown to occur. The observed effect, thus, can be attributed to the formation of strong bonds after partial reduction of the TiO_2_ support, and is more likely to be described by the CMSI term. DFT calculations revealed significant charge redistribution and covalent bonding formation between Ir single atoms and the WO_3_ support, which affected the associated energy barrier of CO_2_ cycloaddition to epoxides reaction.^137^ A similar covalent bonding nature was found for a Pt_1_/CoFe_2_O_4_ SAC catalyst with a Pt–O–Fe bonding at the interface.^132^

EMSI is also at play in metal clusters supported on oxide supports. Clusters, as opposed to bulk NPs, have more discrete electronic states along with an increase of Fermi energy level.^138,139^ Attaching to an MO with a different Fermi energy level from the metal species, the results are interfacial charge transfer and electronic reorganization, and increasing the particle size above a given number of atoms suppresses further charge transfer. Strong charge transfer between Pt clusters and an Al_2_O_3_ support was reported, resulting in charge-deficient states of the metal clusters compared with those of metallic NPs.^140^

The certain ratio between AM oxide formation enthalpy, AM work function, and AM/MO surface energies allows for geometric rearrangement to occur. The most well-known case of this type of phenomenon – the encapsulation of metal NPs with oxide support material – was first described as SMSI in literature and is often termed c-SMSI nowadays. The phenomenon occurs between Group VIII noble metals and transition MO supports after exposure of the catalysts to high-temperature reduction and is recognized most often by suppression of CO or H_2_ chemisorption.^21^ A study of different transition metal oxides revealed a correlation between the lowest temperature of reduction for chemisorption suppression to occur and oxide reducibility.^54^ Unequivocal evidence of overlayer formation under reducing conditions and reversible metal particle surface recovery under oxidative conditions was provided by means of *in situ* electron microscopic techniques, and this indicator warrants this type of MSI to indeed be discussed separately.^29,141,142^

The formation of overlayers, as discussed for c-SMSI, is typically said to occur under reductive conditions; however, recently, the effect was also encountered in oxidizing environments (where it is commonly termed OSMSI).^39^ Also in this case, the balance between AM oxide formation enthalpy, AM work function, and surface energies is what results in charge redistribution with subsequent AM nanoparticle encapsulation. Although this phenomenon is discussed separately, the fundamental physical phenomena underlying it closely resemble or are even identical to c-SMSI. Dedicated review articles on OSMSI can be found in the literature.^26–28^ The phenomenon was first observed in Au/ZnO^26^ and confirmed to occur in different systems, comprising gold deposited on Al_2_O_3_^111^ and TiO_2_ after surface modification with melamine.^68^ It is also worthwhile to note that in most cases of OSMSI treatment conditions were not typical of c-SMSI and higher temperature, or additional surface modifications were required.

It is noteworthy that apart from encapsulation, other geometric rearrangements can occur because of MSI, including the formation of mixed oxide phases and alloys.^143^ A homogenous catalyst phase with distinct catalytic properties, such as solid solutions or spinels, can result when the interactions between cations of the MO support and the AM constituent are very strong.^144^ However, despite such cases representing distinctly different characteristics from other cases in observed geometric changes, these types of MSI are not specifically afforded any subclassification. Rather, they are typically designated as general SMSI throughout the literature. One may argue that if c-SMSI deserves a subclassification, such cases could also be afforded their own. Praliaud *et al.* studied a Ni/SiO_2_ system and observed c-SMSI-like behavior with suppression of H_2_ chemisorption after high-temperature reduction of the catalyst, although the reduction temperature required to achieve this was higher than that reported for Pt/TiO_2_ materials.^145^ The effect, however, was suggested to occur by Ni–Si alloy formation, and not encapsulation. This was also shown to occur in Rh/SiO_2_,^146^ Pt/SiO_2_,^147^ Pd/SiO_2_,^148^ Pt/Al_2_O_3_,^149^ and Cu/ZnO^150^ systems.

Under appropriate conditions, oxide supports can interact with metal particles and form alloys due to the removal of oxygen during the reduction of the metal/support interface.^21,51,96,99,151–153^ This behavior was recognized as early as the discovery of the c-SMSI phenomenon. Tauster *et al.*^18^ observed the formation of Pt–Ti alloys in the form of Pt_8_Ti and Pt_3_Ti, and others reported surface Pt–Ti alloys formed at 673 K in the presence of SMSI.^154,155^ The formation of alloys of Ti with Pt and Ir (TiPt_3_ and TiIr_3_) resulted from a strong interaction between the metal species.^156^ Beck *et al.*^96^ demonstrated that migration of reduced TiO_*x*_ onto the Pt particle surface also formed an alloy of Pt and Ti at the Pt–TiO_*x*_ interface after a high-temperature treatment in H_2_ atmosphere in a competitive mechanism with overlayer formation. The incorporation of Ti into the Pt crystal structure after a high-temperature reduction caused lattice contraction. Indeed, the formation of Pt–Ti alloys in the presence of SMSI in Pt/TiO_2_ is both experimentally and theoretically feasible, but experimentally, it needs high temperatures, *i.e.*, typically above 873 K, which is higher than where c-SMSI effects typically take place (at temperatures of 673–773 K). Unique interfacial structures, such as exposed reactive facets, morphology, as well as size control, may lower the temperature requirements.

## 5. Generation and control of MSI

MSI effects play a central role in heterogeneous catalysis and provide a means to tune the activity, selectivity, and stability of the materials. Thus, it is essential to be able to control the extent of MSI by altering the synthesis parameters of the catalyst. While it may be possible to control the extent of MSI during synthesis, the situation could be quite different under reaction conditions. In this section, we provide an overview of general principles one can use to control the extent of MSI during synthesis and activation steps.

### 5.1. Control of electronic metal–support interaction

As we have seen in the previous sections, charge redistribution occurs as soon as a metal and metal oxide support are brought in contact. The means of controlling the extent of charge redistribution are based solely on tuning the electronic structure of the metal and support materials. This can be achieved *via* defect engineering, doping, alloying, and other approaches mainly aimed at changing the metal Fermi level position or the band structure of the support oxide.

#### 5.1.1. Support material composition and degree of reduction

The EMSI strength depends on the differences in electronic structures of the support and the deposited AM. Thus, changing the support material composition is a straightforward way to change EMSI. However, as we will see later, it is essential to consider possible defects in the MO crystal structure, which can drastically change the electron structure of the support, introduce new energy levels near the CBM or VBM, as well as change the direction and extent of charge redistribution.

Materials commonly studied and utilized as supports in heterogeneous catalysis include metal oxides (reducible, *e.g.*, CeO_2_ and TiO_2_, and “non-reducible”, *e.g.*, Al_2_O_3_ and SiO_2_) and their composites, *e.g.*, CeO_2_–ZrO_2_ and TiO_2_–ZrO_2_ as mentioned throughout the text, but also include carbon materials, nitrides, zeolites, and metal–organic framework materials.^36,155,157–160^ It is clear that the choice of support is critical to the functioning of a heterogeneous catalyst system, and several detailed discussions on support effects exist in the recently published literature.^40,161,162^

As an example of altered surface properties of catalysts by choice of support composition, Chen *et al.* aimed to induce a reverse effect to that of c-SMSI in terms of H_2_ and CO adsorption suppression by changing the charge of supported Cu NPs. Selection of the appropriate relative levels of the work functions of copper and oxide support resulted in positively charged copper in Cu/MO systems with the MO work function higher than that of bulk copper.^163^

#### 5.1.2. Oxide lattice plane termination

Control over different lattice plane terminations can be achieved using the synthesis of oxides with well-defined morphologies. Ha *et al.* synthesized Au catalysts supported on CeO_2_ cubes and octahedra, having (100) and (111) surface planes, respectively.^164^ They found that, while Au NPs were positively charged in both cases, the Au/CeO_2_ (100) catalyst showed a weaker Au–Au bond due to stronger MSI, corresponding to stronger charge separation. Because of the correspondingly different energetics of different lattice planes, it is nearly impossible to synthesize catalysts that differ only by support morphology. If one were to commence synthesis with supports of varying morphologies, they would end up with catalysts containing metal particles of varying dispersion, shapes, and size (distributions).^165^ Different oxide support morphologies can have significantly varying concentrations of lattice oxygen vacancies. It has been shown, for example, that different CeO_2_ planes exposed to the surface of particles have different energies of oxygen vacancy formation, which in turn lead to the preferential formation of vacancies in rod-shaped CeO_2_ particles in comparison to cubic ones.^166^ It is thus highly difficult to explicate the effects of lattice terminations separately from other effects and, consequently, to (exclusively) use support morphology as a means of MSI control.

#### 5.1.3. Oxygen vacancies and degree of reduction

The electronic structure of oxides can be changed by the introduction of oxygen vacancies into the MO lattice. This can have significant effects on the catalytic activity, as was recently shown by Belgamwar and coworkers, who stabilized Cu clusters on a TiO_2_-coated fibrous nano-silica support to form a highly active catalyst for the conversion of CO_2_ to CO.^167^ In another study, it was shown that the work function of MoO_3_ was lowered by 0.5 eV through the introduction of vacancies. A shallow band near the Fermi level appeared, making the oxide an n-type donor-doped semiconductor.^168^ Such a change in electronic structure can completely change the degree of charge redistribution and its direction. In a Ru/ZrO_2_ system without defects, the charge transfer proceeded from the Ru nanoparticle towards the oxide support,^169,170^ while the introduction of only a single oxygen vacancy at the interface resulted in 0.67*e*^−^ charge transfer from the support to the nanoparticle, as demonstrated by DFT calculations.^169^ This showcases the strength of the effect of oxygen vacancies on EMSI and suggests that the general rules used to predict charge transfer in supported metal oxides have somewhat limited prediction power. Nevertheless, for a certain metal/support combination, oxygen vacancies tend to favor electron transfer from the support to the AM phase due to a decrease in work function and the introduction of new sub bands close to the CBM of the oxide.

Different concentrations of defects in TiO_2_ supports can be achieved by the choice of catalyst preparation method. In the work of Long *et al.*, Ni_3_Fe clusters were anchored on TiO_2_ by means of hydrothermal, co-precipitation, and impregnation methods.^171^ Oxygen vacancy concentrations decreased in the order co-precipitation > impregnation > hydrothermal synthesis. A corresponding increase in charge transfer from TiO_2_ to Ni_3_Fe was observed for samples with higher O_v_ concentrations. A similar trend was also observed for the Rh/TiO_2_ system, for which the introduction of oxygen vacancies resulted in the decrease of the MO work function and easier transfer of electrons to the AM, leading to negatively charged Rh particles.^172^ The energy of oxygen vacancy formation can also be controlled by the size of support particles, as was shown for Co/ceria–zirconia.^173^ It was pointed out that the process was facilitated by the presence of Co-based phases by means of spillover of oxygen at lower temperatures.

One of the methods that proved to be useful for this purpose is high-temperature reduction of mixed oxides.^174^ Naeem *et al.* reported the reductive exsolution of metallic elements from solid solutions at a high temperature.^175^ The applied conditions played an important role in tuning the geometric and electronic properties. They showed that Ru exsolution from fluorite-type Sm_2_Ru_*x*_Ce_2−*x*_O_7_ at 700 °C yielded dispersed supported metallic NPs of *ca.* 1 nm in diameter fixed in the host oxide surface, leading to changes in electronic state. *In situ* X-ray absorption spectroscopy at the Ru K-edge showed that with increasing temperature, the exsolution of Ru from Sm_2_Ru_0.2_Ce_1.8_O_7_ in H_2_ atmosphere proceeded *via* an intermediate Ru^*δ*+^ state, *i.e.*, Ru^4+^ → Ru^*δ*+^ → Ru^0^. XPS showed that Ce^4+^ ions reduced to Ce^3+^, together with an electron transfer from the reduced host oxide to the exsolved Ru clusters, creating Ru^*δ*−^ states.

The surface of a support oxide can be modified in a way that changes the local electron density and induces deviations from bulk electron structures. Chen *et al.* reported an increase of MSI strength between Ru and non-reducible Al_2_O_3_ upon high-temperature reduction in H_2_. The effect was attributed to modification of the alumina surface with highly basic, *i.e.*, electron donating hydroxylated alumina sites.^176^

Support “reducibility” was considered as a useful descriptor to predict the extent of charge redistribution. It was shown, for example, in theoretical work by G. R. Jennes and J. R. Schmidt that relatively inert SiO_2_ does not induce substantial charge transfer through the metal/support interface, resulting in a 0.25|e^−^| charge on the Rh nanoparticles, while in the case of TiO_2_, the amount of charge accumulated on the nanoparticles was tenfold.^177^ Experimentally, it was shown that the change in the unoccupied d-electron density of Pt deposited on Y_2_O_3_ and ZrO_2_ was larger upon reduction than in the cases of TiO_2_ and Nb_2_O_5_ supports, traditionally considered reducible oxides. The effect was attributed to stronger interaction in the latter case. We note that it is possible, however, that an oxide overlayer was also formed in Pt/TiO_2_ and Pt/Nb_2_O_5_ systems, aside from charge redistribution through the AM/MO interface.

#### 5.1.4. Support oxide doping and active metal alloying

Doping is arguably the most effective way to tune the electronic structure of a given oxide support and achieve a certain desired EMSI strength. One should be aware of a well-known “doping asymmetry” problem in oxides, however, when designing a doping strategy. It is known, for example, that ZnO is readily dopable by electron donors leading to increased n-type conductivity, and is hardly dopable by holes.^178^ The main reason for this behavior stems from the fact that the VBM of such oxides is comprised mainly of highly localized 2p orbitals of oxygen with low energy with respect to the vacuum level. To make a p-doped oxide, one has to find a material in which metal cations introduce local energy states near the VBM of the MO so that the VBM becomes more delocalized, which is challenging as most such dopants will do so near the CBM.^179^ The most well-known examples of p-type oxides are NiO and Cu_2_O,^180^ though these materials have little usage as supports in catalysis.

Control of charge transfer between the AM and MO, based on metal/semiconductor interface theories is a topic of active discussion in the literature.^181^ Although the significance of the effect was criticized based on the fact that metals have a high concentration of free electrons and only minimal perturbations can likely be induced by such charge transfer, Akubuiro and Verykios pointed out that in the case of small NPs of active metals, the volumetric concentration of electrons transferred to the metal can be substantial.^182^ In their work, the authors controlled charge transfer by altervalent TiO_2_ support doping. No charge transfer was observed in the case of Pt on titania doped with cations of lower or equal valence (K^+^, Mg^2+^, Ge^4+^). In comparison, metal NPs became negatively charged when TiO_2_ was doped with cations of higher valence (Ta^5+^, Sb^5+^, W^6+^). Other cations of higher valence (V^5+^, Nb^5+^, Cr^6+^) showed the same trend.^183^ The effect was also demonstrated for Rh deposited on W^6+^-doped TiO_2_.^184^ In recent work by Chen *et al.*, the authors confirmed the presence of negative charge on Pd NPs after doping a TiO_2_ support with Mo^6+^, while they also pointed out that doping affected the dispersion of supported NPs and the catalysts should be characterized extensively to address purely electronic effects.^185^ Besides altervalent cation doping, oxygen substitution was also considered as a way to tune EMSI. Chen *et al.* compared Pd supported on n-type doped and undoped TiO_2_, and showed that electron-deficient Pd NPs formed in the case of n-doping in comparison to undoped TiO_2_ support.^186^

Altervalent cation doping is usually done by high-temperature annealing of oxide support with the oxides of the dopant metal.^182^ In the case of oxygen substitution with other anions, the method depends on the anion of choice. For example, n-doping can be achieved by high-temperature treatment of oxide in NH_3_, a process known as ammonolysis.^181^

“Doping” can also be done at the site of the AM, referred to as alloying, in which case its work function changes and, as a consequence, so can the quantity and/or direction of charge redistribution. The drawback of this approach is that the electron density distribution of the metal particle changes as well, which directly affects the strength of interaction with adsorbates. It is thus not trivial to distinguish between MSI and non-MSI related effects in catalysis induced by “doping” of the AM. The effects of such doping are more often described in the literature as electronic redistribution between elements of the active phase alloys. For example, a shift of electron density from Zn to Pt was observed upon ZnPt alloy formation during reductive calcination of Pt/ZnO catalysts.^187^ Transfer of electrons from Ti_3_C_2_ with a surface layer of TiO_2_ to the AM was observed in the case of a PdAg alloy. Based on the XPS analysis of the authors, however, the addition of Ag to Pd had negligible effect on the transfer.^188^

#### 5.1.5. Control of CMSI

AM nanoparticle size decrease results in a more pronounced effect of charge redistribution which, in the limiting case of SACs, reaches its maximum. The main trade-off of SACs is their limited stability due to the relatively high degree of under-coordination of the metal atoms (which arguably is also the main reason for elevated activities). This can be modulated and enhanced by MSI by utilizing supports able to form metallic or covalent bonds to the dispersed metal atoms such as metal borides, semiconductors, or oxide films,^189^ or by constructing a bifunctional support, where one constituent has a strong interaction, and the other has a weak interaction with the metal particles, such as CeO_2_@ZrO_2_ with CeO_2_ and ZrO_2_, respectively, binding strongly and weakly with Au.^189^

Due to the high ratio of surface AM atoms in SACs, these systems respond noticeably to any change in the MO surface chemical composition. It has been reported recently that catalysts can be treated in solvents (*e.g.*, water) to vary the extent of catalyst–support interaction by means of the introduction of terminal functional groups.^132^ Simple water treatment of a catalyst can weaken a strong interaction by H^+^ bonding produced by water dissociation onto the metal/support interface, reducing the interfacial charge transfer. Thus, this strategy can alter the electronic structure of supported metal atoms. Tuning MSI properties in this way was achieved by soaking Pt SACs in water, which altered the strong CMSI to a weaker interaction.^132^ The water treatment altered only the local environment of Pt atoms but had no effect on the dispersion and stability of Pt atoms.

Activation treatments can also be used to tweak the extent of CMSI. For example, Zhang *et al.*^190^ tuned the degree of MSI in atomically dispersed Pt catalysts supported on CeO_2_ by applying different activation conditions. Characterization of the catalysts showed that reducing Pt–CeO_2_ at high temperatures by H_2_ led to weak MSI with increased Pt electron density. It was thus concluded that activation of catalysts with strong MSI at high temperatures gradually decreases the degree of MSI in SAC systems.^190^

### 5.2. Generation and control of c-SMSI

Substantial restructuring occurs in many supported catalysts during activation, as well as catalysis due to chemical interactions between the AM and the support, and the diffusion of metal and oxygen atoms. As we saw in Section 3.2, the relationship between the work functions of the AM and MO, the enthalpy of formation of the AM-based oxide, and the AM and MO surface energies can be used to predict (in first approximation) the mechanism of MSI. Similar to EMSI, changing such parameters is a way to control the extent of c-SMSI.

#### 5.2.1. Oxygen vacancies, degree of reduction, and doping

The relationship between the work functions of a metal and metal oxide support can be changed by means of oxygen vacancy creation, for which the most straightforward method is treatment at elevated temperatures in reducing atmosphere. Formation of oxygen vacancies results in the appearance of a filled shallow band inside the band gap region, close to the CBM level of the oxide, as well as an increase of the Fermi level of the oxide. In addition, oxygen vacancies impart the oxide surface with high local electron density, which can be transferred to metal constituents at nearby interface sites, thus influencing the metal–support interactions. Reduction of reducible metal oxides at temperatures ranging from 300 to 700 °C can induce encapsulation of NPs (c-SMSI).^21,191–194^ Usually, reducible metal oxides exhibit stronger c-SMSI than non-reducible oxides, which is another indication of the influence of strong oxygen vacancies on MSI. For example, Zhong and co-workers recently showed that oxygen vacancies in reduced TiO_2_ boosted MSI between Cu and TiO_2_.^195^ The TiO_2_ support was pre-reduced in H_2_ under high pressure at different temperatures to generate oxygen vacancies with different concentrations prior to depositing Cu. The advantage here is that this method provided small openings (a perforated surface), which immobilized the Cu NPs on the catalyst surface for increased stability through the c-SMSI effect while maintaining accessibility of the active phase to the reactants. Cu surface coverage could be tuned by changing the H_2_-reduction temperature. It has been shown that oxidation can also generate c-SMSI.^26,196^ We may generalize, therefore, that the generation of c-SMSI involves a reductive or oxidative treatment at elevated temperatures.

One of the characteristics of c-SMSI is its (partial) reversibility upon reduction–oxidation–reduction (ROR) treatments, thereby changing the degree of reduction, the number of oxygen vacancies, and the surface energy of catalyst constituents. Van Bokhoven and co-workers have studied the response of NPs to redox conditions in Pt/TiO_2_.^96,113,197^ They showed that the TiO_*x*_ overlayer, formed by high-temperature reduction, is stable in *in situ* transmission electron microscopy (TEM) under both pure H_2_ and pure O_2_ atmospheres. The introduction of H_2_ into a flow of O_2_ resulted in the destabilization of the TiO_*x*_ encapsulation layer and its total retraction from all particles. The overlayer coverage was restored and the system returned to a steady state after switching gases back to pure oxygen.^197^ The extent of overlayer surface coverage can be controlled by means of treatment temperature. De Jong and co-workers adopted the ROR process for a CoO/TiO_2_ catalyst. The catalyst was first reduced at 350 °C, which resulted in the c-SMSI state, followed by oxidation at 200 °C and a subsequent reduction at 220 °C to control the degree of suboxide coverage. In this way, the accessible metallic surface area saw a two-fold increase, and thus, the catalytic activity was improved with respect to direct reduction.^198^

Typically, MO overlayers are formed at elevated temperatures, under conditions that are generally applied as activation treatments. However, overlayers can also be formed, removed, or altered during the course of a catalytic reaction.^199–203^ For example, Monai *et al.*^204^ studied TiO_*x*_ overlayer formation on Ni in Ni/TiO_2_ catalysts, and their evolution under CO_2_ hydrogenation reaction conditions in a mixture of H_2_ and CO_2_. They found that the thin TiO_*x*_ overlayers formed during reduction in H_2_ at 400 °C were completely removed under CO_2_ hydrogenation reaction conditions, while those formed under more strongly reducing conditions (600 °C) were partially preserved. Adsorbates (*e.g.*, reaction intermediates) may induce the covering of the metal NPs by supporting suboxides *via* a change of the catalyst particle surface structure. As with the conventional methods for overlayer formation, adsorbate-mediated c-SMSI can be reversed by heating in air or changing the redox properties of the reactant mixture. Matsubu *et al.*^29^ observed that in CO_2_ hydrogenation over Rh/TiO_2_ and Rh/Nb_2_O_5_ catalysts. HCO_*x*_ reaction intermediates, formed when treating the catalyst in the reaction gas atmosphere at 250 °C, enabled the formation of an amorphous overlayer containing a mixture of Ti^3+^ and Ti^4+^ on the surface of Rh NPs. The overlayer formation was explained by oxygen vacancy formation due to HCO_*x*_ intermediate coverage of the catalyst surface. TiO_*x*_ overlayer formation was also observed over Pd particles over the course of the 2-propanol decomposition reaction due to reactant adsorption and partial reduction of the support.^203^

As with EMSI, changing the electronic structure of the MO support by means of doping was considered a facile method to change the degree of c-SMSI. It has been reported that changing the surface structure of TiO_2_ by doping with boron tuned the observed c-SMSI effect, leading to the formation of a slight TiO_*x*_ overlayer, which enhanced the Ni dispersion on the support.^152^

#### 5.2.2. Other methods of c-SMSI generation

Encapsulation of AM NPs by support suboxides usually occurs at relatively high temperatures (>300 °C). The temperature can be lowered, however, by intentional or unintentional coverage of the AM surface by support oxide metal cations during wet chemical synthesis or catalyst treatment. For example, Zhang *et al.*^31^ demonstrated the formation of c-SMSI during synthesis of Au/TiO_2_. TiCl_3_ species were deposited on the surface of Au at room temperature. Ti^3+^ cations acted as a strong reductant and interacted with the surface of Au species to result in negatively charged Au (Au^*δ*−^). This facilitated the formation and diffusion of thin suboxide layers of TiO_*x*_ on the Au surface. Another example of lower temperature encapsulation was demonstrated by Zhang *et al.* by a ball-milling treatment of a physical mixture of Pd and TiO_2_.^205^ The formation of oxygen vacancies during the process was claimed to be the key step driving encapsulation. It was also shown that the interface between rutile and anatase facilitated encapsulation by decreasing oxygen vacancy formation energy in comparison to pure rutile or anatase phases. It is worth noting that this method of c-SMSI generation has thus far only been possible for a handful of supports which can be reduced under mild conditions or otherwise fulfill the conditions under which c-SMSI could occur, *i.e.*, with specific redox properties and specific difference in work function between the oxide and AM constituents. Atomic layer deposition was used to generate ZnO overlayers on Co/SiO_2_ catalysts at 150 °C.^206^ Active cobalt carbide which displayed improved stability when covered with ZnO formed under syngas atmosphere.

Suboxide formation was also demonstrated for TiO_2_ during ultraviolet (UV) light irradiation in numerous studies. Fernandez *et al.* first demonstrated photo-assisted generation of c-SMSI in Rh/TiO_2_ catalysts at low temperature in 1993.^207^ They observed a reduction of the c-SMSI induction temperature down to 200 °C due to exposure of the Rh/TiO_2_ precursor to a UV treatment. The effect was attributed to the partial reduction of the TiO_2_ support by atomic hydrogen formed on the *in situ* photogenerated small Rh particles in a water/isopropanol mixture. Similarly, Rh and Pt metal particles deposited on TiO_2_ by means of photoreduction exhibited c-SMSI, as was evidenced by XPS depth analysis of Rh/Ti and Pt/Ti ratios.^208^ Chen *et al.* also achieved the encapsulation of Pd NPs with a TiO_*x*_ overlayer and suppression of CO adsorption upon UV irradiation.^191^ The mechanism is believed to involve the generation of photo-induced electron–hole pairs and the formation of Ti^3+^ species, oxygen vacancies O_v_, and interfacial Pd–O_v_–Ti^3+^ sites, affording c-SMSI in Pd–TiO_2_. It was found that the suboxide overlayer can be removed by oxidative high-temperature treatment and induced again by means of UV treatment, and the procedure could be extended to Pd/ZnO and Pt/TiO_2_.^141^

Not only high-energy UV light can be used to induce overlayer formation. Near-infrared ultrafast laser impulses were used by Zhang *et al.* on a Pt/CeO_2_ system to demonstrate CeO_*x*_ suboxide diffusion on the surface of Pt NPs.^209^ It was proposed that high-intensity confined fields at the interface between the oxide support and supported metal particles were responsible for oxygen vacancy formation. The possibility of c-SMSI with “non-reducible” oxides was also shown in the work.

## 6. Characterization of MSI-related properties

The influence of MSI on the catalytic properties of heterogeneous catalytic materials is widely accepted across the literature. However, the diversity of observed phenomena, including the strong intercorrelations of different physicochemical parameters causing such effects, as well as the often-encountered lack of definition or characterization of such terms, complicates the imagination of any unified theory able to address all aspects of MSI. This is exemplified by the spread of works in the field with a more qualitative character.

**Fig. 18:**
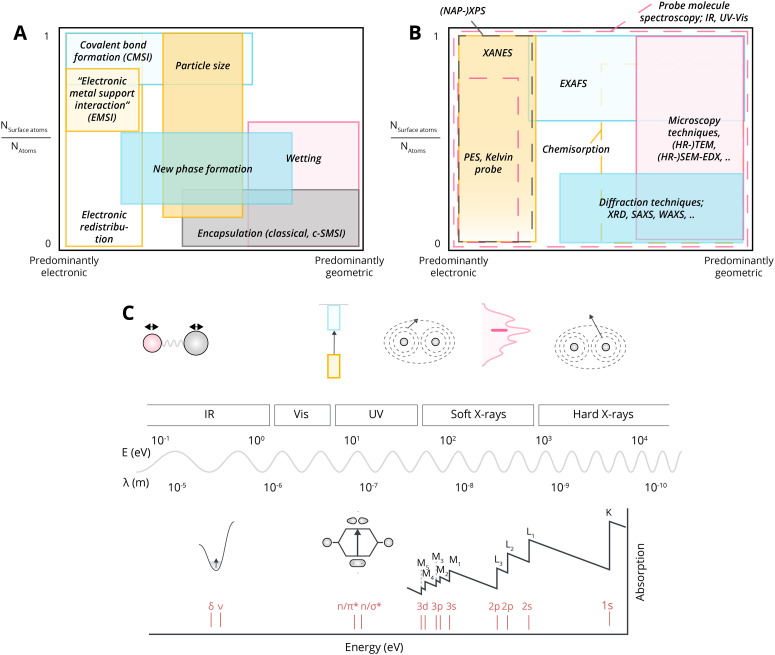
(A) Characteristics of MSI as a function of the dispersion, and the predominant observed effect (electronic, geometric). Electronic redistribution, as a predominantly electronic effect, is observed for particles of different sizes with EMSI reported for nanoparticles and clusters, and CMSI separately distinguished for SACs due to the high influence of local coordination by support atoms, *i.e.*, by geometric effects. Higher influence of geometric effects, such as local coordination, redistribution of atoms, and change of particle morphology is observed in case of the influence of MSI on particle size, new phase formation, encapsulation, and wetting behavior. (B) Characterization techniques along the same axes as in panel (A) which can be used to characterize such properties. Photoemission spectroscopy (PES), Kelvin probe, X-ray absorption near edge spectroscopy (XANES) and near ambient pressure-XPS are used to characterize the electronic structure of the materials and charge redistribution; diffraction and microscopy techniques are more used to characterize geometric restructuring induced by MSI, while probe molecule spectroscopy techniques are used for various kinds of characterizations. (C) Schematic representation of the electromagnetic spectrum, used in different techniques for MSI characterization, in wavelength and eV, as well as the probable transitions corresponding to this spectrum, from vibrational excitations, to electronic, and core-hole.

We have thus far established that the degree and nature of the observed MSI effect is dependent largely on the electronic properties of the metal and MO support. The observed effects span from predominantly electronic for smaller metal particle sizes, to having a larger possible contribution to nanoparticle morphology and catalyst reconstruction more closely connected to geometric effects. Encapsulation or/and new phase formation at the interface of metal NPs and support can occur, which is strongly affected by the values of the metal particle work function, as well as the enthalpy of formation of the oxide of the metal. Particle size and shape are also affected by MSI. We understand that this entire picture is highly complex and dynamic, and infinite variations exist, leading to the expression of MSI in different ways. Yet, we would preferably still be able to characterize not just the type, but also the extent of MSI. This line of thought will be followed in this section, starting with measurement methods for characteristics directly describing the AM–MO interaction energy, as well as descriptors of electronic structure, means to classify the quantity of electronic redistribution, the formation of new phases, particle size and geometry, and finally, overlayer formation (encapsulation, c-SMSI), see Fig. 18. The discussed possible methods of MSI quantification aim to help make our conclusions more rigorous and the corresponding theory more holistic.

### 6.1. Direct measurement of MSI strength

The interaction energy between a metal atom and oxide support can be measured experimentally as a heat of adsorption by means of microcalorimetry. The technique allows measuring interaction energies for vast classes of materials; however, the drawback of the method is a lack of structural information about the material at different metal coverages. The technique, for example, was used in the study of Cu/MgO, Cu/MoO_*x*_, and Cu/WO_*x*_ systems.^210^ The dependency of adsorption energy on metal coverage had a non-trivial nature; however, in all three cases, the energy of Cu sublimation was approached. The results proved that the Cu–Cu interaction was stronger than that of the Cu–MO bonds in all of the systems, but it did not reveal the reasons for the observed complex interaction energy *vs.* coverage dependence. The technique was further improved by the group of Campbell and was considered, in combination with He^+^ low-energy ion scattering, as a means to determine AM–MO adhesion energy.^211^

Adhesion energy can be determined from precise knowledge of nanoparticle morphology and surface energies using Wulff construction.^212^ Worren *et al.* studied Cu clusters grown on an Al_2_O_3_ surface using scanning tunneling microscopy.^213^ They observed the formation of hexagonal metal clusters of 2–5 nm width. Using Wulff construction and theoretical values of surface energies for different crystal facets of Cu, they calculated the adhesion energy to be 2.8 J m^−2^. The shape of crystals and width-to-height ratio can also be found using HRTEM. Giorgio *et al.* studied Au deposited on TiO_2_ and MgO and revealed that adhesion energy, calculated using Wullf-Kaishev equations, was higher for TiO_2_ supports.^214^ One of the benefits of using HRTEM is its capability to use environmental cells, which enables the study of variations in adhesion energies and corresponding alterations in the shape of supported NPs under different temperatures and environments. This was demonstrated in the research.^215^

Surface interfacial energy can also be determined using the Young–Dupre equation, for which knowledge of contact angle between metal and oxide, as well as surface energies of metal and oxide, must be known. An estimation of the adhesion energy between Au particles and TiO_2_ support was made by Zhang *et al.* using scanning electron microscopy (SEM).^216^ Au was deposited on the surface of TiO_2_ under ultra-high vacuum conditions. The average thickness of metal particles was monitored using a quartz crystal monitor in proximity to the sample. The average width of particles was determined using SEM. Using information about the average height and width of particles, the authors estimated the contact angle and then estimated the interfacial energy.

### 6.2. Charge redistribution measurement

**Fig. 19:**
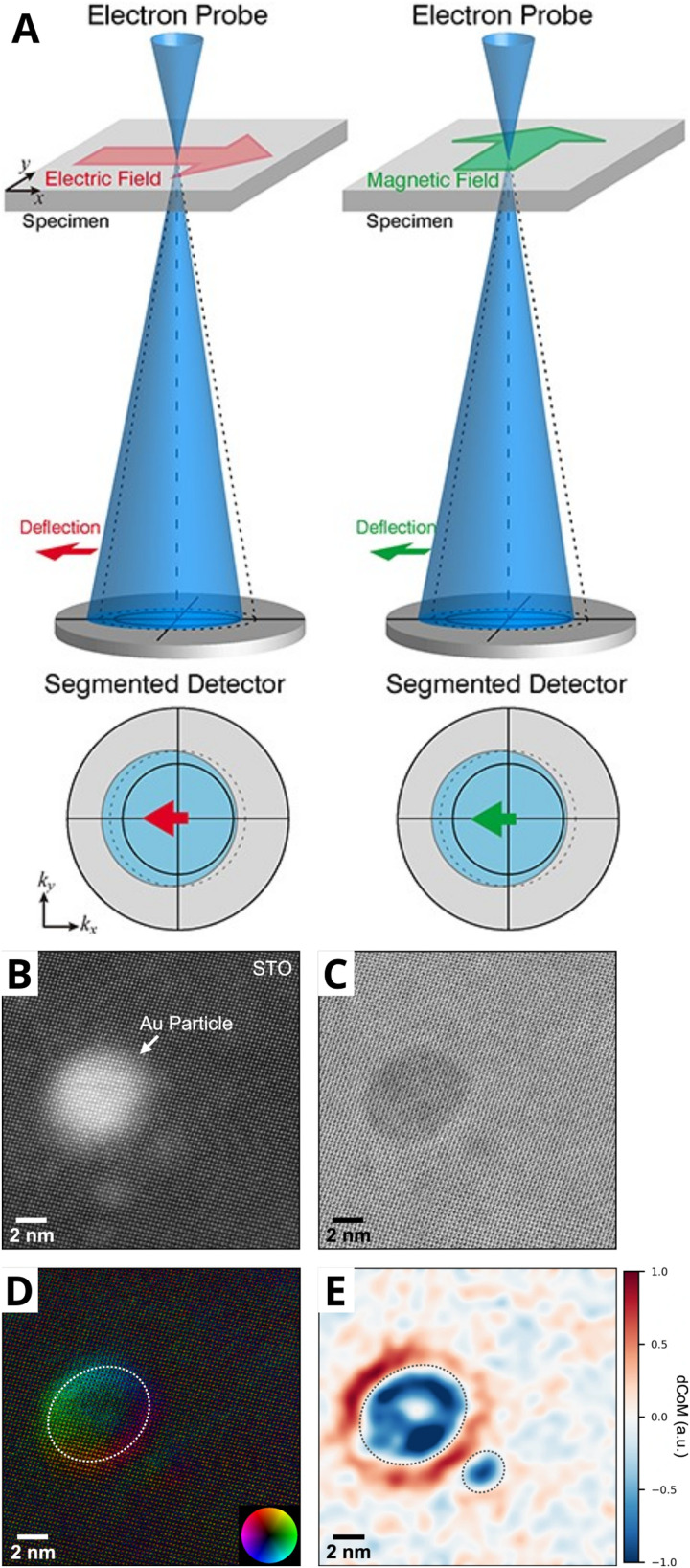
(A) Schematic illustration of DPC STEM for the visualization of electric (left) and magnetic (right) fields and their effects on the center of mass of the electron beam. Adapted from ref. 221. Copyright 2021, Oxford University Press. (B) HAADF-STEM and (C) bright field (BF)-STEM images reconstructed from the 4D data. (D) Atomic-scale center of mass (CoM) map with direction and strength indicated by color and intensity, respectively (direction inverted from raw CoM to display features associated with electric fields appropriately, since the beam electrons are negatively charged). (E) Inverted dCoM map after application of a 4 Å Gaussian filter to isolate nanometer-scale features from the underlying atomic-scale information The particles appear negative in projection, with the surrounding support positive. Dashed lines represent the approximate particle perimeters. Adapted from ref. 220.

The degree of charge redistribution is arguably the descriptor that best describes the breadth of expected MSI, as explained in Section 3.1, and will also be shown in Section 7.2. Conventional high-resolution scanning transmission electron microscopy (STEM) images can be used to provide valuable atomic-scale information. However, the technique is not typically sensitive to properties related to extended electrostatic fields. Multi-dimensional STEM (*e.g.*, 3D- or 4D-STEM) can overcome this limitation, allowing for the visualization of internal electric fields and corresponding potentials and charge densities, making it a particularly powerful technique to characterize MSI effects.^217–219^ In 4D-STEM, the center of mass of the electron beam intensity probes the momentum transfer from the specimen to the electron probe and, as such, can give insight into internal fields (Fig. 19A). Recently, Zachman *et al.*^220^ deployed HAADF-STEM to observe the atomic-level structure and sub-nm scale charge distribution in an Au/SrTiO_3_ model catalyst system. In this system, Au NPs were found to be negatively charged, while a positively charged region extended from the Au particle edge over ∼2 nm of the surrounding SrTiO_3_ support, as would be expected to compensate for the transfer of negative charge to the particle (Fig. 19B).^220^ DFT calculations confirmed charge transfer from the SrTiO_3_ support to the Au NP, leading to an overall negative charge on the particle and a positive charge on the support. The technique, differential phase contrast scanning transmission electron microscopy (DPC STEM), has the advantage of visualizing electric field distribution on an atomic scale, thus being a promising technique for mechanistic studies. It is still, however, in its infancy and several limitations were highlighted in a recent review on the topic.^221^

**Fig. 20:**
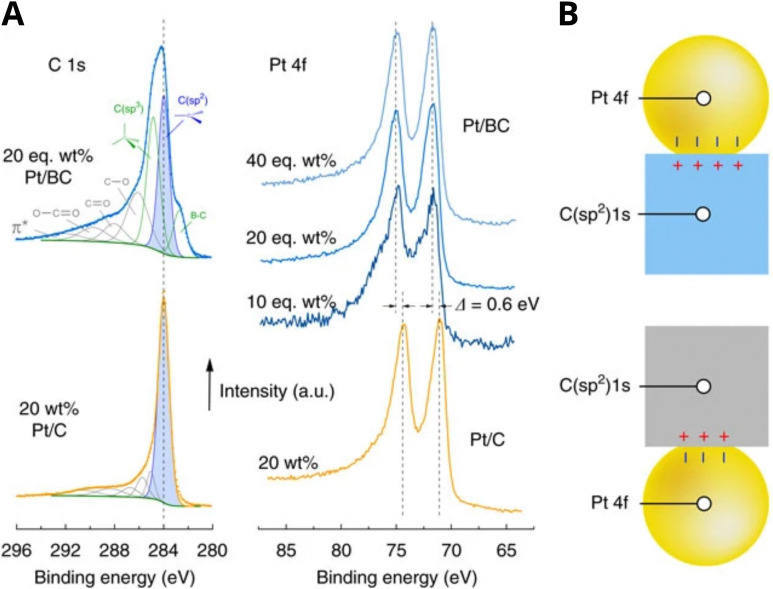
(A) XPS spectra of the C 1 s and Pt 4f region of the Pt/C and Pt/BC catalysts. Spectra are aligned to the graphitic C (sp^2^) peak at 284 eV; (B) schematic of charge transfer across the support/catalyst interface due to Fermi level equilibration rationalizing relative shifts in the XPS Pt 4f and C 1s core levels. Adapted from ref. 222.

Several examples of the use of XPS to show charge redistribution in heterogeneous catalysts exist. For example, shifts in the core binding energy in the spectrum of Pt 4f were used to demonstrate electron transfer from carbon and boron carbide supports to Pt particles.^222^ The authors observed a negative shift of 0.6 eV of the Pt 4f peaks in Pt/BC with respect to Pt/C, while the peak position corresponding to C 1s sp^2^ did not change (Fig. 20A). The observed change was attributed to a charge transfer towards the Pt nanoparticle from the BC support (Fig. 20B). Up to 0.5 eV electron binding energy shifts of Ti 2p and O 1s peaks were observed for NiFe/TiO_2_ catalysts prepared by different methods.^171^ The authors explained the observed shifts by either electron transfer from the support to the metal for samples with higher electron binding energy, or electron transfer from the metal to the support for samples with lower electron binding energy. Usage of the method is limited, however, due to the complications originating from charging and differential charging effects for insulating supports.

X-ray absorption near edge structure (XANES) analysis can be used to probe differences in electronic properties due to MSI. Changes in the intensity of the absorption edge peak, also called the white line (WL), in the width of the WL, or shift in the energy of the absorption edge are the main features of XANES spectra that are usually considered to be good descriptors of electronic characteristics of AM/MO systems. Integration of the area under the XANES features, for example, is a useful indicator of the WL intensity. At the L-edge (excitation of 2s or 2p electrons) of transition metals, the area under the WL corresponds to the density of unoccupied d-states. Consequently, it is sensitive to changes in oxidation state and variations in electronic structure resulting from chemical bonding or charge transfer processes.^223^ Ambiguity, however, exists in the literature of whether to assign changes in XANES spectra to adsorbate or charge transfer effects. Behafarid *et al.*^224^ studied a Pt/Al_2_O_3_ system in H_2_ atmosphere at different temperatures and were able to decouple the effects of adsorbate and support at 648 K because of the lowest effective residual hydrogen coverage at this temperature. Energy shifts at high temperatures (relative to the Pt foil) were plotted against the number of AM atoms in contact with the support. A linear correlation was found, with the most significant shifts corresponding to nanoparticles with 2D shapes, such as those with the largest interfacial areas.

CO chemisorption coupled with Fourier transform infrared spectroscopy can yield additional information on the electronic state and interfacial charge transfer between metal particles and a support material.^225^ For example, bands of CO adsorbed on exposed metallic Cu (2112 cm^−1^) and Cu oxides (2177 cm^−1^) were both present in the Cu/LaTiO_2_ system after reduction.^225^ After a subsequent oxidative treatment, both bands exhibited a blue shift, indicating that electrons reversibly transferred from support to metal.

**Fig. 21:**
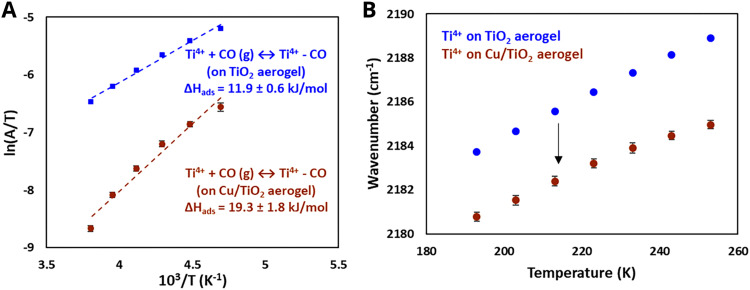
(A) Van’t Hoff plot of variable temperature IR spectroscopy (VTIR) data to evaluate Δ*H*_ads_ of CO on Ti^4+^ for TiO_2_ aerogel support (blue circles) and Cu/TiO_2_ aerogel (brown squares). (B) center of CO–Ti^4+^ IR peak (in cm^−1^) *vs*. temperature in VTIR experiment for both Cu/TiO_2_ and TiO_2_ aerogels. Error bars indicate 1 standard deviation from triplicate measurements. Error bars smaller than the symbols are not visible. Adapted with permission from ref. 123. Copyright 2020, American Chemical Society.

Maynes *et al.* investigated EMSI effects over Cu/TiO_2_ catalysts using CO as a molecular probe to study charge transfer between metal and support.^123^ They observed that cations with an electron configuration similar to a group 18 element, such as Ti^4+^, lack d-shell electrons. As a result, they do not display π-electron back bonding with CO adsorbates. Therefore, interactions between CO and such sites occur through electrostatic and σ-bonding interactions. Specifically, charge transfer happens from the lone pair on the carbon end of CO to the metal cation in a σ-coordination. This interaction increases the *ν*(CO) frequency compared to that of gas-phase CO. On the other hand, metal cations with partially filled d orbitals can donate electron density to the 2π* antibonding orbitals of CO. This donation decreases the force constant of the C–O bond and increases the C–O bond length, leading to a reduction in the *ν*(CO) stretching frequency compared to the gas-phase molecule. Metal sites often exhibit a synergistic effect from these two charge transfer mechanisms. CO adsorption itself is thus a method to probe the nature of (CO) binding sites on the surface of a catalyst, which, in turn can yield information about the available sites for reaction. The same authors used variable temperature infrared (IR) spectroscopy to investigate the effect of MSI.^123^ They measured CO adsorption energy on Ti^4+^ sites and found that it is stronger in the presence of Cu, which they attributed to EMSI effects (Fig. 21).

### 6.3. Electronic structure characterization

As alluded to throughout the article, the description of MSI should begin with an understanding of the electronic structure of the AM component and the MO support. Parameters related to the electronic structure that are important for characterizing MSI include valence and core electron properties, conductive (insulating) properties such as the CBM, VBM, and band gap, and finally, those relating to the work function and surface energy. It is therefore important to briefly outline the primary techniques used for characterizing the electronic structure of metals and metal oxides, bearing in mind that most of these methods were originally developed, and still most widely applied, in semiconductor physics.

**Fig. 22:**
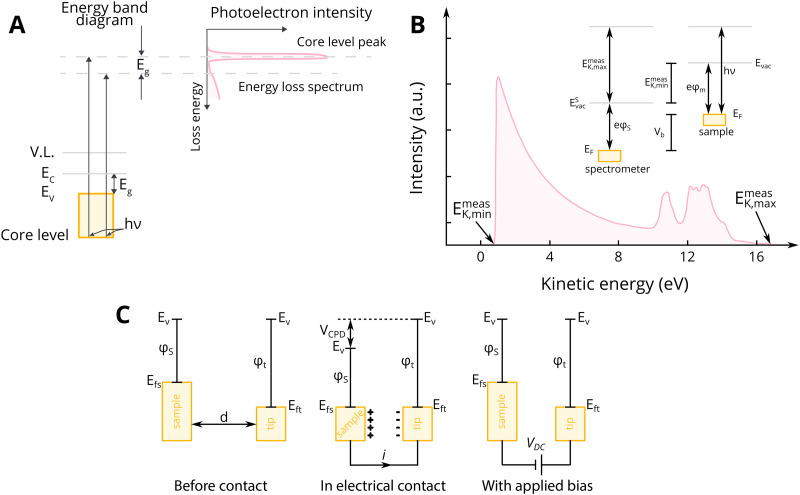
(A) Schematic view of the energy loss process of the photoelectrons from core levels. Adapted with permission from ref. 229. Copyright 2015, Japanese Journal of Applied Physics. (B) He Iα (*hν* = 21.22 eV) valence band spectrum of an Ar^+^ sputter-cleaned Au film on Si(100). The spectrum was collected with a photoelectron take-off angle (*θ*) of 90° and with a −10 V bias (*V*_b_) applied to the sample. The kinetic energy scale has already been corrected for the applied bias. The high intensity peak at low kinetic energy corresponds to the SEC region of the spectrum. *E*^meas^_K,max_ (Fermi level) and *E*^meas^_K,min_ are as shown. The inset of the figure shows the corresponding schematic energy level diagram for the sample and spectrometer. Adapted with permission from ref. 231. Copyright 2010, Elsevier. (C) Electronic energy levels of the sample and AFM tip for three cases where tip and sample are separated by a distance with no electrical contact; tip and sample are in electrical contact; and external bias is applied between tip and sample to nullify the CPD and, therefore, the tip-sample electrical force. Adapted with permission from ref. 232. Copyright 2011, Elsevier.

The most widespread technique to determine the band gap of semiconductors is from optical absorption measurements. The simplicity of the equipment and experimental setup likely led to its prevalence in the literature. In this technique, a linear and steep increase in the Tauc plot (typically a change in absorption coefficient plotted as a function of the photon energy) of the absorption spectrum is extrapolated to the point where it intersects the *x*-axis. This point represents the onset of the transition of an electron from the valence band to the conduction band and corresponds to the band gap value. Both the construction procedure and limitations of this technique are well-described in a recent review.^226^ The accuracy of the method is largely determined by the proper choice of the linear region which can be as narrow as 10 meV.^227^ To address this issue, Zanatta derived a new expression to fit absorption spectra using the sigmoid-Boltzmann function and showed that it presents a more accurate way for band gap determination.^227^ The values of the band gap of thin oxides can also be determined by analyzing the energy loss signals of O 1s photoelectrons.^228^ The photoexcited electrons suffer inelastic losses due to plasmon and band-to-band excitations. On the spectrum, one can generally see a broad peak corresponding to the electron inelastic plasmon loss, which is 20–25 eV away from the core level energies. The beginning of electron excitation from the valence band to the conduction band can also be detected at an energy level separated by the band gap (*E*_g_) from the core level peak, which can be used to determine the band gap value (Fig. 22A).^229^ Miyazaki used the technique to determine the band gap value of SiO_2_ film grown on Si monocrystals.^230^

Secondary electron cutoff (SEC) in ultraviolet photoemission spectroscopy (UPS) can be used to determine the work function of oxides.^168^ In the technique, the work function is determined as in eqn (5):^231^

5 *eϕ*_m_ = *hν* − (*E*^meas^_K,max_ − *E*^meas^_K,min_)

where *E*^meas^_K,max_ is the maximum measured kinetic energy of an electron emitted from the Fermi level, and *E*^meas^_K,min_ is the minimum measured kinetic energy in the photoelectron spectrum (*i.e.*, the zero of the kinetic energy scale relative to the sample), as shown in Fig. 22B.

For semiconductors, the Fermi level typically falls within the band gap. This means that it must be measured from a metal sample that is in electrical contact with the semiconductor sample, where the Fermi levels are aligned.^231^

The work function can also be measured using Kelvin probe force microscopy. As an atomic force microscopy (AFM) tip approaches the sample surface, an electrical force arises between them due to differences in their Fermi energy levels. Fig. 22C illustrates the energy level diagram for the tip and sample surface. When separated, the vacuum levels are aligned, but the Fermi energy levels differ. For equilibrium, the Fermi levels must align if the tip and sample surface are close enough for electron tunneling. Once in electrical contact, the Fermi levels will align through electron flow, bringing the system to equilibrium and causing both the tip and sample surface to become charged and the Fermi energy levels to be aligned, but the vacuum energy levels are no longer the same. An electrical force acts on the contact area which can be nullified. When an external bias of equal magnitude but opposite direction is applied, it neutralizes the surface charge at the contact area. The amount of external bias needed to cancel out the electrical force equals the difference in work function between the tip and the sample. Consequently, if the work function of the tip is known, the work function of the sample can be determined.^232^

**Fig. 23:**
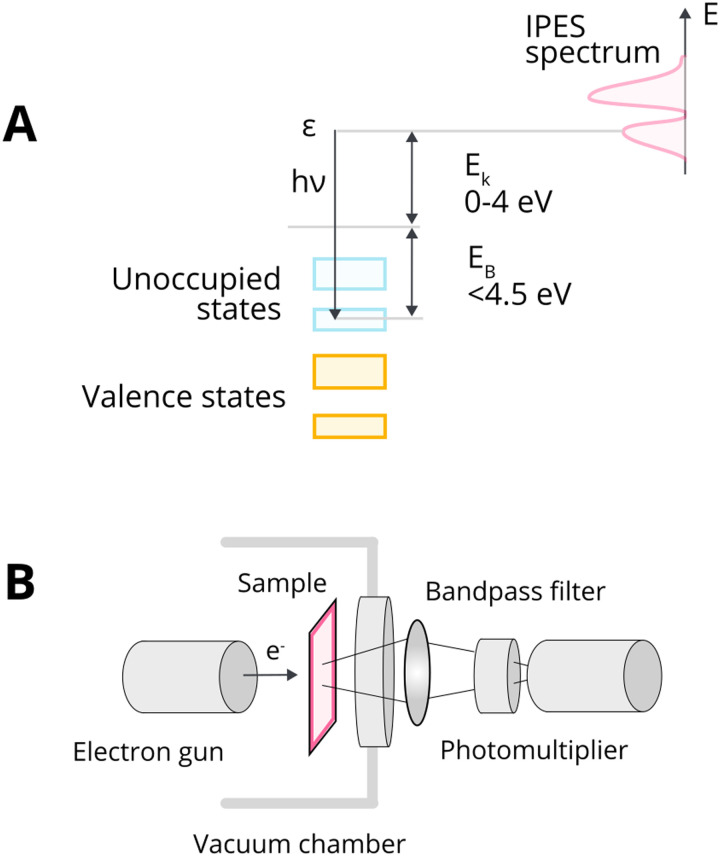
(A) Energy level diagram of inverse photoemission spectroscopy. Typical energy values for the measurement in the near ultraviolet region are also indicated. (B) Schematic diagram of the experimental setup which implements the concept displayed in panel A. Adapted with permission from ref. 235. Copyright 2012, Elsevier.

The unoccupied band structure of a semiconductor can be studied using inverse photoemission spectroscopy in which an electron, having kinetic energy *E*_k_, is introduced to the sample, and the energy of the evolved photon upon electron relaxation is measured (Fig. 23(A and B)).^233^ The two techniques, UPS and inverse photoemission spectroscopy, are often used together to get a comprehensive picture of the conduction band of inorganic semiconductors.^234^

**Fig. 24:**
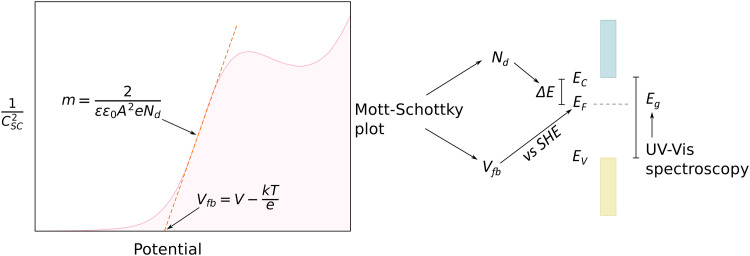
Schematic diagram of constructing the band-edge position *via* Mott–Schottky at a single-frequency analysis. Adapted from ref. 236.

Besides work function and MO band gap, the VBM and CBM positions of an oxide support material are of great importance. The widespread method of band edge position determination in semiconductor physics is based on flat band potential measurement by means of Mott–Schottky analysis. The method is based on electrochemical impedance spectroscopy measurement of a semiconductor/electrolyte interface and determination of space charge capacitance. As a result, the flat-band potential and charge carrier density can be obtained, and the position of the CBM can be calculated as schematically illustrated in Fig. 24, using the Mott–Schottky equation (eqn (6)) for undoped or low-doped n-type semiconductors:^236^6
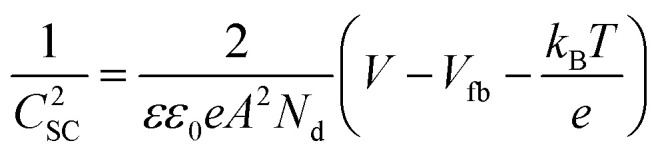
where *C*_SC_ – space charge capacitance, *ε*, *ε*_0_ – permittivity of MO semiconductor film and vacuum, respectively, *e* – electron charge, *A* – exposed surface area, *N*_d_ – charge carrier density, *V*_fb_ – flat-band potential, *k*_B_ – Boltzmann constant, *T* – absolute temperature.

However, common pitfalls should be avoided using this method as recently described.^157^ UPS^155^ and photocurrent onset potential^158^ are also used for this purpose, although these characterization techniques are not used in heterogeneous catalysis studies.

### 6.4. Alloy and mixed oxide characterization

The formation of mixed metal oxides or alloys between the AM and the metal of the support is a commonly observed phenomenon in MSI. X-ray-based characterization techniques are utilized to study such phenomena. X-ray absorption spectroscopy can offer a fundamental understanding of the local coordination environment and structure of supported metal catalysts. For example, Wu *et al.* performed DFT simulations combined with extended X-ray absorption fine structure (EXAFS) fitting to provide insights into the interactions between Pd and Fe_3_O_4_.^159^ The authors employed wavelet transform EXAFS to differentiate between Pd–Pd and Pd–Fe bonds, and used this information to further confirm alloy formation at the interface by means of DFT calculations. The continuous Cauchy wavelet transform (CCWT) provides a means of visualizing the XAS results in three dimensions: the wavevector (*k*), the interatomic distance uncorrected for phase-shifts (*R*′), and the CCWT modulus (representing the continuous decomposition of the amplitude terms).^237,238^ Interpreting these complex-valued results can be challenging, and incorrect or overly simplistic interpretations can lead to misleading conclusions. Findings should always be validated with other methods or datasets, and sensitivity analysis should be performed on parameter choices to ensure robustness of the X-ray scattering techniques, like wide angle X-ray scattering (commonly known as X-ray diffraction, XRD) or small angle X-ray scattering, which are sensitive to relatively long-range periodicity. Small crystallites (*i.e.*, smaller than 2–2.5 nm, containing a few hundreds of atoms) or amorphous particles cannot readily be detected with XRD; however, the technique is highly sensitive to the formation of crystalline phases. For example, the formation of an alloy resulting from the strong interaction between the metal atom and the cation of the support oxide can be detected, given that its quantity is more than several percent.^96,153,190^ Beck *et al.* examined the behavior of a Pt/TiO_2_ catalyst in an oxidation-reduction gas environment with, among other techniques, *in situ* XRD.^96^ Under an oxidative atmosphere, the Ti surface layer oxidized and locally formed a Pt–Ti alloy with the Pt particle. A shift in the Bragg reflection of Pt could be observed towards higher angles after reduction at 600 °C, as the incorporation of Ti into the Pt crystal structure caused lattice contraction. Moreover, the average unit cell contracted from 3.921 to 3.919 Å after reduction at 600 °C, based on Pawley fitting (fitting of the observed diffraction peaks).^190^

Raman spectroscopy is a vibrational spectroscopy technique that can detect inorganic–organic interactions that generally occur at low wavenumbers, *e.g.*, metal–C and metal–O bonds, as opposed to infrared spectroscopy. It can also be used to detect surface defects and metal–oxygen or metal–metal bonds at the molecular scale.^239^ The sensitivity of the technique can be increased by several orders of magnitude using advanced methods, *e.g.*, surface-enhanced Raman spectroscopy, or shell-isolated nanoparticle-enhanced Raman spectroscopy.^240^

### 6.5. c-SMSI characterization

By far the most discussed form of MSI is encapsulation, and several review articles specifically discuss experimental techniques for the characterization of, predominantly, this c-SMSI effect.^15,34,39,44,46,48,161,162^ The first evidence of c-SMSI was the suppression of chemisorption,^21,41,42,54^ and this method is still widely used as a characterization technique for the identification of c-SMSI in supported metal nanoparticle catalysts. In the initial approximation, the ratio of hydrogen adsorbed per metal atom on the catalyst (H/M) to the hydrogen adsorbed per metal atom when the catalyst is reduced at low temperatures can be used as an approximate measure of the extent of metal encapsulation, noting that not all exposed sites may adsorb the same quantity of H.^182^ Zhao *et al.* observed that H/Pt and CO/Pt ratios decreased from 0.24 to 0.04 and from 0.13 to 0.03 for catalysts reduced at 200 and 500 °C, respectively, demonstrating the suppressed chemisorption of H_2_ and CO, attributed to metal surface coverage by suboxide overlayers.^154^ The “ease” of c-SMSI formation for different AM/MO systems can be quantified by the rates of hydrogen adsorption inhibition over time. Hideaki showed that the H/Pt ratio over Pt/V_2_OB, Pt/TiO_2_, and Pt/Nb_2_O_5_ decreased linearly as a function of the square root of the catalyst reduction time.^241^ However, the rate of the decrease was different for the systems, and the ease of c-SMSI was deduced to be on the order Nb_2_O_5_ > TiO_2_ > V_2_O_5_.

Quantification of the c-SMSI state, as well as the tendency of one oxide to form an overlayer over another, are possible by employing a combination of chemisorption and spectroscopic techniques. Vannice *et al.* first used CO adsorption coupled with IR spectroscopy to detect c-SMSI in a Pd/TiO_2_ catalyst,^66^ and the observed decrease in adsorbed CO band intensity has since become a commonplace tool to identify encapsulation, as well as specific properties of the c-SMSI state such as the variation of exposed metal sites during pretreatments.^194,195,225,242^ For example, Zn^*δ*+^ sites were proposed by the emergence of a peak at 2125 cm^−1^ in a Cu/ZnO:Al catalyst.^242^ Raman spectroscopy was also used to characterize the encapsulation of CeO_2_-supported Pt catalysts, as the adsorbed CO peak position and intensity varied with the reduction temperature applied.^190^

**Fig. 25:**
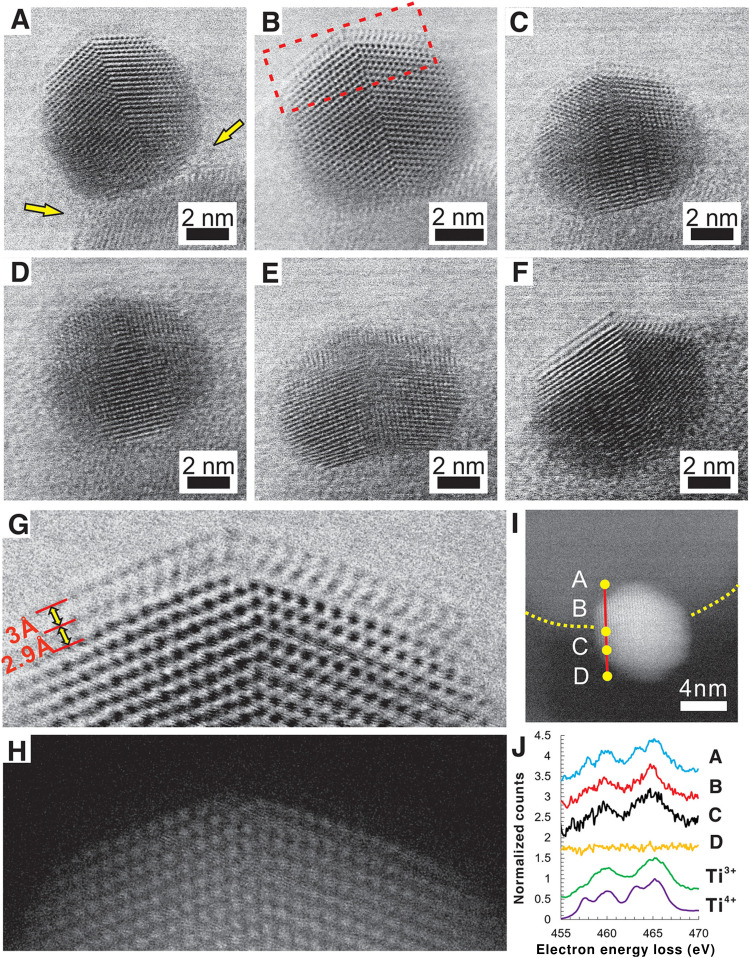
Formation and removal of TiO_*x*_ overlayers on a Pd nanocrystal in Pd/TiO_2_. Sequential *in situ* observations, first under reducing conditions (H_2_ (5 vol%)/Ar at 1 atm) of the Pd/TiO_2_ sample at 250 °C (A), then 500 °C for 10 min (B); next, under oxidizing conditions (150 Torr O_2_) at 250 °C for 8 min (C), then 15 min (D), and then at a final stable state at 500 °C (E); finally, under reducing conditions again (H_2_ (5 vol%)/Ar at 1 atm) at 500 °C for 5 min (F). (G) and (H) are higher magnification ABF and HAADF images, respectively, of a section of part (B) showing the TiO_*x*_ double layer. (I) and (J): EELS spectra extracted from a line scan of another Pd particle, shown in (I), under H_2_ (5 vol%)/Ar at 1 atm and 500 °C, and Ti^3+^ and Ti^4+^ reference spectra acquired from LaTiO_3_ and TiO_2_. Adapted with permission from ref. 141. Copyright 2016, American Chemical Society.

Electron microscopy has played a large role in the identification and explanation of encapsulation.^243,244^ Bernal *et al.*^44^ wrote a dedicated review of the study of c-SMSI interactions using electron microscopy techniques. In recent years, through the development and application of nanoreactors, *in situ* HRTEM has been applied to track the formation of overlayers at controlled elevated temperatures and in different gaseous environments.^96,141,154,197,204,245^ For example, an amorphous layer was observed to start evolving near the metal/oxide interface in a Pd/TiO_2_ system under reducing conditions (H_2_ (5 vol%)/Ar at 1 atm) at 250 °C, which then covered the surface of Pd nanoparticle after reaching 500 °C (Fig. 25).^141^ EELS, often performed together with TEM, can provide information on chemical speciation at the nanoscale, which can provide additional chemical information about the formed overlayer. *Operando* electron microscopy coupled with vibrational spectroscopy was used recently to show that the reduction temperature applied to achieve the c-SMSI state (*i.e.*, 400 *vs*. 600 °C) affects the subsequent preservation of the overlayer under reaction conditions for CO and CO_2_ hydrogenation.^204^ The catalyst reduced at 600 °C retained some of the overlayers under reaction conditions, which affected the reaction mechanism to favor carbon–carbon coupling through the provision of a “reservoir” of carbon species.

Petzoldt *et al.*^246^ investigated c-SMSI on Pt/TiO_2_ catalysts with near ambient pressure-XPS under different oxidizing and reducing conditions to study the impact of the various treatments on the TiO_2_ support. The Ti 2p doublet binding energy and shape corresponded to that of stoichiometric TiO_2_ both at room temperature and directly after reaching 527 °C. However, prolonged annealing in ultrahigh vacuum induced the formation of a shoulder at low binding energy of the Ti 2p_3/2_ peak, which was attributed to a substoichiometric c-SMSI overlayer.^246^

## 7. Effects of MSI on catalytic performance

MSI influences a catalyst from its synthesis, through activation, to its life and death (see also Fig. 17), resulting in noticeable changes in its performance. The physicochemical properties of support materials and their interactions with the AM constituent, including support surface area, phase, surface chemistry, and particle size, can all change as a function of the synthesis, activation, active phase, and deactivation. Such properties of a catalyst define MSI, and, in turn, their interrelation influences all stages of catalyst operation dynamically, and at greatly varying time scales during catalyst lifetime. It is quite difficult to compare the individual or unique effects of MSI on the catalytic performance directly. For example, it is nearly inevitable to obtain different metal particle sizes and shapes after synthesis when using equal synthesis parameters but a different support due to MSI. The comparison of the effect of support properties on the catalytic performance of such series will be influenced by exposed site differences. It cannot be related to support effects on their own. This implies that to properly study the effect of MSI on catalytic properties of interest, it is essential to tune synthesis parameters for each AM/MO pair so that the properties of interest are as comparable as possible, for example, nanoparticle size. However, the aspect ratio or shape can change significantly for similar particle sizes depending on the support or the synthesis procedure, exposing different sites or facilitating electronic redistribution differently. All these slight differences are likely to influence the observed catalyst stability, selectivity, and activity. In the below subsections, we will discuss what we believe to be the most noteworthy demonstrations thereof and recent literature explicating them.

### 7.1. Effects of MSI on catalyst stability

The stability of a catalyst constitutes its resistance to deactivation, as well as its ability to withstand possible regeneration procedures after deactivation. Deactivation is generally caused through one or a combination of three general mechanisms: first, loss of active material, for example, through coalescence, leaching, or promoter depletion; second, blockage of active sites, for instance, through poisoning, or the deposition of coke; and finally, modification of active sites *via*, for example, phase changes.^247^ All these mechanisms can be affected by different types of MSI to a different extent and should ideally be accounted for during catalyst design.

Finding the balance of high dispersion and high stability can be done by optimizing the metal–support interactions. This was done in a Co/CeZrO_4_ system by varying the size of the support particles. It was observed that support particles of intermediate particle size (*i.e.*, ∼20 nm) lead to higher dispersion of metallic cobalt compared to both smaller and larger support particle sizes. This optimum metal–support interaction led to optimal stabilization of the Co NPs during reduction, in turn leading to higher catalytic activity and stability during CO_2_ hydrogenation.^173^

The strength of MSI can affect the redox properties of the active phase, leading, for example, to reduction (or oxidation) at different temperatures during the activation step and/or during reaction. Beale and coworkers showed that Co NPs supported on SiO_*x*_/Si(100) and rutile TiO_2_(110) substrates showed markedly different reduction and sintering resistance properties.^248^ SiO_*x*_/Si(100)-supported Co NPs have weaker interaction and were fully reduced upon a H_2_ reduction treatment, whereas the TiO_2_(110)-supported counterpart was only partially reduced by the same treatment. In the former case, however, the NPs migrated and agglomerated during reaction, while for the latter the interaction was observed to be so strong that Co atoms spread at and below the surface. The concept of the induction of stronger support interactions can be manipulated to (re)gain a high degree of dispersion by performing consecutive calcination and reduction treatments either as an activation procedure or as a regeneration procedure, which was shown to improve the dispersion of NPs.^249,250^

The surface geometry of the supports can play a crucial role in the stability of the catalyst. For example, Pd NPs loaded on either a (100) (more coordinated) or (001) (more undercoordinated) facet of ZnO showed markedly different sintering properties.^251^ Pd particles on the (100) facet grew from 1.1 to 5.7 nm after calcination at 600 °C, while on the (001) facet they retained their size. This phenomenon explains the trends in the performance of the Pd catalysts in CO oxidation reaction supported on ZnO nanorods and nanosheets, where the latter had a higher degree of (001) facets and showed activity and stability enhancement.

Al_2_O_3_ is a particularly often seen example illustrating the effects of support surface chemistry on catalyst stability due to the possibility of exposing unsaturated penta-coordinated Al^3+^_penta_ to the surface, which allows for strong binding of heteroatoms.^252–254^ Al^3+^_penta_ sites have been shown by Kwak *et al.* to allow for the formation of atomically dispersed Pt sites at low loadings on γ-Al_2_O_3_ (100) facets, where the cation was believed to bind to five oxygen atoms in total, three of which from the alumina. Such undercoordinated sites, in addition to allowing for higher dispersion, also lead to higher stability. This was shown in a study where several different Pd-based bimetallic NPs were synthesized using Al_2_O_3_ with a high abundance of Al^3+^_penta_ sites, which was shown to decrease the mean metal particle size as compared to a commercial Al_2_O_3_ support, and to suppress sintering.^252^ In another study on Ru/γ-Al_2_O_3_ catalysts, an increase of Lewis basicity of the γ-Al_2_O_3_ surface was observed following a high-temperature treatment during CO_*x*_ methanation, where OH species were formed on either hexa- or penta-coordinated Al sites on the surface. Such induced Lewis basicity, combined with higher mobility of the Ru species at high temperatures, leads to the formation of flat Ru NPs with higher sintering resistance.^176^

Finally, the formation of overlayers, c-SMSI, can greatly influence catalyst stability. The balance between blocking of active surface area and stabilization, however, is delicate. De Jong and coworkers showed that for Ni/Nb_2_O_5_ catalysts, Ni particle growth during CO hydrogenation into C_5+_ hydrocarbons could be prevented by partial reduction of the Nb_2_O_5_ support.^255^ When the support was not co-reduced, deactivation occurred rapidly *via* the formation of highly mobile nickel subcarbonyl species (Ni(CO)_*x*_).

### 7.2. Effects of MSI on activity and selectivity

It is well-understood that the binding energy of specific reaction intermediates with AM surfaces typically largely determines the observed catalytic activity and selectivity in supported metal catalysts. Such binding energy is roughly correlated to the electronic properties of the metal particle, such as the d-band center of the metal surface, according to the Bell(Brønsted)-Evans Polanyi principle. Therefore, EMSI and CMSI effects on activity and selectivity arise because the orbital energies, or d-band center, and Fermi level of the AM are, in turn, strongly affected by the degree of electronic redistribution between the metal and the MO support. However, it is not only these electronic effects that can influence catalytic activity and selectivity. The geometric changes or restructuring, such as rearranging metal atoms or exposing different facets, altering particle size and shape, and forming new phases, along with c-SMSI, have correlated but distinct effects on intermediate binding energy. These effects can either positively or negatively impact the overall performance. The important distinction between the latter *versus* the former is that geometric changes can affect different reaction intermediates in a notably different manner.^20^

Electronic promotion of the ZnO support with trivalent promoters, Al^3+^ and Ga^3+^, led to a decreased apparent activation energy and reaction order in H_2_ over Cu/ZnO catalyst in the reverse water gas shift reaction, while the reverse effect was observed for Mg^2+^ doping.^256^ The promoters thus tuned the electronic properties of ZnO, which in turn affected the degree of charge transfer between the copper metal and the support material. For atomically dispersed Pt/CoFe_2_O_4_ catalysts that were soaked in water, H-bonding at the Pt–O–Fe interface resulted in reduced charge transfer from the metal to the support, showing high sensitivity of SACs to local coordination.^132^ This led to an increase in methane oxidation activity of over 50 times.

Jenness and Schmidt tried to generalize the influence of supports on surface reaction elementary step thermodynamics for Rh-based Fischer–Tropsch catalysts.^177^ They stated that if *N*_products_ > *N*_reactants_ (*e.g.*, a dissociation reaction), and the support tends to increase adsorbate binding with respect to the pure metal case, the exothermicity of the corresponding elementary step will increase. The same support will decrease the exothermicity of association reactions. The tendency of a particular support to strengthen or weaken adsorption will depend on MSI and the degree of charge redistribution. A negative effect of MSI was shown for Au/TiO_2_ catalysts used in CO oxidation, where decreased reaction activity was observed with an increased presence of bulk oxygen defects, which the authors argue result in electronic redistribution that lowers the CO adsorption strength.^257^ Similar effects were observed for Pd NPs on Mo-doped TiO_2_ supports, which showed a decrease in the CO_2_ methanation rate with respect to Pd/TiO_2_.^185^

Changing the shape and size of the support particles is a way to influence the degree of MSI and, thereby, the resulting AM particle size.^173,258^ The influence of the morphology of the support on the metal nanoparticle morphology was demonstrated by studying Pd/CeO_2_ catalysts where CeO_2_ octahedrons, cubes, and rods were used. The activity in CH_4_ catalytic oxidation was found to be highest for octahedrons. It is worth noting, however, that high activity was due to the presence of the abundant Pd^2+^ sites, which was not the case for other morphologies, and that support morphology, therefore, did not directly influence the activity; structural changes in the active phase did.^259^

The above examples relate predominantly to the effect of the support on the properties of the AM constituent and how, in turn, that can influence catalytic activity and selectivity. There are, however, several examples of reactions that do not exclusively take place on the AM but in which the reaction mechanism includes species adsorbed or activated on the support. In general, catalytic systems benefit from oxygen vacancies in terms of catalytic activity, especially when the MO support is actively involved in the catalytic cycle. For example, H_2_ reduction of TiO_2_-supported catalysts can generate oxygen vacancies in the form of coordinatively unsaturated cations close to metal species, which can work in tandem with the AM constituent during catalysis.^195,260,261^ In order to tune the availability of these vacancies, CeO_2_ with different crystal shapes (rods, cubes, octahedrons, and polyhedrons) and exposed facets (100), (110), and (111) were deployed in CO_2_ hydrogenation in combination with Pd. It was found that Pd supported on CeO_2_-rods with exposed (110) facets had the lowest oxygen vacancy formation energy. As such, the catalytic activity of the CeO_2_-rod-supported Pd was highest as the abundantly available oxygen vacancies functioned as CO_2_ adsorption and activation sites.^262^ In another example, Zhong and co-workers recently showed that oxygen vacancies in reduced TiO_2_ boosted MSI between Cu and TiO_2_, forming a new interface that facilitated the activation of CO_2_ molecules and promoted the formation of the proper reaction intermediates for methanol formation.^195^

Encapsulation of metal particles by a support overlayer, *i.e.*, c-SMSI, may be expected to affect a reaction by loss of accessible active surface sites, which hinders the adsorption and reaction of reactants and intermediates.^182^ For example, high-temperature reduction treatment of a Ru/TiO_2_ catalysts resulted in a decrease in the CO methanation activity.^263^ However, several examples exist where the activity of encapsulated AM phases is increased by encapsulation.^31,264^ For example, Zhang *et al.*^195^ observed that small N_2_O molecules could penetrate the TiO_2−*x*_ surface layers on Cu/TiO_2−*x*_ catalysts and interact with the Cu NPs. They suggested the overlayer formation to be only partial or to contain cracks and pores allowing the passage of reactant and product molecules.

The reaction mechanism can benefit from the local availability of suboxide overlayers. For example, the c-SMSI overlayer in a Ni/TiO_2_ system was used to tune C–C formation in a reaction that typically only produced C_1_ products: CO_2_ methanation over nickel.^265^ The effect was later further explained by a combination of *operando* HRTEM and diffuse reflectance infrared Fourier transform spectroscopy, showing that the overlayer formed after the high-temperature reduction was restructured under reaction conditions, exposing highly active Ni/TiO_*x*_ interfacial sites.^204^ On these sites, it was proposed that the TiO_*x*_ can serve as a C-species reservoir, which can react with carbon species on Ni to yield C–C coupling products with higher intrinsic activity. The presence of an overlayer can thus impact catalytic activity and selectivity through a number of different mechanisms.^266^

### 7.3. Quantitative assessment of MSI effects

Based on the discussion, it is evident that there is a wide range of effects resulting from metal–support interactions that can impact catalytic activity. These effects include changes in the electronic structure of the active sites, alterations in the shape of the metal nanoparticle, reduction of exposed surface area due to encapsulation, phase transformation, and even active involvement of the support material in the reaction mechanism. These effects depend on specific details that are often not fully reported, such as support particle size, crystalline phase, impurity content, surface functionalization, flow path of the reactor, and so on. As a result, it is difficult to identify consistent trends related to metal–support interactions across experimental results in the literature.

**Fig. 26:**
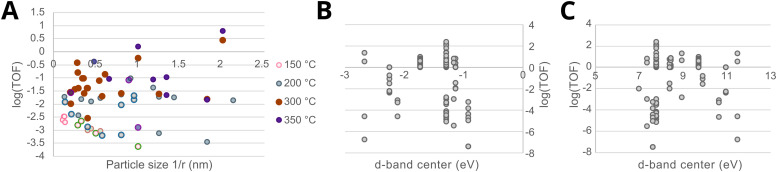
Turnover frequencies (TOF) of different supported catalysts in the CO_2_ hydrogenation reaction as a function of (A) reaction temperature and particle size (1/*r*), (B) plotted against the d-band center of the metal, and (C) plotted against the adjusted d-band center of the metal as a function of the difference between the metal work function and the conduction band minimum of the MO. (B) and (C) are normalized to the maximum value observed per *T*.

Despite the expected difficulty in comparing catalyst activity between studies in literature due to the factors noted above, we analyzed the recent literature on CO_2_ hydrogenation catalysis, compared the surface specific activities (turnover frequencies, TOF) at different temperatures, and calculated the correlation of several bulk parameters related to MSI, such as discussed in this article, and particularly in Section 3.1. In this exercise, we face two possible challenges that are not easily distinguishable: minor but important details influencing activity are not properly reported, or our models to explain the effect of MSI on activity are not entirely accurate. Nevertheless, it can possibly provide valuable insights into whether a means to rationalize MSI effects may be MSI-adjusted activity descriptors (like the d-band center adjusted for interaction with the support) *versus* non-adjusted parameters (such as the d-band center of the metal alone), as well as the extent of such contributions on activity. Surface specific activities (TOF) were gathered or calculated from experimental literature reports and are summarized in Table S3 (ESI†). Fig. 26A shows the TOF *versus* the particle size as 1/*r*, as the TOF is expected to be proportional to the number of active sites, which decreases with 1/*r* for different reaction temperatures. It is seen and well-understood that the TOF scales predominantly with the temperature. According to what was discussed in Section 3.1, it would be interesting to understand the correlation of the expected re-equilibration in electronic structure determined predominantly by the work function of the metal and the CBM of the MO. That is, when a nanoparticle comes in contact with a MO and electronic redistribution occurs, the d-band may shift, become more narrow, and become less filled, which will be dependent on the specific electronic configuration of the metal. As the d-band center of a metal (*E*_C_) is reported with respect to the *E*_F_ of the metal,^74^ and is an often cited descriptor of adsorption strength (and therefore catalytic activity), electronic redistribution due to overlap of the CBM with the *φ*_M_ should affect the d-band center of metals disproportionately for positive ΔWF (*i.e.*, low CBM relative to *φ*_M_). We can attempt to capture such a re-equilibration crudely by normalizing the d-band center energy accordingly (*E*_C-adj_).

Fig. 26(B and C) show the normalized TOF per temperature (*i.e.*, for each temperature, the maximum TOF was determined and scaled to 1), plotted against the d-band center of the metal,^74^ and the adjusted d-band center, which takes into account the possible interaction of the AM and MO. This was calculated by taking the difference in the CBM of the MO and the work function φ of the AM (ΔWF) with respect to the vacuum, after which the resulting equilibration of the Fermi level of the metal (*E*_F-MSI_) was calculated if CBM_MO_ > *φ*_M_. The adjusted d-band center was then calculated taking *E*_F-MSI_ and the reported d-band center of the elemental AM as reported.^74^

The normalization of the re-equilibrated *E*_F-MSI_ of the metal, as calculated here, is, as such, a grave oversimplification. It must again be noted here that general electronic values for the metals and supports were taken from literature, and individual outliers such as dopants or functionalization of supports are clearly not accounted for.

**Fig. 27:**
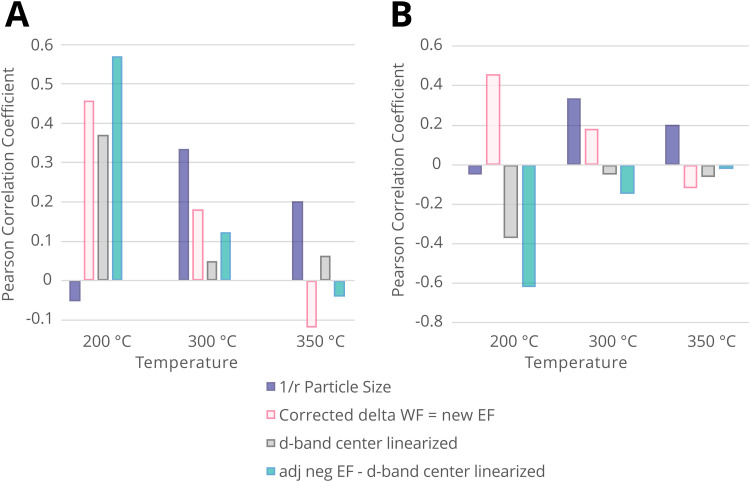
(A) Pearson correlation coefficients calculated by evaluating the surface-normalized activities for literature values of CO_2_ hydrogenation catalysts with respect to metal particle size (as 1/*r*), the d-band center (*E*_C_), and the adjusted d-band center energy (*E*_C-adj_) based on the difference in conduction band minimum of the MO and work function of the metal (ΔWF), the adjusted Fermi energy of the metal if 0 > ΔWF (*E*_F-MSI_), and the original position of the d-band center *E*_C_. (B) Pearson correlations, now calculated with the linearized (adjusted) d-band center, as the expected trend would resemble a volcano plot. The values used for the calculations are listed in Table S3 (ESI†).

Fig. 27 shows the Pearson correlation coefficients of the surface-normalized activities against particle size as 1/*r*, the d-band center *E*_C_, the *E*_F-MSI_, and the adjusted d-band center *E*_Cadj_ for CO_2_ hydrogenation activities reported at different temperatures. The d-band_adj_ has the highest of a set of poor to moderate correlation coefficients to the reported TOF at 200 °C. This indicates a moderate correlation in this parameter that connects the highest amount of different electronic MSI considerations (alignment of the metal and MO electronic structure, as well as the following probable position and adjustment of the d-band center energy), which might influence activity. *E*_F-MSI_ and *E*_C_ show a lower but still moderately positive correlation. At higher reaction temperatures, all correlations become worse, and the best correlation can be found with particle size (yet this can still only be described as a low correlation). The decrease of correlation with an increase in temperature is likely to be caused by several factors, including a larger error in the differences in the data due to measurement setups, the likely larger contribution from overlayers with diverging effects, and so forth. While particle size seems to have a higher correlation to the TOF at 300 and 350 °C than at 200 °C, it is interesting indeed that at lower temperatures, the electronic structure parameters adjusted for MSI is better correlated.^267^ The results of this exercise shown in Fig. 26 and 27 underline the need for systematic, large-scale experimental studies, which would be greatly aided by automation and data chemistry. This could allow for the further development of more accurate theory and *vice versa*.

## 8. Conclusions and future perspectives

Fundamentally, different MSI effects can arise at the ends of the particle size spectrum. For large particles, encapsulation and/or new phase formation at the interface can occur, which is significantly influenced by the metal work function and the enthalpy of formation of the metal oxide. This scenario is complex and dynamic, with MSI manifesting in diverse ways. Smaller particles, clusters, and atoms appear to display unique MSI characteristics, primarily due to the quantized nature of their energy levels.

The small number of truly varying, fundamentally different effects causing different observed MSI effects, however, as we argue, cannot support the large degree of subclassifications introduced in recent literature. Several studies cited in this Review have introduced new subclassifications of MSI. The degree of electronic perturbation of atoms and clusters led to terms such as “electronic metal–support interactions” and “covalent metal–support interactions”, which indeed are conceivably different to encapsulation – despite each of them being explained by the direction and degree of electronic interaction, be it (predominantly) covalent or ionic, or of metal nature, which is correlated to (subsequent) morphological or geometrical properties of importance (nanoparticle shape, size, phase, alloy formation, site exposure, and so forth). Further subclassifications were introduced in literature based on the type of environment or procedure that induces the observed effects (*e.g.*, oxidative treatment rather than reductive, or the influence of reactants), and additional subclassifications arose by varying what was classified as the “active” nanoparticle (metal *versus* metal oxide) and what constituted the support (metal *versus* metal oxide).

It is likely, however, that the observed phenomena can be largely understood by the degree of alignment between the work function (or orbital energies) of the metal (atom, cluster) and the conduction band minimum of the metal oxide, although such an assessment could only very primitively be done due to the large degree of variation between studies throughout the literature, as well as likely unreported experimental parameters. A large, systematic study comparing several different combinations of metals and metal oxide catalysts along several different activation step types, as well as reactions, could greatly increase our understanding of the contribution of MSI effects where automation and machine learning/artificial intelligence can play an important role. Indeed, much of the basic theory discussed in this review was developed only for a handful of systems, predominantly on TiO_2_ with platinum group metals, which, as we discussed in Section 3, is not directly fully able to explain some of the recently observed phenomena such as overlayer formation in SiO_2_.

Advances in characterization techniques have recently been demonstrated, aiding in our understanding of the observed effects. Particularly, *operando* spectroscopy and microscopy (*i.e.*, studying the reaction under realistic reaction conditions while quantifying the reaction products) techniques have advanced significantly. It has only recently unequivocally been shown that overlayers (c-SMSI) can be both unstable and completely removed during catalysis, even under mild conditions (200 °C), or (partially) sustained up to at least 400 °C. Such techniques will further help, for example, to gain a better understanding of the role and nature of charge transfer, particularly necessary for non-classical systems.

Among further possible future research directions, we suggest the following:

– Rationally influencing the activity of supported metal catalysts through MSI, from nanoparticles to the non-scalability (quantum) regime:

In catalyst systems with ever-increasing complexity, disentangling the effect of the different fundamental phenomena surrounding MSI is highly complex. They can be predominantly electronic or geometric, as mentioned – which arguably affect the exponent of the rate equation. However, it is not unreasonable to attempt to further distinguish (and manipulate) MSI effects, for example, through manipulating or dissecting the effects of confinement, which can have a (decoupled) contribution also in the prefactor of the rate equation. It is possible that in the future, researchers will be able to separate the electronic and geometric effects of catalysts in a rational way, for example, by resonance catalysis or “programmable” catalysis. This could lead to surpassing the current limitations of catalysis imposed by the scaling relationships or compensation effect, which are the often-observed linear dependencies between the catalyst activity and the prefactor or exponent.

Whether a catalytic reaction involves the breaking or formation of σ- *vs*. π-bonds traditionally determines the expected “structure sensitivity” trend, either increasing with decreasing metal particle size (for σ-bonds) or increasing with metal particle size (to a certain maximum, for π-bonds) as there is an energetic benefit to a certain degree of coordination for the cleavage or making of π-bonds, as several studies cited in this Review have shown. To optimize the utilization of “single atom catalysts” across reactions where π-bonds should be cleaved, it is crucial to properly stabilize (coordinate) these bonds with the support's sites, which gives another avenue to decouple geometric and electronic effects.

High-temperature reductive treatments usually lead to oxygen vacancy formation and/or transformation in reducible oxide support in addition to forming c-SMSI with metal nanoparticles. Manipulating oxygen defects in oxide supports is a tool to direct the electron density. This concept can be extended to the design of other (non-oxide-supported) catalysts, such as sulfur- or carbon-based materials, by engineering defect sites. However, as mentioned, to effectively do this, better methods to characterize or quantify (the contribution of) oxygen defects are needed.

– Improving characterization through theory and *in situ*/*operando* characterization:

There is a strong focus in the literature on the intrinsic properties of either the metal constituent or the metal oxide support, such as the work function. More attention can be given to, for example, dynamic adsorbate-induced effects. Theoretical models are often still limited to simple reactions and simplified systems, unlike experimental studies, in which the effects contributing to observations are difficult to deconvolute, *e.g.*, due to minor impurities or adsorbates taking part in interactions; force field-based studies focusing on mesoscale systems incorporating (dynamic) adsorbate interactions will further enhance understanding.

To fully make use of the potential of MSI in catalyst design and optimization, the dynamics of MSI and how to leverage them must be characterized and manipulated.

As a final remark, a comprehensive and coherent understanding of the factors influencing the interaction strength between metal–support pairs to an extent that allows us to tune catalytic activity through MSI from first principles, particularly in clusters and atoms, still requires significant efforts. Such generalized understanding is likely not aided by the proliferation of subclassifications. Characterizing and referring to the underlying physical phenomenon or phenomena causing the observed effects is preferred.

## Data availability

The data supporting this article have been included as part of the ESI.†

## Conflicts of interest

There are no conflicts to declare.

## Supplementary Material

CS-053-D4CS00527A-s001

## References

[cit1] de Jong K. P. , in Synthesis of solid catalysts, ed. K. P. de Jong, Wiley-VCH, Weinheim, 2009, pp. 1–11

[cit2] Wacławek S., Padil V. V., Černík M. (2018). Ecol. Chem. Eng. S.

[cit3] Chemical Industry Worldwide - Statistics & Facts, https://www.statista.com/topics/6213/chemical-industry-worldwide, (Accessed 18/8/2023)

[cit4] Perathoner S. and Centi G., Science and Technology Roadmap on Catalysis for Europe: A path to create a Sustainable Future, European Cluster on Catalysis, ed. S. Gross and E. J. M. Hensen, 2016

[cit5] Vogt C., Weckhuysen B. M. (2022). Nat. Rev. Chem..

[cit6] Van der Hoeven M. , Kobayashi Y. and Diercks R., *Technology Roadmap – Energy and GHG Reductions in the Chemical Industry via Catalytic Processes*, International Energy Agency, International Council of Chemical Associations, Dechema, 2013

[cit7] Jin R., Li G., Sharma S., Li Y., Du X. (2021). Chem. Rev..

[cit8] Thomas J. M., Harris K. D. M. (2016). Energy Environ. Sci..

[cit9] Winnacker Küchler: Chemische Technik: Prozesse und Produkte, ed. R. Dittmeyer, W. Keim, G. Kreysa, A. Oberholz, Wiley-VCH, Weinheim, 5th edn, 2004

[cit10] Ning Y., Wei M., Yu L., Yang F., Chang R., Liu Z., Fu Q., Bao X. (2015). J. Phys. Chem. C.

[cit11] Bezemer G. L., Bitter J. H., Kuipers H. P. C. E., Oosterbeek H., Holewijn J. E., Xu X., Kapteijn F., van Dillen A. J., de Jong K. P. (2006). J. Am. Chem. Soc..

[cit12] Vogt C., Groeneveld E., Kamsma G., Nachtegaal M., Lu L., Kiely C. J., Berben P. H., Meirer F., Weckhuysen B. M. (2018). Nat. Catal..

[cit13] Vogt C., Meirer F., Monai M., Groeneveld E., Ferri D., van Santen R. A., Nachtegaal M., Unocic R. R., Frenkel A. I., Weckhuysen B. M. (2021). Nat. Commun..

[cit14] Riscoe A. R., Wrasman C. J., Herzing A. A., Hoffman A. S., Menon A., Boubnov A., Vargas M., Bare S. R., Cargnello M. (2019). Nat. Catal..

[cit15] Lou Y., Xu J., Zhang Y., Pan C., Dong Y., Zhu Y. (2020). Mater. Today Nano.

[cit16] van Deelen T. W., Hernández Mejía C., de Jong K. P. (2019). Nat. Catal..

[cit17] Pacchioni G., Freund H.-J. (2018). Chem. Soc. Rev..

[cit18] Salvadori E., Bruzzese P. C., Giamello E., Chiesa M. (2022). Acc. Chem. Res..

[cit19] van Santen R. A., Tranca I., Hensen E. J. M. (2015). Catal. Today.

[cit20] Vogt C. (2023). J. Phys. Chem. C.

[cit21] Tauster S., Fung S., Garten R. L. (1978). J. Am. Chem. Soc..

[cit22] Campbell C. T. (2012). Nat. Chem..

[cit23] Bruix A., Rodriguez J. A., Ramírez P. J., Senanayake S. D., Evans J., Park J. B., Stacchiola D., Liu P., Hrbek J., Illas F. (2012). J. Am. Chem. Soc..

[cit24] Qian K., Duan H., Li Y., Huang W. (2020). Chem. - Eur. J..

[cit25] Qiao B., Liang J.-X., Wang A., Xu C.-Q., Li J., Zhang T., Liu J. J. (2015). Nano Res..

[cit26] Liu X., Liu M.-H., Luo Y.-C., Mou C.-Y., Lin S. D., Cheng H., Chen J.-M., Lee J.-F., Lin T.-S. (2012). J. Am. Chem. Soc..

[cit27] Li Y., Zhang Y., Qian K., Huang W. (2022). ACS Catal..

[cit28] Yang C., Pei C., Luo R., Liu S., Wang Y., Wang Z., Zhao Z.-J., Gong J. (2020). J. Am. Chem. Soc..

[cit29] Matsubu J. C., Zhang S., DeRita L., Marinkovic N. S., Chen J. G., Graham G. W., Pan X., Christopher P. (2017). Nat. Chem..

[cit30] Chandler B. D. (2017). Nat. Chem..

[cit31] Zhang J., Wang H., Wang L., Ali S., Wang C., Wang L., Meng X., Li B., Su D. S., Xiao F.-S. (2019). J. Am. Chem. Soc..

[cit32] Lykhach Y., Kozlov S. M., Skála T., Tovt A., Stetsovych V., Tsud N., Dvořák F., Johánek V., Neitzel A., Mysliveček J. (2016). Nat. Mater..

[cit33] Schwab G.-M. , in Advances in Catalysis, ed. D. D. Eley, H. Pines and P. B. Weisz, Academic Press, New York, 1979, vol. 27, pp. 1–22

[cit34] Lang R., Du X., Huang Y., Jiang X., Zhang Q., Guo Y., Liu K., Qiao B., Wang A., Zhang T. (2020). Chem. Rev..

[cit35] Sui X., Zhang L., Li J., Doyle-Davis K., Li R., Wang Z., Sun X. (2022). Adv. Energy Mater..

[cit36] Finzel J., Sanroman Gutierrez K. M., Hoffman A. S., Resasco J., Christopher P., Bare S. R. (2023). ACS Catal..

[cit37] Luo Z., Zhao G., Pan H., Sun W. (2022). Adv. Energy Mater..

[cit38] Cheng Q., Liu Y., Lyu S., Tian Y., Ma Q., Li X. (2021). Chin. J. Chem. Eng..

[cit39] Du X., Tang H., Qiao B. (2021). Catalysts.

[cit40] Pacchioni G. (2013). Phys. Chem. Chem. Phys..

[cit41] Tauster S., Fung S., Baker R., Horsley J. (1981). Science.

[cit42] Tauster S. (1987). Acc. Chem. Res..

[cit43] Haller G. L., Resasco D. E. (1989). Adv. Catal..

[cit44] Bernal S., Calvino J. J., Cauqui M. A., Gatica J. M., López Cartes C., Pérez Omil J. A., Pintado J. M. (2003). Catal. Today.

[cit45] Ahmadi M., Mistry H., Roldan Cuenya B. (2016). J. Phys. Chem. Lett..

[cit46] Pan C.-J., Tsai M.-C., Su W.-N., Rick J., Akalework N. G., Agegnehu A. K., Cheng S.-Y., Hwang B.-J. (2017). J. Taiwan Inst. Chem. Eng..

[cit47] Liu J. (2011). ChemCatChem.

[cit48] Pu T., Zhang W., Zhu M. (2023). Angew. Chem., Int. Ed..

[cit49] Xie Y., Wen J., Li Z., Chen J., Zhang Q., Ning P., Hao J. (2023). ACS Mater. Lett..

[cit50] Burch R., Flambard A. (1982). J. Catal..

[cit51] Den Otter G., Dautzenberg F. M. (1978). J. Catal..

[cit52] Schwab G.-M., Schlütes H. (1930). Z. Phys. Chem..

[cit53] Uzun A., Bhirud V. A., Kletnieks P. W., Haw J. F., Gates B. C. (2007). J. Phys. Chem. C.

[cit54] Tauster S., Fung S. (1978). J. Catal..

[cit55] Chen B. H., White J. (1982). J. Phys. Chem..

[cit56] Huizinga T. and Prins R., in Studies in Surface Science and Catalysis, ed. B. Imelik, C. Naccache, G. Coudurier, H. Praliaud, P. Meriaudeau, P. Gallezot, G. A. Martin, J. C. Vedrine, Elsevier, 1982, vol. 11, pp. 11–17

[cit57] Ponec V. , in Studies in Surface Science and Catalysis, ed. B. Imelik, C. Naccache, G. Coudurier, H. Praliaud, P. Meriaudeau, P. Gallezot, G. A. Martin, J. C. Vedrine, Elsevier, 1982, vol. 11, pp. 63–75

[cit58] Resasco D. E., Haller G. (1983). J. Catal..

[cit59] Braunschweig E. J., Logan A. D., Datye A. K., Smith D. J. (1989). J. Catal..

[cit60] Román-Martínez M., Cazorla-Amorós D., Linares-Solano A., Salinas-Martí'nez De Lecea C., Yamashita H., Anpo M. (1995). Carbon.

[cit61] Fu Q., Wagner T., Olliges S., Carstanjen H.-D. (2005). J. Phys. Chem. B.

[cit62] Ioannides T., Verykios X. E. (1996). J. Catal..

[cit63] Horsley J. (1979). J. Am. Chem. Soc..

[cit64] Chung Y.-W., Xiong G., Kao C.-C. (1984). J. Catal..

[cit65] Meriaudeau P., Ellestad O., Dufaux M., Naccache C. (1982). J. Catal..

[cit66] Wang S. Y., Moon S., Vannice M. A. (1981). J. Catal..

[cit67] Vannice M., Garten R. (1979). J. Catal..

[cit68] Liu S., Xu W., Niu Y., Zhang B., Zheng L., Liu W., Li L., Wang J. (2019). Nat. Commun..

[cit69] Liu S., Qi H., Zhou J., Xu W., Niu Y., Zhang B., Zhao Y., Liu W., Ao Z., Kuang Z., Li L., Wang M., Wang J. (2021). ACS Catal..

[cit70] Fu Q., Wagner T. (2007). Surf. Sci. Rep..

[cit71] Semiconductor Physics and Devices: Basic Principles, ed. D. A. Neamen, McGraw-Hill, Boston, 3rd edn, 2003

[cit72] Mine S., Ting K. W., Li L., Hinuma Y., Maeno Z., Siddiki S. H., Toyao T., Shimizu K.-I. (2022). J. Phys. Chem. C.

[cit73] CRC Handbook of Chemistry and Physics, ed. D. R. Lide, CRC Press/Taylor & Francis Group, Boca Raton, 90th edn, 2010

[cit74] Ruban A., Hammer B., Stoltze P., Skriver H. L., Nørskov J. K. (1997). J. Mol. Catal. A: Chem..

[cit75] Hannay N. B., Smyth C. P. (1946). J. Am. Chem. Soc..

[cit76] Tung R. T. (2000). Phys. Rev. Lett..

[cit77] Hammer B., Nørskov J. K. (1995). Nature.

[cit78] Sang K., Zuo J., Zhang X., Wang Q., Chen W., Qian G., Duan X. (2023). Green Energy Environ..

[cit79] Zou H., Guo L., Xue H., Zhang Y., Shen X., Liu X., Wang P., He X., Dai G., Jiang P., Zheng H., Zhang B., Xu C., Wang Z. L. (2020). Nat. Commun..

[cit80] Zhang Z., Yates, Jr. J. T. (2012). Chem. Rev..

[cit81] Huebener R. P. , in Conductors, Semiconductors, Superconductors: An Introduction to Solid State Physics, ed. R. P. Huebener, Springer International Publishing, 2015, pp. 185–198

[cit82] Tung R. T. (2001). Mater. Sci. Eng. R Rep..

[cit83] Lyons J. L. , Janotti A. and Van de Walle C. G., Semiconductors and semimetals, Elsevier, 2013, vol. 88, pp. 1–37

[cit84] Lüth H. , Solid surfaces, interfaces and thin films, Springer, Cham, 2015

[cit85] Campbell C. T., Mao Z. (2017). ACS Catal..

[cit86] Mao Z., Campbell C. T. (2021). ACS Catal..

[cit87] Campbell C. T., Sellers J. R. (2013). Faraday Discuss..

[cit88] Nørskov J. K., Bligaard T., Rossmeisl J., Christensen C. H. (2009). Nat. Chem..

[cit89] Noronha F. B., Fendley E. C., Soares R. R., Alvarez W. E., Resasco D. E. (2001). Chem. Eng. J..

[cit90] Hemmingson S. L., Campbell C. T. (2017). ACS Nano.

[cit91] Goodman E. D., Schwalbe J. A., Cargnello M. (2017). ACS Catal..

[cit92] O’Connor N. J., Jonayat A., Janik M. J., Senftle T. P. (2018). Nat. Catal..

[cit93] Iyemperumal S. K., Pham T. D., Bauer J., Deskins N. A. (2018). J. Phys. Chem. C.

[cit94] Dietze E. M., Plessow P. N. (2019). J. Phys. Chem. C.

[cit95] Su Y.-Q., Zhang L., Wang Y., Liu J.-X., Muravev V., Alexopoulos K., Filot I. A., Vlachos D. G., Hensen E. J. (2020). Npj Comput. Mater..

[cit96] Beck A., Huang X., Artiglia L., Zabilskiy M., Wang X., Rzepka P., Palagin D., Willinger M.-G., van Bokhoven J. A. (2020). Nat. Commun..

[cit97] Gates B. C., Flytzani-Stephanopoulos M., Dixon D. A., Katz A. (2017). Catal. Sci. Technol..

[cit98] Plessow P. N., Bajdich M., Greene J., Vojvodic A., Abild-Pedersen F. (2016). J. Phys. Chem. C.

[cit99] Wang X., Beck A., van Bokhoven J. A., Palagin D. (2021). J. Mater. Chem A.

[cit100] Wulff G. (1901). Cryst. Mater..

[cit101] Winterbottom W. L. (1967). Acta Metall..

[cit102] Chen P., Gao Y., Castell M. R. (2021). Phys. Rev. Mater..

[cit103] Labich S., Taglauer E., Knözinger H. (2000). Top. Catal..

[cit104] Handbook of heterogeneous catalysis, ed. G. Ertl, H. Knözinger and J. Weitkamp, Wiley-VCH, Weinheim, 2nd edn, 2008

[cit105] Gao Y., Liang Y., Chambers S. A. (1996). Surf. Sci..

[cit106] Berkó A., Ulrych I., Prince K. (1998). J. Phys. Chem. B.

[cit107] Pesty F., Steinrück H.-P., Madey T. E. (1995). Surf. Sci..

[cit108] Mezey L. Z., Giber J. (1982). Jpn. J. Appl. Phys..

[cit109] Deng L., Miura H., Shishido T., Hosokawa S., Teramura K., Tanaka T. (2017). Chem. Commun..

[cit110] Wang L., Zhang L., Zhang L., Yun Y., Wang K., Yu B., Zhao X., Yang F. (2023). Nano Res..

[cit111] Wang J., Lu A.-H., Li M., Zhang W., Chen Y.-S., Tian D.-X., Li W.-C. (2013). ACS Nano.

[cit112] Yang F., Zhao H., Wang W., Wang L., Zhang L., Liu T., Sheng J., Zhu S., He D., Lin L., He J., Wang R., Li Y. (2021). Chem. Sci..

[cit113] Beck A. M., Frey H., Huang X., Clark A. H., Goodman E. D., Cargnello M., Willinger M. G., van Bokhoven J. A. (2023). Angew. Chem., Int. Ed..

[cit114] Macino M., Barnes A. J., Althahban S. M., Qu R., Gibson E. K., Morgan D. J., Freakley S. J., Dimitratos N., Kiely C. J., Gao X. (2019). Nat. Catal..

[cit115] Wu Z., Li Y., Huang W. (2020). J. Phys. Chem. Lett..

[cit116] Du X., Huang Y., Pan X., Han B., Su Y., Jiang Q., Li M., Tang H., Li G., Qiao B. (2020). Nat. Commun..

[cit117] Wan Z., Wang Q.-D., Liu D., Liang J. (2021). Phys. Chem. Chem. Phys..

[cit118] Hinuma Y., Toyao T., Kamachi T., Maeno Z., Takakusagi S., Furukawa S., Takigawa I., Shimizu K.-I. (2018). J. Phys. Chem. C.

[cit119] Deml A. M., Holder A. M., O’Hayre R. P., Musgrave C. B., Stevanovic V. (2015). J. Phys. Chem. Lett..

[cit120] Ruiz Puigdollers A., Schlexer P., Tosoni S., Pacchioni G. (2017). ACS Catal..

[cit121] Duffy J. A. (2006). J. Phys. Chem. A.

[cit122] Shaw P. E. (1926). Nature.

[cit123] Maynes A. J., Driscoll D. M., DeSario P. A., Pietron J. J., Pennington A. M., Rolison D. R., Morris J. R. (2020). J. Phys. Chem. C.

[cit124] Ament K., Köwitsch N., Hou D., Götsch T., Kröhnert J., Heard C. J., Trunschke A., Lunkenbein T., Armbrüster M., Breu J. (2021). Angew. Chem., Int. Ed..

[cit125] Lyu S., Zhang Y., Li Z., Liu X., Tian Z., Liu C., Li J., Wang L. (2022). Front. Chem..

[cit126] Yang W., Li J., Cui X., Yang C., Liu Y., Zeng X., Zhang Z., Zhang Q. (2021). Chin. Chem. Lett..

[cit127] Rodriguez J. A., Liu P., Graciani J., Senanayake S. D., Grinter D. C., Stacchiola D., Hrbek J., Fernández-Sanz J. (2016). J. Phys. Chem. Lett..

[cit128] Liu X., Luo J., Wang H., Huang L., Wang S., Li S., Sun Z., Sun F., Jiang Z., Wei S., Li W.-X., Lu J. (2022). Angew. Chem., Int. Ed..

[cit129] Senanayake S. D., Ramírez P. J., Waluyo I., Kundu S., Mudiyanselage K., Liu Z., Liu Z., Axnanda S., Stacchiola D. J., Evans J. (2016). J. Phys. Chem. C.

[cit130] Gao Q., Li W., Liu P., Wang Q., Yang Y. (2023). Appl. Surf. Sci..

[cit131] Graciani J. s, Vidal A. B., Rodriguez J. A., Sanz J. F. (2014). J. Phys. Chem. C.

[cit132] Yang J., Huang Y., Qi H., Zeng C., Jiang Q., Cui Y., Su Y., Du X., Pan X., Liu X. (2022). Nat. Commun..

[cit133] Qi K., Chhowalla M., Voiry D. (2020). Mater. Today.

[cit134] Giulimondi V., Mitchell S., Pérez-Ramírez J. (2023). ACS Catal..

[cit135] Yang X.-F., Wang A., Qiao B., Li J., Liu J., Zhang T. (2013). Acc. Chem. Res..

[cit136] Han B., Guo Y., Huang Y., Xi W., Xu J., Luo J., Qi H., Ren Y., Liu X., Qiao B. (2020). Angew. Chem., Int. Ed..

[cit137] Xu J., Xu H., Dong A., Zhang H., Zhou Y., Dong H., Tang B., Liu Y., Zhang L., Liu X. (2022). Adv. Mater..

[cit138] Ishida T., Murayama T., Taketoshi A., Haruta M. (2019). Chem. Rev..

[cit139] Beniya A., Higashi S. (2019). Nat. Catal..

[cit140] Beniya A., Higashi S., Ohba N., Jinnouchi R., Hirata H., Watanabe Y. (2020). Nat. Commun..

[cit141] Zhang S., Plessow P. N., Willis J. J., Dai S., Xu M., Graham G. W., Cargnello M., Abild-Pedersen F., Pan X. (2016). Nano Lett..

[cit142] Tang H., Su Y., Zhang B., Lee A. F., Isaacs M. A., Wilson K., Li L., Ren Y., Huang J., Haruta M. (2017). Sci. Adv..

[cit143] Penner S., Armbrüster M. (2015). ChemCatChem.

[cit144] He L., Ren Y., Yue B., Tsang S. C. E., He H. (2021). Processes.

[cit145] Praliaud H., Martin G. (1981). J. Catal..

[cit146] Labich S., Kohl A., Taglauer E., Knözinger H. (1998). J. Chem. Phys..

[cit147] Lamber R., Jaeger N. I. (1991). J. Appl. Phys..

[cit148] Lamber R. , Jaeger N. and Schulz-Ekloff G., in Studies in Surface Science and Catalysis, ed. C. Morterra, A. Zecchina, G. Costa, Elsevier, Amsterdam, 1989, vol. 48, pp. 559–565

[cit149] Ren-Yuam T., Rong-An W., Li-Wu L. (1984). Appl. Catal..

[cit150] Zabilskiy M., Sushkevich V. L., Palagin D., Newton M. A., Krumeich F., van Bokhoven J. A. (2020). Nat. Commun..

[cit151] Zhang P., Lu H., Zhou Y., Zhang L., Wu Z., Yang S., Shi H., Zhu Q., Chen Y., Dai S. (2015). Nat. Commun..

[cit152] Yuan S., Yang Y., Xiong Z., Guo P., Sun S., Li Z., Du J., Gao Y. (2024). Green Energy Environ..

[cit153] Holse C., Elkjær C. F., Nierhoff A., Sehested J., Chorkendorff I., Helveg S., Nielsen J. H. (2015). J. Phys. Chem. C.

[cit154] Zhao W., Zhou D., Han S., Li Y., Liu J., Zhou Y., Li M., Zhang X., Shen W. (2021). J. Phys. Chem. C.

[cit155] Chun W.-J., Ishikawa A., Fujisawa H., Takata T., Kondo J. N., Hara M., Kawai M., Matsumoto Y., Domen K. (2003). J. Phys. Chem. B.

[cit156] Spencer M. (1985). J. Catal..

[cit157] Hankin A., Bedoya-Lora F. E., Alexander J. C., Regoutz A., Kelsall G. H. (2019). J. Mater. Chem. A.

[cit158] Xu Y., Schoonen M. A. (2000). Am. Mineral..

[cit159] Wu P., Tan S., Moon J., Yan Z., Fung V., Li N., Yang S.-Z., Cheng Y., Abney C. W., Wu Z. (2020). Nat. Commun..

[cit160] Schilling C., Hess C. (2018). J. Phys. Chem. C.

[cit161] Biswas S., Pal A., Pal T. (2020). RSC Adv..

[cit162] Yuan X., Pu T., Gu M., Zhu M., Xu J. (2021). ACS Catal..

[cit163] Chen H.-W., White J., Ekerdt J. (1986). J. Catal..

[cit164] Ha H., Yoon S., An K., Kim H. Y. (2018). ACS Catal..

[cit165] Ren J., Mebrahtu C., Palkovits R. (2020). Catal. Sci. Technol..

[cit166] Ma Z., Zhao S., Pei X., Xiong X., Hu B. (2017). Catal. Sci. Technol..

[cit167] Belgamwar R., Verma R., Das T., Chakraborty S., Sarawade P., Polshettiwar V. (2023). J. Am. Chem. Soc..

[cit168] Greiner M. T., Chai L., Helander M. G., Tang W. M., Lu Z. H. (2012). Adv. Funct. Mater..

[cit169] Chen S., Abdel-Mageed A. M., Li M., Cisneros S., Bansmann J., Rabeah J., Brückner A., Groß A., Behm R. J. (2021). J. Catal..

[cit170] Mohan O., Shambhawi S., Xu R., Lapkin A. A., Mushrif S. H. (2021). ChemCatChem.

[cit171] Long F., Wu S., Chen H., Jia S., Cao X., Liu P., Lu Y., Jiang J., Zhang X., Xu J. (2023). J. Catal..

[cit172] Yuan M., Zhong S., Li G., Fan G., Yu X. (2024). Fuel.

[cit173] Parastaev A., Muravev V., Huertas Osta E., van Hoof A. J., Kimpel T. F., Kosinov N., Hensen E. J. (2020). Nat. Catal..

[cit174] Shah S., Hong J., Cruz L., Wasantwisut S., Bare S. R., Gilliard-AbdulAziz K. L. (2023). ACS Catal..

[cit175] Naeem M. A., Burueva D. B., Abdala P. M., Bushkov N. S., Stoian D., Bukhtiyarov A. V., Prosvirin I. P., Bukhtiyarov V. I., Kovtunov K. V., Koptyug I. V. (2020). J. Phys. Chem. C.

[cit176] Chen S., Abdel-Mageed A. M., Dyballa M., Parlinska-Wojtan M., Bansmann J., Pollastri S., Olivi L., Aquilanti G., Behm R. J. (2020). Angew. Chem. Int. Ed..

[cit177] Jenness G. R., Schmidt J. R. (2013). ACS Catal..

[cit178] Lany S., Osorio-Guillén J., Zunger A. (2007). Phys. Rev. B: Condens. Matter Mater. Phys..

[cit179] He H. , in Solution Processed Metal Oxide Thin Films for Electronic Applications, ed. Z. Cui, Elsevier, Amsterdam, 1st edn, 2020, pp. 7–30

[cit180] Sato H., Minami T., Takata S., Yamada T. (1993). Thin Solid Films.

[cit181] Solymosi F. (1968). Catal. Rev..

[cit182] Akubuiro E. C., Verykios X. E. (1987). J. Catal..

[cit183] Kim J.-H., Kwon G., Lim H., Zhu C., You H., Kim Y.-T. (2016). J. Power Sources.

[cit184] Zhang Z., Kladi A., Verykios X. E. (1994). J. Catal..

[cit185] Chen L., Kovarik L., Meira D., Szanyi J. (2022). ACS Catal..

[cit186] Chen P., Khetan A., Yang F., Migunov V., Weide P., Stürmer S. P., Guo P., Kähler K., Xia W., Mayer J. (2017). ACS Catal..

[cit187] Yarulin A., Berguerand C., Alonso A. O., Yuranov I., Kiwi-Minsker L. (2015). Catal. Today.

[cit188] Sun Q., Zhang H., Fan Y., Bian L., Peng Q., Liu B. (2023). Renew. Energy.

[cit189] Hu S., Li W.-X. (2021). Science.

[cit190] Zhang J., Qin X., Chu X., Chen M., Chen X., Chen J., He H., Zhang C. (2021). Environ. Sci. Technol..

[cit191] Chen H., Yang Z., Wang X., Polo-Garzon F., Halstenberg P. W., Wang T., Suo X., Yang S.-Z., Meyer III H. M., Wu Z. (2021). J. Am. Chem. Soc..

[cit192] Kim M.-S., Chung S.-H., Yoo C.-J., Lee M. S., Cho I.-H., Lee D.-W., Lee K.-Y. (2013). Appl. Catal., B.

[cit193] Li C., Shi Y., Zhang Z., Ni J., Wang X., Lin J., Lin B., Jiang L. (2021). J. Energy Chem..

[cit194] d’Alnoncourt R. N., Xia X., Strunk J., Löffler E., Hinrichsen O., Muhler M. (2006). Phys. Chem. Chem. Phys..

[cit195] Zhang C., Wang L., Etim U. J., Song Y., Gazit O. M., Zhong Z. (2022). J. Catal..

[cit196] Tang H., Su Y., Guo Y., Zhang L., Li T., Zang K., Liu F., Li L., Luo J., Qiao B., Wang J. (2018). Chem. Sci..

[cit197] Frey H., Beck A., Huang X., van Bokhoven J. A., Willinger M.-G. (2022). Science.

[cit198] Hernández Mejía C., van Deelen T. W., de Jong K. P. (2018). Nat. Commun..

[cit199] Wang H., Wang L., Lin D., Feng X., Niu Y., Zhang B., Xiao F.-S. (2021). Nat. Catal..

[cit200] Zhang J., Zhu D., Yan J., Wang C.-A. (2021). Nat. Commun..

[cit201] Wan G., Zhang G., Chen J. Z., Toney M. F., Miller J. T., Tassone C. J. (2022). ACS Catal..

[cit202] Xin H., Lin L., Li R., Li D., Song T., Mu R., Fu Q., Bao X. (2022). J. Am. Chem. Soc..

[cit203] Polo-Garzon F., Blum T. F., Bao Z., Wang K., Fung V., Huang Z., Bickel E. E., Jiang D.-E., Chi M., Wu Z. (2021). ACS Catal..

[cit204] Monai M., Jenkinson K., Melcherts A. E., Louwen J. N., Irmak E. A., Van Aert S., Altantzis T., Vogt C., van der Stam W., Duchoň T. (2023). Science.

[cit205] Zhang J., Ma J., Choksi T. S., Zhou D., Han S., Liao Y.-F., Yang H. B., Liu D., Zeng Z., Liu W. (2022). J. Am. Chem. Soc..

[cit206] Singh J. A., Hoffman A. S., Schumann J., Boubnov A., Asundi A. S., Nathan S. S., Nørskov J., Bare S. R., Bent S. F. (2019). ChemCatChem.

[cit207] Fernandez A., Gonzalez-Elipe A., Caballero A., Munuera G. (1993). J. Phys. Chem..

[cit208] Ohyama J., Yamamoto A., Teramura K., Shishido T., Tanaka T. (2011). ACS Catal..

[cit209] Zhang J., Zhu D., Yan J., Wang C.-A. (2021). Nat. Commun..

[cit210] Ranney J., Starr D., Musgrove J., Bald D., Campbell C. (1999). Faraday Discuss..

[cit211] Zhao K., Auerbach D. J., Campbell C. T. (2023). ACS Catal..

[cit212] Jensen M. C. R., Venkataramani K., Helveg S., Clausen B., Reichling M., Besenbacher F., Lauritsen J. V. (2008). J. Phys. Chem. C.

[cit213] Worren T., Hansen K. H., Lægsgaard E., Besenbacher F., Stensgaard I. (2001). Surf. Sci..

[cit214] Giorgio S., Cabie M., Henry C. (2008). Gold Bull..

[cit215] Hansen P. L., Wagner J. B., Helveg S., Rostrup-Nielsen J. R., Clausen B. S., Topsøe H. (2002). Science.

[cit216] Zhang L., Cosandey F., Persaud R., Madey T. E. (1999). Surf. Sci..

[cit217] Hachtel J. A., Idrobo J. C., Chi M. (2018). Adv. Struct. Chem. Imag..

[cit218] Ophus C. (2019). Microsc. Microanal..

[cit219] Lazić I., Bosch E. G., Lazar S. (2016). Ultramicroscopy.

[cit220] Zachman M. J., Fung V., Polo-Garzon F., Cao S., Moon J., Huang Z., Jiang D.-E., Wu Z., Chi M. (2022). Nat. Commun..

[cit221] Seki T., Ikuhara Y., Shibata N. (2021). Microscopy.

[cit222] Jackson C., Smith G. T., Inwood D. W., Leach A. S., Whalley P. S., Callisti M., Polcar T., Russell A. E., Levecque P., Kramer D. (2017). Nat. Commun..

[cit223] Timoshenko J., Roldan Cuenya B. (2021). Chem. Rev..

[cit224] Behafarid F., Ono L. K., Mostafa S., Croy J. R., Shafai G., Hong S., Rahman T. S., Bare S. R., Roldan Cuenya B. (2012). Phys. Chem. Chem. Phys..

[cit225] Yu J., Sun X., Tong X., Zhang J., Li J., Li S., Liu Y., Tsubaki N., Abe T., Sun J. (2021). Nat. Commun..

[cit226] Klein J., Kampermann L., Mockenhaupt B., Behrens M., Strunk J., Bacher G. (2023). Adv. Funct. Mater..

[cit227] Zanatta A. R. (2019). Sci. Rep..

[cit228] Miyazaki S., Narasaki M., Ogasawara M., Hirose M. (2002). Solid-State Electron..

[cit229] Ohta A., Murakami H., Makihara K., Miyazaki S. (2015). Jpn. J. Appl. Phys..

[cit230] Miyazaki S., Nishimura H., Fukuda M., Ley L., Ristein J. (1997). Appl. Surf. Sci..

[cit231] Helander M., Greiner M., Wang Z., Lu Z. (2010). Appl. Surf. Sci..

[cit232] Melitz W., Shen J., Kummel A. C., Lee S. (2011). Surf. Sci. Rep..

[cit233] Yoshida H. (2015). J. Electron Spectrosc. Relat. Phenom..

[cit234] Krause S., Schöll A., Umbach E. (2015). Phys. Rev. B: Condens. Matter Mater. Phys..

[cit235] Yoshida H. (2012). Chem. Phys. Lett..

[cit236] Lee S. F., Jimenez-Relinque E., Martinez I., Castellote M. (2023). Catalysts.

[cit237] Muñoz M., Farges F., Argoul P. (2005). Phys. Scr..

[cit238] Weckhuysen B. M. , Wondergem C. S. and Vogt C. in Handbook of Advanced Catalyst Characterization, ed. I. E. Wachs, M. Bañares, Springer International Publishing, Cham, 2023, 1st edn, pp. 601–623

[cit239] Wu Z., Li M., Howe J., Meyer, III H. M., Overbury S. H. (2010). Langmuir.

[cit240] Du X., Liu D., An K., Jiang S., Wei Z., Wang S., Ip W. F., Pan H. (2022). Appl. Mater. Today.

[cit241] Yoshitake H., Iwasawa Y. (1992). J. Phys. Chem..

[cit242] Schumann J., Kröhnert J., Frei E., Schlögl R., Trunschke A. (2017). Top. Catal..

[cit243] Baker R., Prestridge E., Garten R. (1979). J. Catal..

[cit244] Baker R., Prestridge E., Garten R. (1979). J. Catal..

[cit245] Yang F., Zhao H., Wang W., Wang L., Zhang L., Liu T., Sheng J., Zhu S., He D., Lin L. (2021). Chem. Sci..

[cit246] Petzoldt P., Eder M., Mackewicz S., Blum M., Kratky T., Günther S., Tschurl M., Heiz U., Lechner B. A. (2022). J. Phys. Chem. C.

[cit247] Martín A. J., Mitchell S., Mondelli C., Jaydev S., Pérez-Ramírez J. (2022). Nat. Catal..

[cit248] Qiu C., Odarchenko Y., Meng Q., Cong P., Schoen M. A., Kleibert A., Forrest T., Beale A. M. (2020). Chem. Sci..

[cit249] Matos J., Ono L., Behafarid F., Croy J., Mostafa S., DeLaRiva A., Datye A., Frenkel A., Cuenya B. R. (2012). Phys. Chem. Chem. Phys..

[cit250] Kim G. J., Kwon D. W., Hong S. C. (2016). J. Phys. Chem. C.

[cit251] Pu Y., Niu Y., Wang Y., Liang Q., Zhang L., Zhang B. (2021). J. Phys. Chem. C.

[cit252] Jiao W.-Z., Yin P., Tong L., Xu S.-L., Ma C.-S., Zuo L.-J., Wang A., Liang H.-W. (2022). Inorg. Chem..

[cit253] Kwak J. H., Hu J., Mei D., Yi C.-W., Kim D. H., Peden C. H., Allard L. F., Szanyi J. (2009). Science.

[cit254] Mei D., Kwak J. H., Hu J., Cho S. J., Szanyi J., Allard L. F., Peden C. H. (2010). J. Phys. Chem. Lett..

[cit255] Hernández Mejía C., Vogt C., Weckhuysen B. M., de Jong K. P. (2020). Catal. Today.

[cit256] Schumann J., Eichelbaum M., Lunkenbein T., Thomas N., Alvarez Galvan M. C., Schlögl R., Behrens M. (2015). ACS Catal..

[cit257] Wang Y., Widmann D., Behm R. J. (2017). ACS Catal..

[cit258] Zhu J., Su Y., Chai J., Muravev V., Kosinov N., Hensen E. J. (2020). ACS Catal..

[cit259] Chen S., Li S., You R., Guo Z., Wang F., Li G., Yuan W., Zhu B., Gao Y., Zhang Z. (2021). ACS Catal..

[cit260] Singh U. K., Vannice M. A. (2000). J. Mol. Catal. A: Chem..

[cit261] Reyes P., Rojas H., Pecchi G., Fierro J. (2002). J. Mol. Catal. A: Chem..

[cit262] Jiang F., Wang S., Liu B., Liu J., Wang L., Xiao Y., Xu Y., Liu X. (2020). ACS Catal..

[cit263] Abdel-Mageed A. M., Wiese K., Parlinska-Wojtan M., Rabeah J., Brückner A., Behm R. J. (2020). Appl. Catal., B.

[cit264] Elnabawy A. O., Schimmenti R., Cao A., Nørskov J. K. (2022). ACS Sustainable Chem. Eng..

[cit265] Vogt C., Monai M., Sterk E. B., Palle J., Melcherts A. E. M., Zijlstra B., Groeneveld E., Berben P. H., Boereboom J. M., Hensen E. J. M., Meirer F., Filot I. A. W., Weckhuysen B. M. (2019). Nat. Commun..

[cit266] Chen S., Xu Y., Chang X., Pan Y., Sun G., Wang X., Fu D., Pei C., Zhao Z.-J., Su D. (2024). Science.

[cit267] Bonati L., Polino D., Pizzolitto C., Biasi P., Eckert R., Reitmeier S., Schlögl R., Parrinello M. (2023). Proc. Natl. Acad. Sci. U. S. A..

