# Supplementary material for: Metal–support interactions in metal oxide-supported atomic, cluster, and nanoparticle catalysis
Source: Chem Soc Rev. 2024 Oct 2;53(21):10450–90. doi: 10.1039/d4cs00527a (PMC11445804; doi:10.1039/d4cs00527a)
Supplement: CS-053-D4CS00527A-s001 [file CS-053-D4CS00527A-s001.pdf]

Supplementary Information (SI) for Chemical Society Reviews.
This journal is © The Royal Society of Chemistry 2024

Supplementary information

Metal Support Interactions in Metal Oxide-Supported Atomic, Cluster, and Nanoparticle Catalysis

Denis Leybo[†1], Ubong J. Etim[†2], Matteo Monai[3], Simon R. Bare[*4,5], Ziyi Zhong[*2], Charlotte Vogt[*1]

[1] Schulich Faculty of Chemistry, and Resnick Sustainability Center for Catalysis, Technion, Israel Institute of Technology, Technion City, Haifa 32000, Israel
[2] Department of Chemical Engineering and Guangdong Provincial Key Laboratory of Materials and Technologies for Energy Conversion (MATEC), Guangdong Technion Israel Institute of Technology (GTIIT), 241 Daxue Road, Shantou, 515063, China
[3] Inorganic Chemistry and Catalysis group, Institute for Sustainable and Circular Chemistry, Utrecht University, Universiteitsweg 99, 3584 CG Utrecht, The Netherlands.
[4] Stanford Synchrotron Radiation Lightsource, SLAC National Accelerator Laboratory, 2575 Sand Hill Road, Menlo Park, CA 94025, USA
[5] SUNCAT Center for Interface Science and Catalysis, SLAC National Accelerator Laboratory, Menlo Park, CA 94025, USA

[*] Corresponding author
[†] These authors contributed equally to this work.

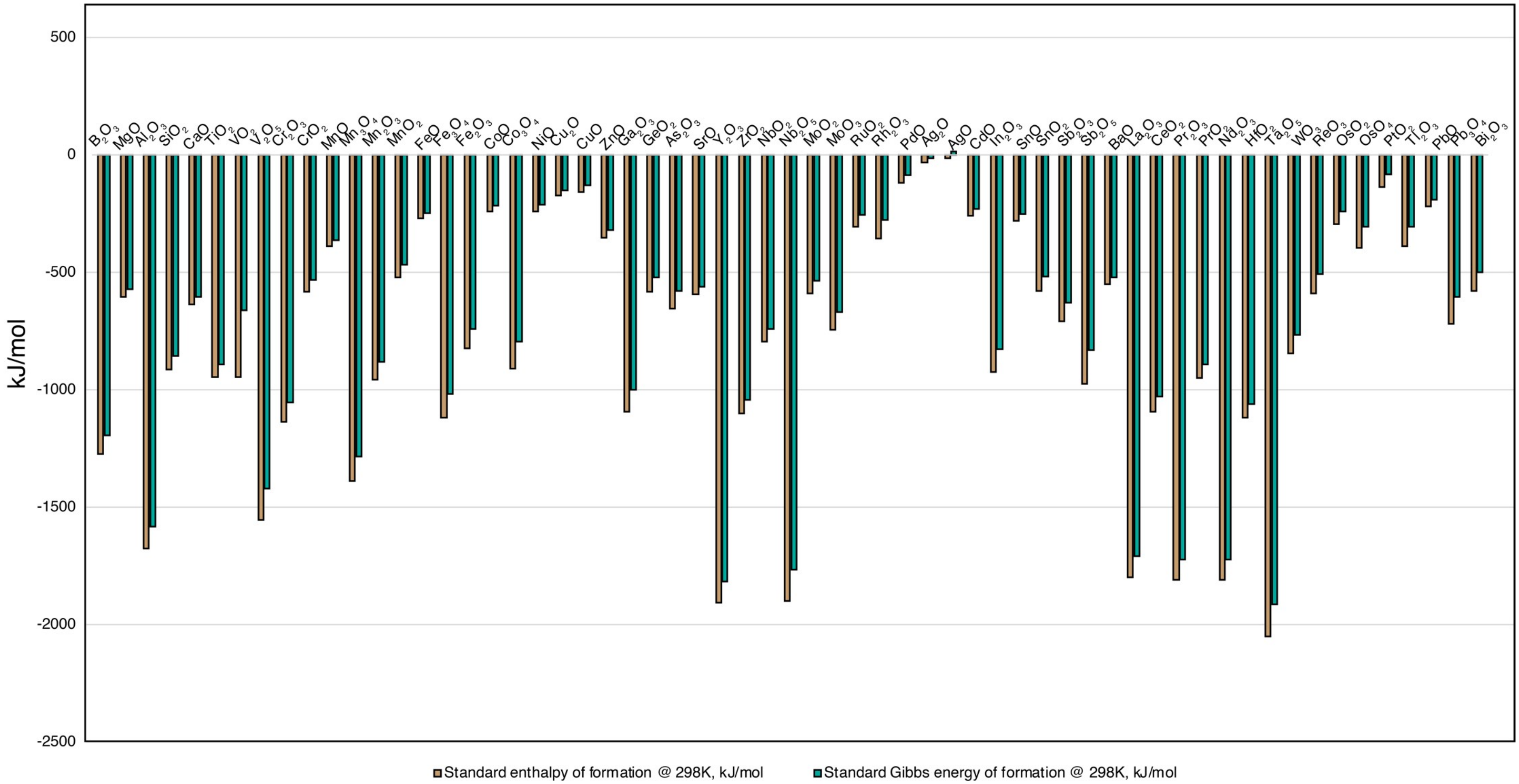

Figure S1. Standard enthalpy of formation and standard Gibbs energy of formation of selected binary oxides in kJ/mol.

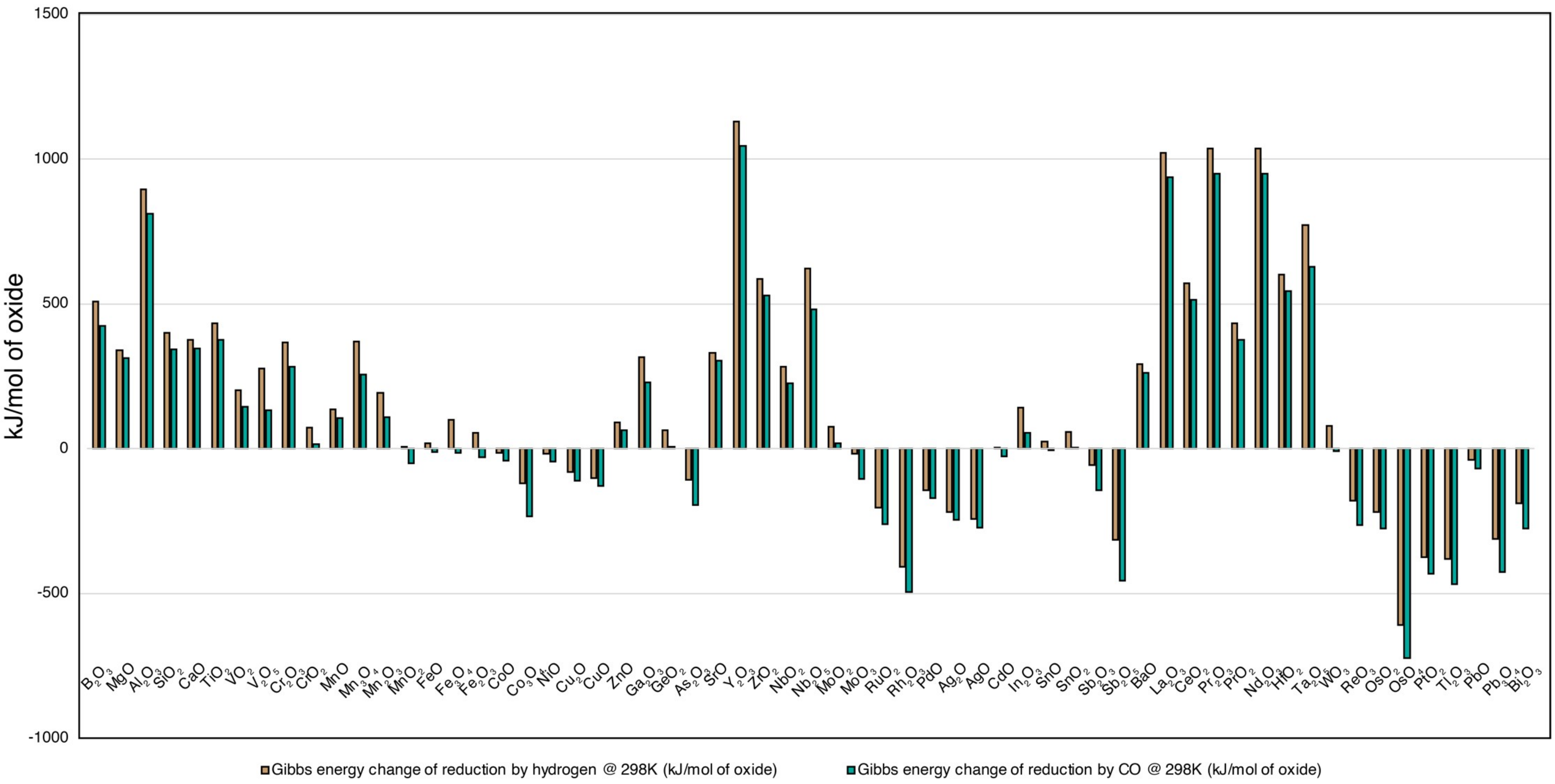

Figure S2. Hydrogen and CO reduction reaction Gibbs energy change in kJ/mol of oxide.

Table S1. Energies of formation for several metal oxides calculated using HSC 5.11 software, and enthalpy of oxygen formation data from literature, indicated in the last column.

Oxides	ΔH_f 298K (kJ/mol)	ΔG_f 298K (kJ/mol)	ΔG of reduction by H_2 298K (kJ/mol of oxide)	ΔG of reduction by CO @ 298K (kJ/mol of oxide)	Enthalpy of oxygen vacancy formation (eV/vac)	Ref
B_2O_3	-1273.5	-1194.325	508.58	422.773		
MgO	-601.6	-569.352	340.771	312.168	6	1
Al_2O_3	-1675.692	-1582.274	896.529	810.722	7	2
SiO_2	-910.857	-856.443	399.279	342.075	6.4	2
CaO	-634.92	-603.297	374.716	346.113	6.3	1
TiO_2	-944.747	-889.417	432.254	375.049	5.7	2
VO_2	-944.747	-658.637	201.474	144.269		
V_2O_5	-1551	-1418.576	275.667	132.656	4.7	1
Cr_2O_3	-1134.701	-1053.111	367.365	281.558	4.11	3
CrO_2	-581.576	-529.359	72.195	14.991	3	4
MnO	-385.221	-362.834	134.253	105.65		
Mn_3O_4	-1387.799	-1283.042	368.714	254.305	4.9	1
Mn_2O_3	-956.881	-878.853	193.108	107.301		
MnO_2	-520.029	-465.074	7.911	-49.294	4.5	1
FeO	-267.27	-245.724	17.143	-11.46	4.2	2
Fe_3O_4	-1118.383	-1015.226	100.899	-13.51		
Fe_2O_3	-823	-741.044	55.298	-30.509	4.38	5
CoO	-237.944	-214.198	-14.384	-42.986	4.1	2
Co_3O_4	-910.02	-794.901	-119.426	-233.835	4.2	1
NiO	-239.7	-211.585	-16.996	-45.599	3.8	1
Cu_2O	-170.6	-147.844	-80.738	-109.34	3.8	1
CuO	-155.8	-128.077	-100.505	-129.107	3.3	1
ZnO	-350.5	-320.374	91.792	63.19	4.2	1
Ga_2O_3	-1091	-1000.292	314.546	228.739	4.8	2
GeO_2	-579.902	-521.307	64.143	6.938	4.4	2
As_2O_3	-654.796	-576.651	-109.094	-194.901		
SrO	-591	-560.591	332.009	303.407	6.5	2
Y_2O_3	-1905	-1816.11	1130.365	1044.558	6.7	2
ZrO_2	-1100.3	-1042.477	585.313	528.109	6.8	2
NbO_2	-794.96	-739.235	282.072	224.867	5.6	1
Nb_2O_5	-1899.536	-1765.937	623.028	480.017		
MoO_2	-589.3	-533.487	76.323	19.119		
MoO_3	-744.6	-667.491	-18.254	-104.061	2.48	6
RuO_2	-305.014	-252.657	-204.507	-261.711		
Rh_2O_3	-355.64	-276.761	-408.984	-494.791	3.4	2
PdO	-115.478	-85.22	-143.362	-171.964		
Ag_2O	-31.13	-11.175	-217.406	-246.009		
AgO	-11.585	14.494	-243.076	-271.678		
CdO	-258.99	-229.305	0.723	-27.879	2.8	2
In_2O_3	-923	-827.227	141.482	55.675	3.7	2
SnO	-280.709	-251.912	23.33	-5.272	4.1	1
SnO_2	-577.631	-515.819	58.655	1.451	4	2
Sb_2O_3	-708.547	-628.384	-57.361	-143.168	3.45	6
Sb_2O_5	-971.901	-829.143	-313.766	-456.778		
BaO	-548	-520.25	291.668	263.066	5.9	2
La_2O_3	-1795.5	-1707.788	1022.042	936.236	6.69	6
CeO_2	-1090.4	-1027.102	569.938	512.734	2.87	6
Pr_2O_3	-1809.6	-1720.875	1035.129	949.323		
PrO_2	-949.35	-889.968	432.805	375.6		
Nd_2O_3	-1808.3	-1721.45	1035.705	949.898		
HfO_2	-1115.6	-1059.158	601.994	544.789	7	2

Ta_2O_5	-2049	-1913.645	770.736	627.725		
WO_3	-842.909	-764.065	78.319	-7.487	3.5	[7]
ReO_3	-589.107	-507.132	-178.614	-264.421		
OsO_2	-295	-239.644	-217.52	-274.725		
OsO_4	-394.099	-304.942	-609.385	-723.794		
PtO_2	-133.888	-80.894	-376.27	-433.474	2.9	[1]
Tl_2O_3	-387	-304.614	-381.131	-466.938		
PbO	-218.062	-188.641	-39.941	-68.543	3.76	[6]
Pb_3O_4	-718.686	-601.591	-312.736	-427.145		
Bi_2O_3	-578.01	-497.097	-188.648	-274.455	3.12	[6]

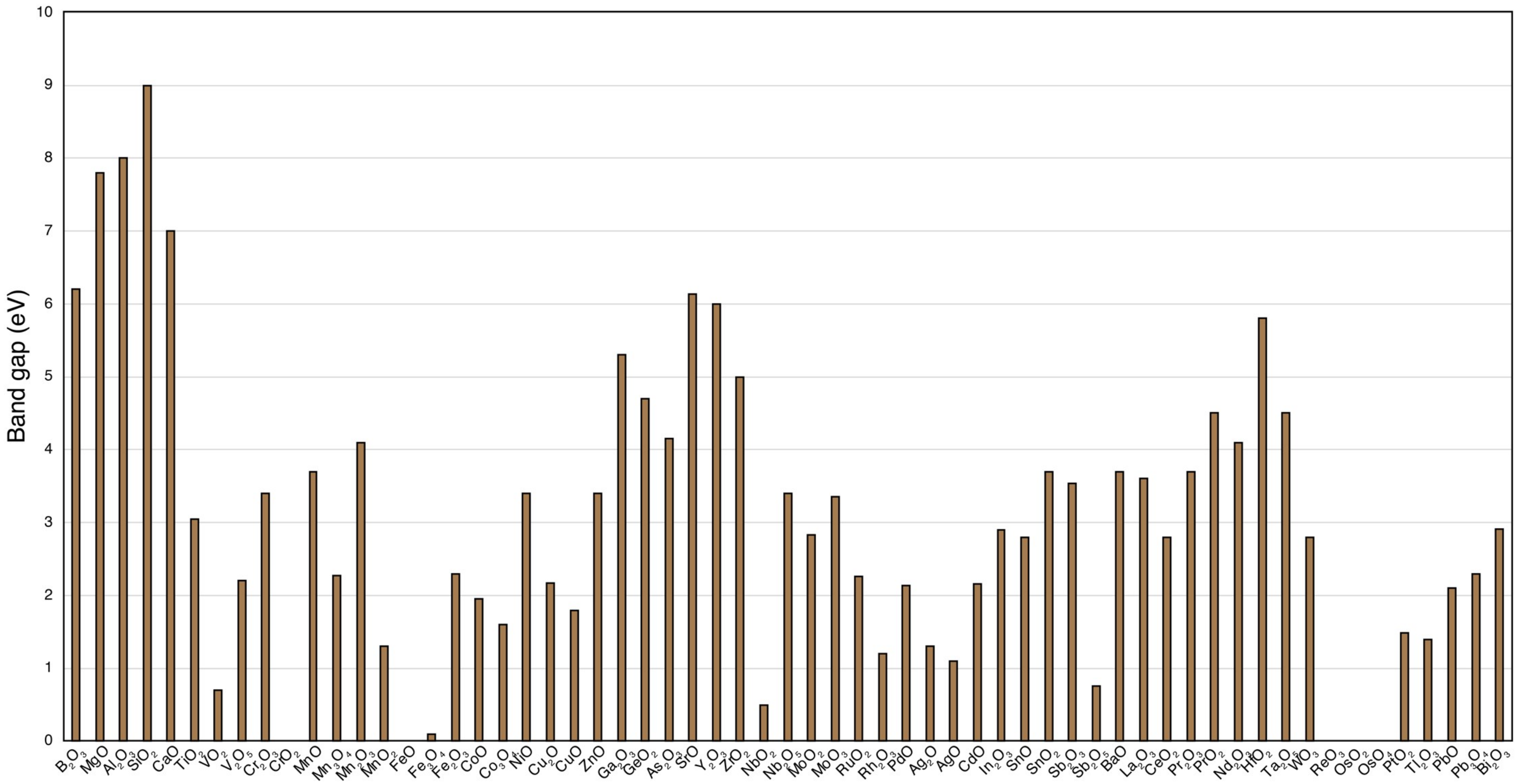

Figure S3. Bandgap values for selected binary metal oxides in eV.

Table S2. Reported band gaps for several metal oxides.

Oxides	Band Gap (eV)	Ref
B_2O_3	6.2	8
MgO	7.8	9
Al_2O_3	8	10
SiO_2	9	11
CaO	7	12
TiO_2	3.05	13
VO_2	0.7	14
V_2O_5	2.2	15
Cr_2O_3	3.4	16
CrO_2	0	17
MnO	3.7	18
Mn_3O_4	2.27	19
Mn_2O_3	4.1	18
MnO_2	1.3	18
Fe_3O_4	0.1	20
Fe_2O_3	2.3	21
CoO	1.95	22
Co_3O_4	1.6	23
NiO	3.4	24
Cu_2O	2.17	24
CuO	1.79	19
ZnO	3.4	24
Ga_2O_3	5.3	25
GeO_2	4.7	25
As_2O_3	4.15	26
SrO	6.14	27
Y_2O_3	6	28
ZrO_2	5	21
NbO_2	0.5	29
Nb_2O_5	3.4	29
MoO_2	2.83	30
MoO_3	3.36	31
RuO_2	2.26	32
Rh_2O_3	1.2	33
PdO	2.13	34
Ag_2O	1.3	35
AgO	1.1	3535
CdO	2.16	3636
In_2O_3	2.9	24
SnO	2.8	24
SnO_2	3.7	25
Sb_2O_3	3.54	37
Sb_2O_5	0.76	37
BaO	3.7	38
La_2O_3	3.61	39
CeO_2	2.8	21
Pr_2O_3	3.7	40
PrO_2	4.5	40
Nd_2O_3	4.1	41
HfO_2	5.8	11
Ta_2O_5	4.5	4242
WO_3	2.8	21
ReO_3	0	43
PtO_2	1.49	44
Tl_2O_3	1.4	45

PbO	2.1	[46]
Pb_3O_4	2.3	[46]
Bi_2O_3	2.91	[47]

Table S3. A collection of relevant turnover frequencies (TOFs) values along with catalytic conditions for thermocatalytic CO_2 hydrogenation over different metal catalysts.

Metal	MO	WF M (eV)	CBM MO (eV)	ΔWF (eV)	new E_F (eV)	E_F - d-band center (eV)	E_C (eV)	Loading (wt.%)	Reaction T (°C)	Metal Particle Size (nm)	TOF (mol CO_2 to products · s^{-1})	Log (TOF)	Main Product	Ref.
Rh	Al_2O_3	4.98	2.28	-2.7	4.98	6.71	-1.73	1.00	150	3.60	0.0009	-3.03621	CH_4	48
Rh	Al_2O_3	4.98	2.28	-2.7	4.98	6.71	-1.73	1.50	150	4.50	0.0012	-2.9393	CH_4	48
Rh	Al_2O_3	4.98	2.28	-2.7	4.98	6.71	-1.73	2.00	150	6.10	0.0023	-2.64207	CH_4	48
Rh	Al_2O_3	4.98	2.28	-2.7	4.98	6.71	-1.73	3.00	150	15.40	0.0033	-2.47756	CH_4	48
Rh	Al_2O_3	4.98	2.28	-2.7	4.98	6.71	-1.73	5.00	150	15.10	0.0020	-2.6925	CH_4	48
Ni	SiO_2	5.2	0.2	-5	5.2	6.49	-1.29	11.80	200	3.50	0.0006	-3.19654	CH_4	49
Ni	SiO_2	5.2	0.2	-5	5.2	6.49	-1.29	6.70	200	2.50	0.0007	-3.17005	CH_4	49
Ni	SiO_2	5.2	0.2	-5	5.2	6.49	-1.29	5.00	200	1.60	0.0008	-3.10458	CH_4	49
Ni	SiO_2	5.2	0.2	-5	5.2	6.49	-1.29	19.50	200	5.00	0.0014	-2.86012	CH_4	49
Ni	SiO_2	5.2	0.2	-5	5.2	6.49	-1.29	1.00	200	1.10	0.0004	-3.45593	CH_4	49
Ni	SiO_2	5.2	0.2	-5	5.2	6.49	-1.29	60.00	200	6.90	0.0037	-2.43415	CH_4	49
Rh	Al_2O_3	4.98	2.28	-2.7	4.98	6.71	-1.73	1.00	200	3.60	0.0172	-1.76447	CH_4	48
Rh	Al_2O_3	4.98	2.28	-2.7	4.98	6.71	-1.73	1.50	200	4.50	0.0124	-1.90658	CH_4	48
Rh	Al_2O_3	4.98	2.28	-2.7	4.98	6.71	-1.73	2.00	200	6.10	0.0152	-1.81816	CH_4	48
Rh	Al_2O_3	4.98	2.28	-2.7	4.98	6.71	-1.73	3.00	200	15.40	0.0188	-1.72584	CH_4	48
Rh	Al_2O_3	4.98	2.28	-2.7	4.98	6.71	-1.73	5.00	200	15.10	0.0119	-1.92445	CH_4	48
Ni	Al_2O_3	5.2	2.28	-2.92	5.2	6.49	-1.29	20.00	220	14.30	0.0005	-3.30103	CH_4	50
Ni	H-Al_2O_3-500	5.2	2.28	-2.92	5.2	6.49	-1.29	20.00	220	6.80	0.0012	-2.92082	CH_4	50
Ni	H-Al_2O_3-400	5.2	2.28	-2.92	5.2	6.49	-1.29	20.00	220	4.60	0.0024	-2.61979	CH_4	50
Cu	SiO_2	4.75	0.2	-4.55	4.75	7.42	-2.67	3.70	230	2.10	0.0006	-3.25964	CH_3OH	51
Ni	Al_2O_3	5.2	2.28	-2.92	5.2	6.49	-1.29	10.00	250	3.70	0.0800	-1.09691	CH_4	52
Ni	ZSM-5	5.2	2.28	-2.92	5.2	6.49	-1.29	10.00	250	14.30	0.0076	-2.1209	CH_4	53
Ni	SBA-15	5.2	0.2	-5	5.2	6.49	-1.29	10.00	250	19.50	0.0059	-2.22768	CH_4	53

Ni	MCM-41	5.2	0.2	-5	5.2	6.49	-1.29	10.00	250	30.30	0.0034	-2.46597	CH_4	[53]
Co	SiO_2	5	0.2	-4.8	5	6.17	-1.17	10.00	250	10.00	0.0070	-2.1549	CO/CH_4	[54]
Ni	SiO_2	5.2	0.2	-5	5.2	6.49	-1.29	11.80	300	3.50	0.0201	-1.6968	CH_4	[49]
Ni	SiO_2	5.2	0.2	-5	5.2	6.49	-1.29	6.70	300	2.50	0.0259	-1.5867	CH_4	[49]
Ni	SiO_2	5.2	0.2	-5	5.2	6.49	-1.29	5.00	300	1.60	0.0245	-1.61083	CH_4	[49]
Ni	SiO_2	5.2	0.2	-5	5.2	6.49	-1.29	19.50	300	5.00	0.0404	-1.39362	CH_4	[49]
Ni	SiO_2	5.2	0.2	-5	5.2	6.49	-1.29	1.00	300	1.10	0.0154	-1.81248	CH_4	[49]
Ni	SiO_2	5.2	0.2	-5	5.2	6.49	-1.29	60.00	300	6.90	0.0403	-1.39469	CH_4	[49]
Ni	Al_2O_3	5.2	2.28	-2.92	5.2	6.49	-1.29	10.00	300	3.70	0.0800	-1.09691	CH_4	[52]
Ni	Al-c	5.2	2.28	-2.92	5.2	6.49	-1.29	15.00	300	5.10	0.0413	-1.38405	CH_4	[55]
Ni	Al-p	5.2	2.28	-2.92	5.2	6.49	-1.29	15.00	300	5.60	0.0970	-1.01323	CH_4	[55]
Co	Al_2O_3	5	2.28	-2.72	5	6.17	-1.17	1.00	300	10.00	0.0105	-1.97881	CO/CH_4	[56]
Pt	SiO_2	5.4	0.2	-5.2	5.4	7.65	-2.25	1.67	300	2.00	0.5800	-0.23657	CO	[57]
Ru	Al_2O_3	4.71	2.28	-2.43	4.71	5.85	-1.14	5.00	300	7.10	0.3800	-0.42022	CH_4	[58]
Pt	SiO_2	5.4	0.2	-5.2	5.4	7.65	-2.25	1.67	310	2.00	0.7400	-0.13077	CO	[57]
Pt	SiO_2	5.4	0.2	-5.2	5.4	7.65	-2.25	1.67	320	2.00	0.9100	-0.04096	CO	[57]
Co	Al_2O_3	5	2.28	-2.72	5	6.17	-1.17	1.00	325	10.00	0.0212	-1.67366	CO/CH_4	[56]
Pt	SiO_2	5.4	0.2	-5.2	5.4	7.65	-2.25	1.67	330	2.00	1.1000	0.041393	CO	[57]
Pt	SiO_2	5.4	0.2	-5.2	5.4	7.65	-2.25	1.67	340	2.00	1.3200	0.120574	CO	[56]
Co	Al_2O_3	5	2.28	-2.72	5	6.17	-1.17	1.00	350	10.00	0.0300	-1.52288	CO/CH_4	[56]
Pt	SiO_2	5.4	0.2	-5.2	5.4	7.65	-2.25	1.67	350	2.00	1.5500	0.190332	CO	[57]
Rh	TiO_2	4.98	7.52	2.54	6.25	7.98	-1.73	0.50	135	2.00	0.0006	-3.18842	CH_4	[59]
Rh	TiO_2	4.98	7.52	2.54	6.25	7.98	-1.73	0.80	135	4.00	0.0017	-2.78252	CH_4	[59]
Rh	TiO_2	4.98	7.52	2.54	6.25	7.98	-1.73	1.00	135	5.00	0.0020	-2.6968	CH_4	[59]
Rh	TiO_2	4.98	7.52	2.54	6.25	7.98	-1.73	2.00	135	7.00	0.0028	-2.54821	CH_4	[59]
Rh	TiO_2	4.98	7.52	2.54	6.25	7.98	-1.73	3.00	135	17.00	0.0053	-2.27491	CH_4	[59]
Rh	TiO_2	4.98	7.52	2.54	6.25	7.98	-1.73	5.00	135	19.00	0.0044	-2.36051	CH_4	[59]
Rh	TiO_2	4.98	7.52	2.54	6.25	7.98	-1.73	0.50	150	2.00	0.0002	-3.6216	CH_4	[59]
Rh	TiO_2	4.98	7.52	2.54	6.25	7.98	-1.73	0.80	150	4.00	0.0008	-3.10403	CH_4	[59]
Rh	TiO_2	4.98	7.52	2.54	6.25	7.98	-1.73	1.00	150	5.00	0.0010	-2.99568	CH_4	[59]

Rh	TiO_2	4.98	7.52	2.54	6.25	7.98	-1.73	2.00	150	7.00	0.0016	-2.7986	CH_4	[59]
Rh	TiO_2	4.98	7.52	2.54	6.25	7.98	-1.73	3.00	150	17.00	0.0033	-2.4828	CH_4	[59]
Rh	TiO_2	4.98	7.52	2.54	6.25	7.98	-1.73	5.00	150	19.00	0.0025	-2.5986	CH_4	[59]
Rh	TiO_2	4.98	7.52	2.54	6.25	7.98	-1.73	0.50	165	2.00	0.0001	-4.0000	CH_4	[59]
Rh	TiO_2	4.98	7.52	2.54	6.25	7.98	-1.73	0.80	165	4.00	0.0004	-3.41341	CH_4	[59]
Rh	TiO_2	4.98	7.52	2.54	6.25	7.98	-1.73	1.00	165	5.00	0.0006	-3.24033	CH_4	[59]
Rh	TiO_2	4.98	7.52	2.54	6.25	7.98	-1.73	2.00	165	7.00	0.0009	-3.03245	CH_4	[59]
Rh	TiO_2	4.98	7.52	2.54	6.25	7.98	-1.73	3.00	165	17.00	0.0018	-2.73755	CH_4	[59]
Rh	TiO_2	4.98	7.52	2.54	6.25	7.98	-1.73	5.00	165	19.00	0.0015	-2.81816	CH_4	[59]
Ni	TiO_2	5.2	7.52	2.32	6.36	7.65	-1.29	5.00	200	9.40	0.0042	-2.37986	CH_4	[60]
Ru	TiO_2	4.71	7.52	2.81	6.115	7.26	-1.14	0.50	200	2.10	0.0150	-1.82391	CH_4	[61]
Ru	TiO_2-200	4.71	7.52	2.81	6.115	7.26	-1.14	2.30	200	1.70	0.0420	-1.37675	CH_4	[62]
Ru	TiO_2-300	4.71	7.52	2.81	6.115	7.26	-1.14	2.30	200	2.20	0.0960	-1.01773	CH_4	[62]
Rh	TiO_2	4.98	7.52	2.54	6.25	7.98	-1.73	6.00	200	2.50	0.0095	-2.02228	CO/CH_4	[63]
Rh	TiO_2	4.98	7.52	2.54	6.25	7.98	-1.73	0.50	200	0.94	0.0137	-1.86328	CO/CH_4	[63]
Rh	TiO_2	4.98	7.52	2.54	6.25	7.98	-1.73	2.00	200	1.40	0.0178	-1.74958	CO/CH_4	[63]
Rh	TiO_2	4.98	7.52	2.54	6.25	7.98	-1.73	3.00	200	2.10	0.0215	-1.66756	CO/CH_4	[63]
Rh	TiO_2	4.98	7.52	2.54	6.25	7.98	-1.73	4.00	200	1.60	0.0191	-1.71897	CO/CH_4	[63]
Rh	TiO_2	4.98	7.52	2.54	6.25	7.98	-1.73	6.00	200	2.50	0.0232	-1.63451	CO/CH_4	[63]
Ni	TiO_2	5.2	7.52	2.32	6.36	7.65	-1.29	5.00	225	9.40	0.0056	-2.25493	CH_4	[60]
Ru	TiO_2	4.71	7.52	2.81	6.115	7.26	-1.14	0.50	225	2.10	0.0327	-1.48545	CH_4	[61]
Ru	TiO_2	4.71	7.52	2.81	6.115	7.26	-1.14	0.50	225	2.10	0.0368	-1.43415	CH_4	[61]
Cu	ZrO_2	4.75	6.86	2.11	5.805	8.48	-2.67	0.80	230	2.20	0.0016	-2.79588	CH_3OH	[51]
Ni	ZrO_2-P	5.2	6.86	1.66	6.03	7.32	-1.29	8.70	235	9.30	0.0580	-1.23657	CH_4	[64]
Ni	ZrO_2-C	5.2	6.86	1.66	6.03	7.32	-1.29	10.00	235	16.00	0.0400	-1.39794	CH_4	[64]
Ni	TiO_2	5.2	7.52	2.32	6.36	7.65	-1.29	5.00	250	9.40	0.0106	-1.97469	CH_4	[60]
Co	TiO_2	5	7.52	2.52	5	6.17	-1.17	10.00	250	10.00	0.0200	-1.69897	CO/CH_4	[54]
Cu	TiO_2-x-500	4.75	7.52	2.77	6.135	8.81	-2.67	5.90	250	5.50	0.0141	-1.85078	CH_3OH	[65]
Cu	TiO_2	4.75	7.52	2.77	6.135	8.81	-2.67	5.90	250	5.00	0.0017	-2.76955	CH_3OH	[65]

Ru	TiO_2	4.71	7.52	2.81	6.115	7.26	-1.14	0.50	250	2.10	0.0590	-1.22915	CH_4	[61]
Ru	TiO_2	4.71	7.52	2.81	6.115	7.26	-1.14	0.50	250	2.10	0.0657	-1.18243	CH_4	[61]
Cu	TiO_2-x-500	4.75	7.52	2.77	6.135	8.81	-2.67	5.90	300	5.50	0.0260	-1.58503	CH_3OH	[65]
Cu	TiO_2	4.75	7.52	2.77	6.135	8.81	-2.67	5.90	300	5.00	0.0029	-2.5376	CH_3OH	[65]
Pt	TiO_2	5.4	7.52	2.12	6.46	8.71	-2.25	1.67	300	1.00	2.7600	0.440909	CO	[57]
Pt	TiO_2	5.4	7.52	2.12	6.46	8.71	-2.25	1.67	310	1.00	3.3100	0.519828	CO	[57]
Fe	ZrO_2	4.7	6.86	2.16	5.78	6.70	-0.92	10.00	320	12.90	0.0450	-1.34679	CO/CH_4	[66]
Fe	ZrO_2	4.7	6.86	2.16	5.78	6.70	-0.92	10.00	320	9.80	0.0200	-1.69897	CO/CH_4	[66]
Fe	ZrO_2	4.7	6.86	2.16	5.78	6.70	-0.92	10.00	320	6.10	0.0090	-2.04576	CO/CH_4	[66]
Fe	ZrO_2	4.7	6.86	2.16	5.78	6.70	-0.92	10.00	320	4.00	0.0060	-2.22185	CO/CH_4	[66]
Fe	ZrO_2	4.7	6.86	2.16	5.78	6.70	-0.92	10.00	320	2.50	0.0040	-2.39794	CO/CH_4	[66]
Fe	ZrO_2	4.7	6.86	2.16	5.78	6.70	-0.92	10.00	320	2.50	0.0001	-3.85387	CO/CH_4	[66]
Fe	ZrO_2	4.7	6.86	2.16	5.78	6.70	-0.92	10.00	320	2.50	0.0001	-3.85387	CO/CH_4	[66]
Fe	ZrO_2	4.7	6.86	2.16	5.78	6.70	-0.92	10.00	320	2.50	0.0001	-3.85387	CO/CH_4	[66]
Pt	TiO_2	5.4	7.52	2.12	6.46	8.71	-2.25	1.67	320	1.00	3.9800	0.599883	CO	[57]
Ru	TiO_2	4.71	7.52	2.81	6.115	7.26	-1.14	0.50	320	4.20	0.2580	-0.58838	CH_4	[61]
Cu	TiO_2-x-500	4.75	7.52	2.77	6.135	8.81	-2.67	5.90	325	5.50	0.0206	-1.68613	CH_3OH	[65]
Cu	TiO_2	4.75	7.52	2.77	6.135	8.81	-2.67	5.90	325	5.00	0.0023	-2.63827	CH_3OH	[65]
Pt	TiO_2	5.4	7.52	2.12	6.46	8.71	-2.25	1.67	330	1.00	4.6700	0.669317	CO	[57]
Pt	TiO_2	5.4	7.52	2.12	6.46	8.71	-2.25	1.67	340	1.00	5.4500	0.736397	CO	[57]
Pt	TiO_2	5.4	7.52	2.12	6.46	8.71	-2.25	1.67	350	1.00	6.2600	0.796574	CO	[57]
Pt	TiO_2	5.4	7.52	2.12	6.46	8.71	-2.25	0.50	350	1.70	0.0855	-1.06803	CO	[67]
Pt	TiO_2	5.4	7.52	2.12	6.46	8.71	-2.25	0.50	350	2.25	0.0825	-1.08355	CO	[67]
Pt	TiO_2	5.4	7.52	2.12	6.46	8.71	-2.25	0.50	350	3.05	0.0920	-1.03621	CO	[67]
Pt	TiO_2	5.4	7.52	2.12	6.46	8.71	-2.25	0.50	350	1.50	0.1050	-0.97881	CO	[67]
Ru	TiO_2	4.71	7.52	2.81	6.115	7.26	-1.14	0.50	350	4.20	0.4260	-0.37059	CH_4	[61]
Ir	TiO_2	5.3	7.52	2.22	6.41	8.52	-2.11	0.50	350	1.50	0.0220	-1.65758	CO	[68]
Ir	TiO_2	5.3	7.52	2.22	6.41	8.52	-2.11	1.00	350	1.10	0.0150	-1.82391	CO	[68]
Ir	TiO_2	5.3	7.52	2.22	6.41	8.52	-2.11	5.00	350	2.00	0.0013	-2.88606	CO/CH_4	[68]

References

(1) Wan, Z.; Wang, Q.-D.; Liu, D.; Liang, J. Data-driven machine learning model for the prediction of oxygen vacancy formation energy of metal oxide materials. *Phys. Chem. Chem. Phys.* **2021**, *23*, 15675-15684.
(2) Deml, A. M.; Holder, A. M.; O'Hayre, R. P.; Musgrave, C. B.; Stevanović, V. Intrinsic Material Properties Dictating Oxygen Vacancy Formation Energetics in Metal Oxides. *J. Phys. Chem. Lett.* **2015**, *6*, 1948-1953.
(3) Carey, J. J.; Legesse, M.; Nolan, M. Low Valence Cation Doping of Bulk Cr_2O_3: Charge Compensation and Oxygen Vacancy Formation. *J. Phys. Chem. C* **2016**, *120*, 19160-19174.
(4) Liu, S.; Lu, Z.; Yuan, C.; Guo, F.; Xiong, R.; Shi, J. Oxygen Vacancies in CrO_2: The Influences to Half Metallicity and the Formation Mode. *IEEE Trans. Magn.* **2016**, *52*, 1-8.
(5) Zhang, M.; Duan, X.; Gao, Y.; Zhang, S.; Lu, X.; Luo, K.; Ye, J.; Wang, X.; Niu, Q.; Zhang, P. Tuning Oxygen Vacancies in Oxides by Configurational Entropy. *ACS Appl. Mater. Interfaces* **2023**, *15*, 45774-45789.
(6) Hinuma, Y.; Toyao, T.; Kamachi, T.; Maeno, Z.; Takakusagi, S.; Furukawa, S.; Takigawa, I.; Shimizu, K.-i. Density Functional Theory Calculations of Oxygen Vacancy Formation and Subsequent Molecular Adsorption on Oxide Surfaces. *J. Phys. Chem. C* **2018**, *122*, 29435-29444.
(7) Chatten, R.; Chadwick, A. V.; Rougier, A.; Lindan, P. J. The oxygen vacancy in crystal phases of WO_3. *J. Phys. Chem. B* **2005**, *109*, 3146-3156.
(8) Li, D.; Ching, W. Electronic structures and optical properties of low-and high-pressure phases of crystalline B_2O_3. *Phys. Rev. B* **1996**, *54*, 13616.
(9) Patel, M. K.; Ali, M. A.; Krishnan, S.; Agrawal, V. V.; Al Kheraif, A. A.; Fouad, H.; Ansari, Z.; Ansari, S.; Malhotra, B. D. A label-free photoluminescence genosensor using nanostructured magnesium oxide for cholera detection. *Sci. Rep.* **2015**, *5*, 17384.
(10) Chen, Y.; Bovet, N.; Trier, F.; Christensen, D.; Qu, F.; Andersen, N. H.; Kasama, T.; Zhang, W.; Giraud, R.; Dufouleur, J. A high-mobility two-dimensional electron gas at the spinel/perovskite interface of γ-Al_2O_3/$SrTiO_3$. *Nat. Commun.* **2013**, *4*, 1371.
(11) Illarionov, Y. Y.; Knobloch, T.; Jech, M.; Lanza, M.; Akinwande, D.; Vexler, M. I.; Mueller, T.; Lemme, M. C.; Fiori, G.; Schwierz, F. Insulators for 2D nanoelectronics: the gap to bridge. *Nat. Commun.* **2020**, *11*, 3385.
(12) Whited, R.; Flaten, C. J.; Walker, W. Exciton thermoreflectance of MgO and CaO. *Solid State Commun.* **1973**, *13*, 1903-1905.
(13) Carp, O.; Huisman, C. L.; Reller, A. Photoinduced reactivity of titanium dioxide. *Prog. Solid State Chem.* **2004**, *32*, 33-177.
(14) Hu, P.; Hu, P.; Vu, T. D.; Li, M.; Wang, S.; Ke, Y.; Zeng, X.; Mai, L.; Long, Y. Vanadium Oxide: Phase Diagrams, Structures, Synthesis, and Applications. *Chem. Rev.* **2023**, *123*, 4353-4415.
(15) Broomhead, W. T.; Tian, W.; Herrera, J. E.; Chin, Y.-H. C. Kinetic Coupling of Redox and Acid Chemistry in Methanol Partial Oxidation on Vanadium Oxide Catalysts. *ACS Catal.* **2022**, *12*, 11801-11820.
(16) Ding, C.; Ma, Y.; Lai, X.; Yang, Q.; Xue, P.; Hu, F.; Geng, W. Ordered large-pore mesoporous Cr_2O_3 with ultrathin framework for formaldehyde sensing. *ACS Appl. Mater. Interfaces* **2017**, *9*, 18170-18177.
(17) Dwivedi, S.; Biswas, S. Enhanced room-temperature magnetoresistance in self-assembled Ag-coated multiphasic chromium oxide nanocomposites. *Phys. Chem. Chem. Phys.* **2016**, *18*, 23879-23887.
(18) Ghosh, S. K. Diversity in the family of manganese oxides at the nanoscale: from fundamentals to applications. *ACS omega* **2020**, *5*, 25493-25504.
(19) Gupta, S. V.; Kulkarni, V. V.; Ahmaruzzaman, M. Bandgap engineering approach for designing $CuO/Mn_3O_4/CeO_2$ heterojunction as a novel photocatalyst for AOP-assisted degradation of Malachite green dye. *Sci. Rep.* **2023**, *13*, 3009.
(20) Ashraf, M.; Khan, I.; Usman, M.; Khan, A.; Shah, S. S.; Khan, A. Z.; Saeed, K.; Yaseen, M.; Ehsan, M. F.; Tahir, M. N. Hematite and magnetite nanostructures for green and sustainable energy harnessing and environmental pollution control: a review. *Chem. Res. Toxicol.* **2019**, *33*, 1292-1311.
(21) Medhi, R.; Marquez, M. D.; Lee, T. R. Visible-light-active doped metal oxide nanoparticles: review of their synthesis, properties, and applications. *ACS Appl. Nano Mater.* **2020**, *3*, 6156-6185.
(22) Moridon, S. N. F.; Salehmin, M. I.; Mohamed, M. A.; Arifin, K.; Minggu, L. J.; Kassim, M. B. Cobalt oxide as photocatalyst for water splitting: Temperature-dependent phase structures. *Int. J. Hydrog. Energy* **2019**, *44*, 25495-25504.
(23) Chen, J.; Wu, X.; Selloni, A. Electronic structure and bonding properties of cobalt oxide in the spinel structure. *Phys. Rev. B* **2011**, *83*, 245204.
(24) Shi, J.; Zhang, J.; Yang, L.; Qu, M.; Qi, D. C.; Zhang, K. H. Wide bandgap oxide semiconductors: from materials physics to optoelectronic devices. *Adv. Mater.* **2021**, *33*, 2006230.

(25) Wong, M. H.; Bierwagen, O.; Kaplar, R. J.; Umezawa, H. Ultrawide-bandgap semiconductors: An overview. *J. Mater. Res.* **2021**, *36*, 4601-4615.

(26) Dhaka, R.; Yadav, A.; Goyal, A.; Pandey, A.; Gupta, G.; Dutta, S.; Shukla, A. Structural and Optical Properties of Arsenic-Oxide Microcrystals on Gaas Substrate for Photonic Applications. *Available at SSRN 4512709*. Dhaka, Rangeeta and Yadav, Aditya and Goyal, Anshu and Pandey, Akhilesh and Gupta, Govind and Dutta, Shankar and Shukla, A. K., Structural and Optical Properties of Arsenic-Oxide Microcrystals on Gaas Substrate for Photonic Applications. Available at SSRN: http://dx.doi.org/10.2139/ssrn.4512709

(27) Nemade, K.; Waghuley, S. UV–VIS spectroscopic study of one pot synthesized strontium oxide quantum dots. *Results Phys.* **2013**, *3*, 52-54.

(28) Tropf, W. J.; Thomas, M. E. *Yttrium oxide (Y_2O_3)* In *Handbook of optical constants of solids*; Elsevier, Amsterdam, 1997.

(29) Nico, C.; Monteiro, T.; Graça, M. P. Niobium oxides and niobates physical properties: Review and prospects. *Prog. Mater. Sci.* **2016**, *80*, 1-37.

(30) Patil, R.; Uplane, M.; Patil, P. Structural and optical properties of electrodeposited molybdenum oxide thin films. *Appl. Surf. Sci.* **2006**, *252*, 8050-8056.

(31) Kumar, A.; Pandey, G. Synthesis, characterization, effect of temperature on band gap energy of molybdenum oxide nano rods and their antibacterial activity. *Am. J. Appl. Ind. Chem.* **2017**, *3*, 38-42.

(32) Vijayabala, V.; Senthilkumar, N.; Nehru, K.; Karvembu, R. Hydrothermal synthesis and characterization of ruthenium oxide nanosheets using polymer additive for supercapacitor applications. *J. Mater. Sci.: Mater. Electron.* **2018**, *29*, 323-330.

(33) Scherson, Y. D.; Aboud, S. J.; Wilcox, J.; Cantwell, B. J. Surface structure and reactivity of rhodium oxide. *J. Phys. Chem. C* **2011**, *115*, 11036-11044.

(34) Rey, E.; Kamal, M.; Miles, R.; Royce, B. The semiconductivity and stability of palladium oxide. *J. Mater. Sci.* **1978**, *13*, 812-816.

(35) Allen, J. P.; Scanlon, D. O.; Watson, G. W. Electronic structures of silver oxides. *Phys. Rev. B* **2011**, *84*, 115141.

(36) Jefferson, P. H.; Hatfield, S.; Veal, T. D.; King, P.; McConville, C. F.; Zúñiga–Pérez, J.; Muñoz–Sanjosé, V. Bandgap and effective mass of epitaxial cadmium oxide. *Appl. Phys. Lett.* **2008**, *92*.

(37) Allen, J. P.; Carey, J. J.; Walsh, A.; Scanlon, D. O.; Watson, G. W. Electronic structures of antimony oxides. *J. Phys. Chem. C* **2013**, *117*, 14759-14769.

(38) Sundharam, E.; Jeevaraj, A.; Chinnusamy, C. Effect of ultrasonication on the synthesis of barium oxide nanoparticles. *J. Bionanosci.* **2017**, *11*, 310-314.

(39) Gu, W.; Song, Y.; Liu, J.; Wang, F. Lanthanum-Based Compounds: Electronic Band-Gap-Dependent Electrocatalytic Materials for Oxygen Reduction Reaction. *Chem. Eur. J.* **2017**, *23*, 10126-10132.

(40) Seifarth, O.; Dabrowski, J.; Zaumseil, P.; Müller, S.; Schmeißer, D.; Müssig, H.-J.; Schroeder, T. On the band gaps and electronic structure of thin single crystalline praseodymium oxide layers on Si (111). *J. Vac. Sci. Technol. B:Nanotechnol. Microelectron.* **2009**, *27*, 271-276.

(41) Pourmortazavi, S. M.; Rahimi-Nasrabadi, M.; Aghazadeh, M.; Ganjali, M. R.; Karimi, M. S.; Norouzi, P. Synthesis, characterization and photocatalytic activity of neodymium carbonate and neodymium oxide nanoparticles. *J. Mol. Struct.* **2017**, *1150*, 411-418.

(42) Kukli, K.; Aarik, J.; Aidla, A.; Kohan, O.; Uustare, T.; Sammelselg, V. Properties of tantalum oxide thin films grown by atomic layer deposition. *Thin Solid Films* **1995**, *260*, 135-142.

(43) Hahn, B. P.; May, R. A.; Stevenson, K. J. Electrochemical deposition and characterization of mixed-valent rhenium oxide films prepared from a perrhenate solution. *Langmuir* **2007**, *23*, 10837-10845.

(44) Seriani, N.; Jin, Z.; Pompe, W.; Ciacchi, L. C. Density functional theory study of platinum oxides: From infinite crystals to nanoscopic particles. *Phys. Rev. B* **2007**, *76*, 155421.

(45) Moeinian, M.; Akhbari, K. Various methods for synthesis of bulk and nano thallium (III) oxide. *J. Inorg. Organomet. Polym. Mater.* **2016**, *26*, 1-13.

(46) Zhuravlev, Y. N.; Korabel'nikov, D. V. e. A first principles study of the mechanical, electronic, and vibrational properties of lead oxide. *Phys. Solid State* **2017**, *59*, 2296-2311.

(47) Ho, C.-H.; Chan, C.-H.; Huang, Y.-S.; Tien, L.-C.; Chao, L.-C. The study of optical band edge property of bismuth oxide nanowires α-Bi_2O_3. *Opt. Express* **2013**, *21*, 11965-11972.

(48) Karelovic, A.; Ruiz, P. CO_2 hydrogenation at low temperature over Rh/γ-Al_2O_3 catalysts: Effect of the metal particle size on catalytic performances and reaction mechanism. *Appl. Catal. B: Environ.* **2012**, *113-114*, 237-249.

(49) Vogt, C.; Groeneveld, E.; Kamsma, G.; Nachtegaal, M.; Lu, L.; Kiely, C. J.; Berben, P. H.; Meirer, F.; Weckhuysen, B. M. Unravelling structure sensitivity in CO_2 hydrogenation over nickel. *Nat. Catal.* **2018**, *1*, 127-134.

(50) He, S.; Li, C.; Chen, H.; Su, D.; Zhang, B.; Cao, X.; Wang, B.; Wei, M.; Evans, D. G.; Duan, X. A surface defect-promoted Ni nanocatalyst with simultaneously enhanced activity and stability. *Chemistry of Materials* **2013**, *25*, 1040-1046.

(51) Larmier, K.; Liao, W.-C.; Tada, S.; Lam, E.; Verel, R.; Bansode, A.; Urakawa, A.; Comas-Vives, A.; Copéret, C. CO_2-to-Methanol Hydrogenation on Zirconia-Supported Copper Nanoparticles: Reaction

Intermediates and the Role of the Metal–Support Interface. *Angew. Chem. Int. Ed.* **2017**, *56*, 2318-2323.
(52) Mutz, B.; Gänzler, A. M.; Nachtegaal, M.; Müller, O.; Frahm, R.; Kleist, W.; Grunwaldt, J.-D. Surface oxidation of supported Ni particles and its impact on the catalytic performance during dynamically operated methanation of CO_2. *Catalysts* **2017**, *7*, 279.
(53) Guo, X.; Traitangwong, A.; Hu, M.; Zuo, C.; Meeyoo, V.; Peng, Z.; Li, C. Carbon dioxide methanation over nickel-based catalysts supported on various mesoporous material. *Energy & Fuels* **2018**, *32*, 3681-3689.
(54) Melaet, G. r. m.; Ralston, W. T.; Li, C.-S.; Alayoglu, S.; An, K.; Musselwhite, N.; Kalkan, B.; Somorjai, G. A. Evidence of highly active cobalt oxide catalyst for the Fischer–Tropsch synthesis and CO_2 hydrogenation. *J.Am. Chem. Soc.* **2014**, *136*, 2260-2263.
(55) Bian, L.; Zhang, L.; Xia, R.; Li, Z. Enhanced low-temperature CO_2 methanation activity on plasma-prepared Ni-based catalyst. *J. Nat. Gas Eng.* **2015**, *27*, 1189-1194.
(56) Shin, H. H.; Lu, L.; Yang, Z.; Kiely, C. J.; McIntosh, S. Cobalt catalysts decorated with platinum atoms supported on barium zirconate provide enhanced activity and selectivity for CO_2 methanation. *ACS Catal.* **2016**, *6*, 2811-2818.
(57) Kattel, S.; Yan, B.; Chen, J. G.; Liu, P. CO2 hydrogenation on Pt, Pt/SiO_2 and Pt/TiO_2: Importance of synergy between Pt and oxide support. *J. Catal.* **2016**, *343*, 115-126.
(58) Dreyer, J. A. H.; Li, P.; Zhang, L.; Beh, G. K.; Zhang, R.; Sit, P. H. L.; Teoh, W. Y. Influence of the oxide support reducibility on the CO_2 methanation over Ru-based catalysts. *Appl. Catal. B: Environ.* **2017**, *219*, 715-726.
(59) Karelovic, A.; Ruiz, P. Mechanistic study of low temperature CO_2 methanation over Rh/TiO_2 catalysts. *J. Catal.* **2013**, *301*, 141-153.
(60) Li, M.; Amari, H.; van Veen, A. C. Metal-oxide interaction enhanced CO_2 activation in methanation over ceria supported nickel nanocrystallites. *Appl. Catal. B: Environ.* **2018**, *239*, 27-35.
(61) Panagiotopoulou, P. Methanation of CO_2 over alkali-promoted Ru/TiO_2 catalysts: II. Effect of alkali additives on the reaction pathway. *Appl. Catal. B: Environ.* **2018**, *236*, 162-170.
(62) Zhang, Y.; Yan, W.; Qi, H.; Su, X.; Su, Y.; Liu, X.; Li, L.; Yang, X.; Huang, Y.; Zhang, T. Strong Metal–Support Interaction of Ru on TiO_2 Derived from the Co-Reduction Mechanism of Ru x $Ti_{1-x}O_2$ Interphase. *ACS Catal.* **2022**, *12*, 1697-1705.
(63) Matsubu, J. C.; Yang, V. N.; Christopher, P. Isolated metal active site concentration and stability control catalytic CO_2 reduction selectivity. *J.Am. Chem. Soc.* **2015**, *137*, 3076-3084.
(64) Jia, X.; Zhang, X.; Rui, N.; Hu, X.; Liu, C.-j. Structural effect of Ni/ZrO_2 catalyst on CO_2 methanation with enhanced activity. *Appl. Catal. B: Environ.* **2019**, *244*, 159-169.
(65) Zhang, C.; Wang, L.; Etim, U. J.; Song, Y.; Gazit, O. M.; Zhong, Z. Oxygen vacancies in Cu/TiO_2 boost strong metal-support interaction and CO_2 hydrogenation to methanol. *J. Catal.* **2022**, *413*, 284-296.
(66) Zhu, J.; Zhang, G.; Li, W.; Zhang, X.; Ding, F.; Song, C.; Guo, X. Deconvolution of the particle size effect on CO_2 hydrogenation over iron-based catalysts. *ACS Catal.* **2020**, *10*, 7424-7433.
(67) Chen, Z.; Liang, L.; Yuan, H.; Liu, H.; Wu, P.; Fu, M.; Wu, J.; Chen, P.; Qiu, Y.; Ye, D. Reciprocal regulation between support defects and strong metal-support interactions for highly efficient reverse water gas shift reaction over Pt/TiO_2 nanosheets catalysts. *Appl. Catal. B: Environ.* **2021**, *298*, 120507.
(68) Chen, X.; Su, X.; Su, H.-Y.; Liu, X.; Miao, S.; Zhao, Y.; Sun, K.; Huang, Y.; Zhang, T. Theoretical insights and the corresponding construction of supported metal catalysts for highly selective CO_2 to CO conversion. *ACS Catal.* **2017**, *7*, 4613-4620.
